# Novel Potent Muscarinic Receptor Antagonists: Investigation on the Nature of Lipophilic Substituents in the 5- and/or 6-Positions of the 1,4-Dioxane Nucleus

**DOI:** 10.1021/acs.jmedchem.9b02100

**Published:** 2020-05-06

**Authors:** Fabio Del Bello, Alessandro Bonifazi, Gianfabio Giorgioni, Alessandro Piergentili, Maria Giovanna Sabbieti, Dimitrios Agas, Marzia Dell’Aera, Rosanna Matucci, Marcin Górecki, Gennaro Pescitelli, Giulio Vistoli, Wilma Quaglia

**Affiliations:** † Scuola di Scienze del Farmaco e dei Prodotti della Salute, Università di Camerino, Via S. Agostino 1, 62032 Camerino, Italy; ‡ Scuola di Bioscienze e Medicina Veterinaria, Università di Camerino, Via Gentile III da Varano, 62032 Camerino, Italy; § Istituto di Cristallografia IC-CNR, Via Amendola 122/o, 70126 Bari, Italy; ∥ Dipartimento di Farmacia-Scienze del Farmaco, Università di Bari “A. Moro”, Consorzio C.I.N.M.P.I.S., Via E. Orabona 4, I-70125 Bari, Italy; ⊥ Dipartimento di Neuroscienze, Psicologia, Area del Farmaco e Salute del Bambino (NEUROFARBA), Sezione di Farmacologia e Tossicologia, Università degli Studi di Firenze, Viale Pieraccini 6, 50139 Firenze, Italy; # Dipartimento di Chimica e Chimica Industriale, Università di Pisa, Via Moruzzi 13, 56124 Pisa, Italy; ¶ Institute of Organic Chemistry, Polish Academy of Sciences, Kasprzaka 44/52 Street, 01-224 Warsaw, Poland; ∇ Dipartimento di Scienze Farmaceutiche, Università degli Studi di Milano, Via Mangiagalli 25, 20133 Milano, Italy

## Abstract

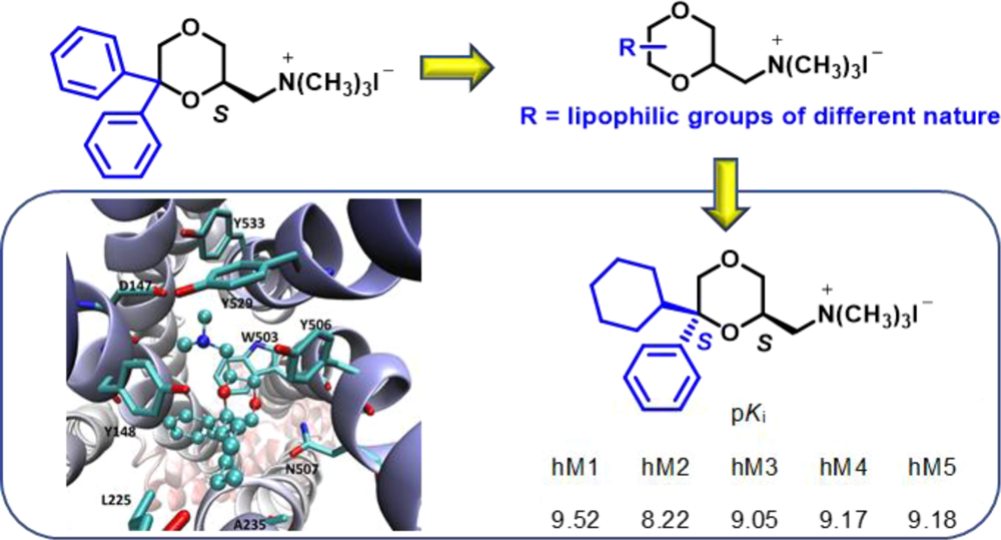

A series of novel 1,4-dioxane analogues of the muscarinic acetylcholine receptor (mAChR) antagonist **2** was synthesized and studied for their affinity at M_1_–M_5_ mAChRs. The 6-cyclohexyl-6-phenyl derivative **3b**, with a *cis* configuration between the CH_2_N^+^(CH_3_)_3_ chain in the 2-position and the cyclohexyl moiety in the 6-position, showed p*K*_i_ values for mAChRs higher than those of **2** and a selectivity profile analogous to that of the clinically approved drug oxybutynin. The study of the enantiomers of **3b** and the corresponding tertiary amine **33b** revealed that the eutomers are (2*S*,6*S*)-(−)-**3b** and (2*S*,6*S*)-(−)-**33b**, respectively. Docking simulations on the M_3_ mAChR-resolved structure rationalized the experimental observations. The quaternary ammonium function, which should prevent the crossing of the blood–brain barrier, and the high M_3_/M_2_ selectivity, which might limit cardiovascular side effects, make **3b** a valuable starting point for the design of novel antagonists potentially useful in peripheral diseases in which M_3_ receptors are involved.

## Introduction

Muscarinic acetylcholine receptors (mAChRs) are proteins with seven transmembrane domains separated by intracellular and extracellular loops. Acetylcholine binds to the extracellular region of mAChRs and thereafter activates GTP-binding regulatory proteins in the intracellular compartment. The mAChR family consists of five closely related members (M_1_–M_5_). M_1_, M_3_, and M_5_ mAChRs are associated with G_q/11_ proteins to trigger phospholipase-C activation. Their activation increases neuronal excitability through the opening of nonspecific cation channels, mobilization of intracellular Ca^2+^, or inhibition of small-conductance Ca^2+^-activated K^+^ channels. M_2_ and M_4_ subtypes couple to G_i/o_ proteins, inhibiting adenylate cyclase and reducing the levels of intracellular adenosine 3′,5′-cyclic monophosphate (cAMP).^1^ mAChRs mediate several functions in the central nervous system (CNS), where they play a crucial role in cognitive functions^2^ and pain circuits.^3^ Moreover, in the periphery, M_2_ and/or M_3_ subtypes are involved in smooth muscle contraction,^4^ cardiovascular function,^5^ and glandular secretion.^6^ Acetylcholine is not only a neurotransmitter but can also act on non-neuronal cells, and the muscarinic system is involved in the regulation of stem^7^ and cancer cells,^8^ in immunity and inflammation,^9^ and in the mucocutaneous epithelial barrier.^10^ Moreover, muscarinic signals have been demonstrated to be transmitted by mesenchymal stem cells (MSCs) from different tissues.^11,12^

**Figure 1:**
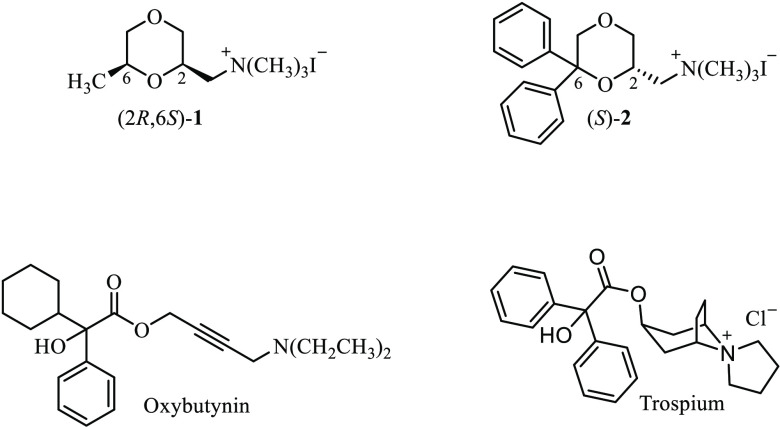
Chemical structures of (2*R*,6*S*)-**1**, (*S*)-**2**, oxybutynin, and trospium.

The 1,4-dioxane nucleus has been demonstrated to be a versatile scaffold for the development of compounds interacting with different receptor systems,^13−20^ including mAChRs.^21−25^ We have demonstrated that the size of the substituent in the 6-position affects the functional activity of 1,4-dioxane ligands directed to mAChRs.^24^ Indeed, a methyl group in this position led to the effective agonist (2*R*,6*S*)-**1**,^23^ whereas aromatic rings characterized potent antagonists, such as the 6,6-diphenyl derivative (*S*)-**2**^24^ (Figure 1). In *in vivo* studies in anesthetized rats, compared to oxybutynin (Figure 1), an antagonist clinically used for overactive bladder (OAB) treatment,^26^ (*S*)-**2** more efficaciously reduced the volume-induced contractions of the urinary bladder.

**Figure 2:**
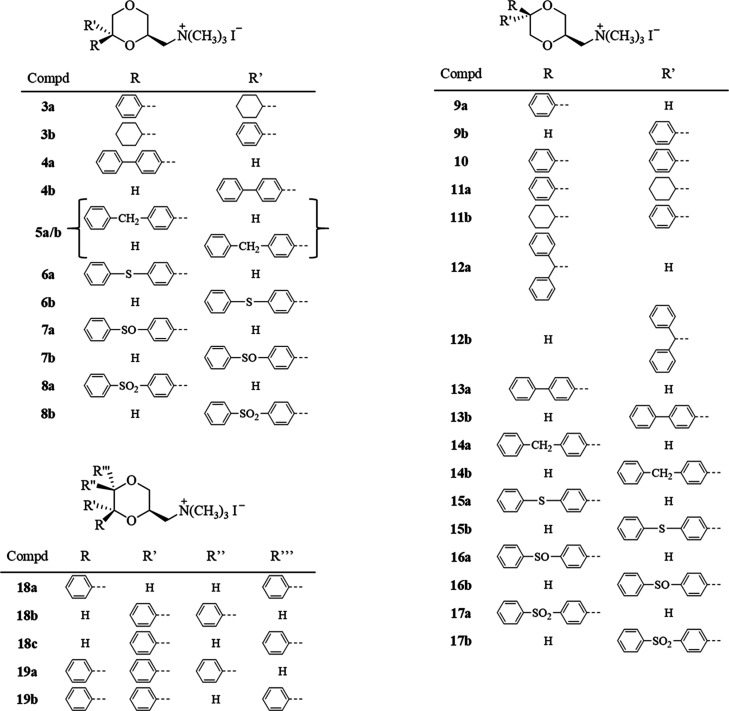
Chemical structures of the new 1,4-dioxane derivatives **3–19**. Only one enantiomer of the racemic mixture is shown.

In the effort to obtain novel potent mAChR antagonists preferentially targeting peripheral M_3_ subtype and potentially useful for the treatment of OAB, the diphenyl group in the 6-position of compound **2** has been replaced by different lipophilic groups (compounds **3–8**, Figure 2). Furthermore, the lipophilic moiety has been moved from the 6- to 5-position (compounds **9–17**, Figure 2) or introduced in both 5- and 6-positions of the 1,4-dioxane ring (compounds **18–19**, Figure 2). All the substituents are aromatic groups, except for the aliphatic cyclohexyl ring in compounds **3** and **11**, which has been selected because it is present in several potent mAChR antagonists, including oxybutynin (Figure 1).

Owing to the lack of M_3_ subtype selectivity, the muscarinic compounds used in therapy for OAB show cardiovascular side effects, due to the interaction with peripheral M_2_ subtype, and/or cognitive side effects, due to the blockade of central mAChRs.^27,28^ Among these drugs, only trospium bears a hydrophilic quaternary ammonium head (Figure 1) that prevents the crossing of the blood–brain barrier (BBB), thus minimizing CNS side effects.^29^

Because of the high degree of amino acid sequence homology in the orthosteric site of the M_1_–M_5_ mAChR subtypes, it is very difficult to obtain orthosteric ligands selective for M_3_ mAChR over all the other subtypes. For this reason, the aim of the present study was to confine the activity of the novel compounds to peripheral tissues, minimizing CNS side effects and, hopefully, to limit cardiovascular side effects by improving the M_3_/M_2_ selectivity ratio. Therefore, the new molecules have been designed to have a quaternary ammonium function that should prevent the crossing of the BBB.

The novel compounds will provide further information on the role played by the lipophilic moiety in the interaction with the five mAChR subtypes.

Moreover, considering the pivotal role played by stereochemistry in the interaction of both 1,4-dioxane agonists and antagonists with the five mAChR subtypes,^22,24,30^ the enantiomeric resolution of the most potent compound **3b** was performed. The absolute configuration of the enantiomers of **3b** was determined by quantum mechanical simulations of electronic circular dichroism (ECD). To elucidate the binding mode of the described compounds and to rationalize the biological results, docking simulations on the M_3_ mAChR-resolved structure were performed.

## Results and Discussion

### Chemistry

**Scheme 1:**
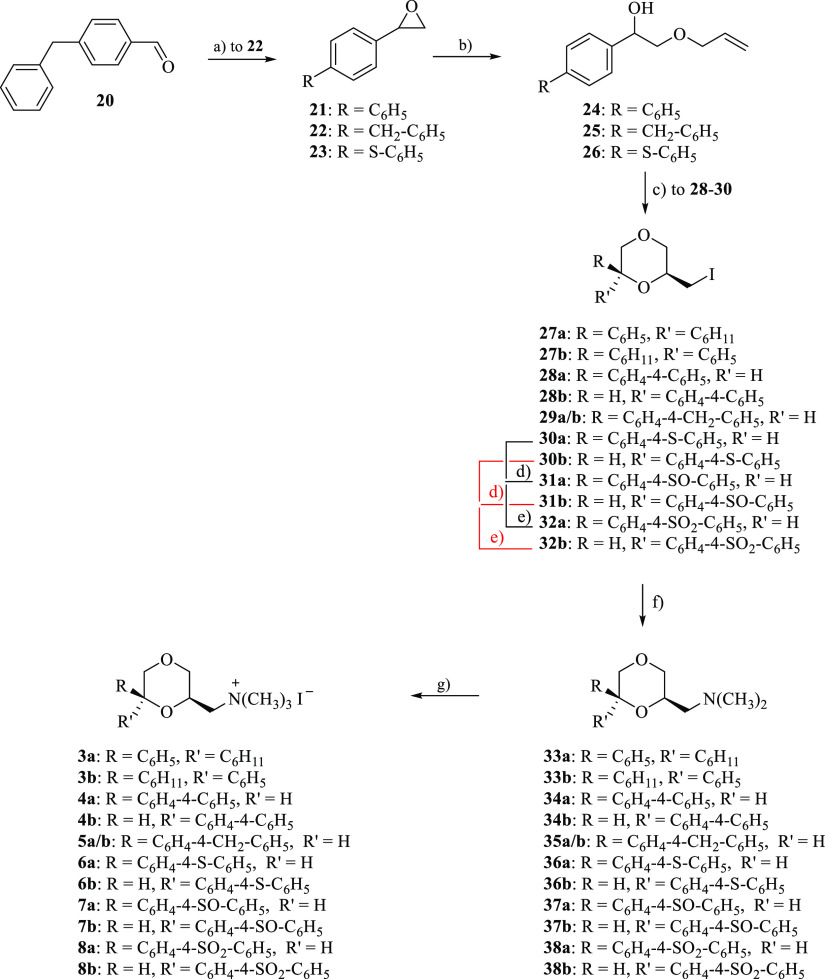
Reagents: (a) (CH_3_)_3_SI, NaH, DMSO; (b) Na, CH_2_=CHCH_2_OH; (c) (CH_3_COO)_2_Hg; H_2_O, KI, I_2_; (d) 1 equiv *m*-CPBA, CH_2_Cl_2_, 30 min; (e) 2 equiv *m*-CPBA, CH_2_Cl_2_, 2 h; (f) (CH_3_)_2_NH, C_6_H_6_; (g) CH_3_I, (CH_3_CH_2_)_2_O Only one enantiomer of the racemic mixture is shown.

Compounds **3–8** were synthesized following the procedure reported in Scheme 1 and were obtained as racemates. 4-Benzylbenzaldehyde **20**(31) was converted into the oxirane **22** by reaction with sodium hydride and trimethylsulfonium iodide in dimethyl sulfoxide (DMSO), according to the procedure reported by Corey and Chaykovsky.^32^ The opening of oxiranes **21**,^33^**22**, and **23**(34) with allyl alcohol in the presence of Na gave alkenes **24–26**, respectively. The mixtures of diastereomers **28–30** were obtained by the oxymercuration-reduction reaction with mercury(II) acetate and subsequent treatment with an aqueous solution of potassium iodide and iodine. The cis and trans isomers of **28** and **30** were separated by column chromatography, while attempts to obtain the pure diastereomers of **29** failed. The iodo derivatives **27a** and **27b** were synthesized as previously reported.^16^ The phenyl thioethers **30a** and **30b** were oxidized with *meta*-chloroperbenzoic acid (*m*-CPBA) to give the sulfoxides **31a** and **31b** after 30 min at room temperature (r.t.) with one equivalent of *m*-CPBA or the sulfones **32a** and **32b** after 2 h at r.t. with 2 equivalents of *m*-CPBA. Concerning the sulfoxide derivatives **31a** and **31b**, a further center of chirality was introduced into the molecule. In both cases, only one of the two diastereomers was obtained. The amination of the intermediate iodo derivatives **27–32** with dimethylamine afforded the corresponding free amines **33–38**, which were transformed into the methiodides **3–8** by treatment with methyl iodide.

**Figure 3:**
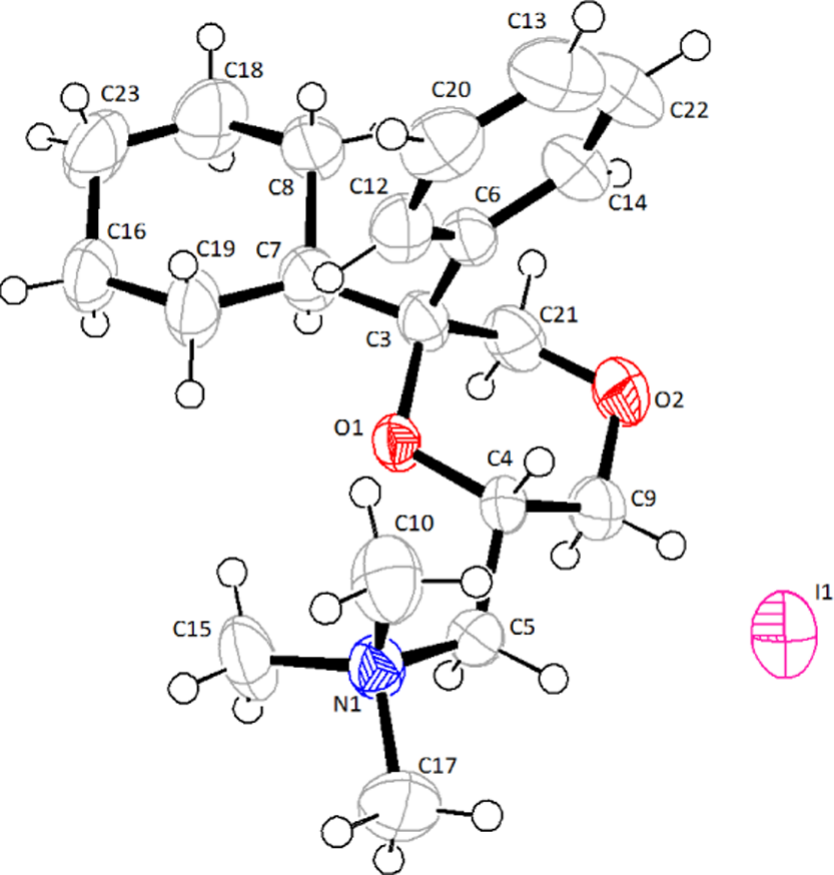
X-ray crystal structure of **3b**. The X-ray coordinates were deposited at Cambridge Crystallographic Data Centre (accession number CCDC 1969353).

The relative configuration between the substituents in 2- and 6-positions of diastereomers **3a** and **3b** was determined by X-ray diffraction analysis performed on **3b** (Figure 3), which confirmed the structure of intermediates **27a** and **27b** previously assigned by ^1^H NMR studies.^16^

**Figure 4:**
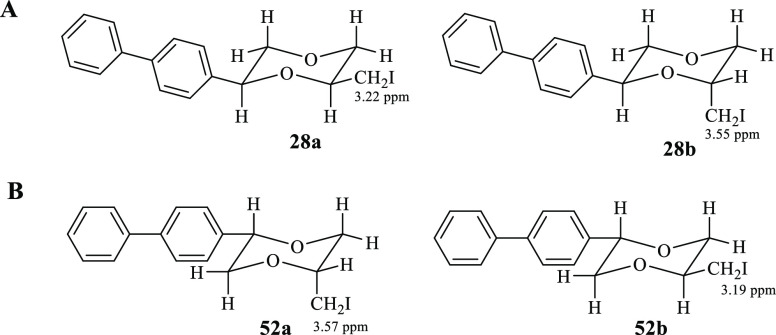
Structure of (A) compounds **28a** and **28b**, precursors of **4a** and **4b**, respectively, and of (B) compounds **52a** and **52b**, precursors of **13a** and **13b**, respectively.

The relative configuration between the substituents in 2- and 6-positions of the 1,4-dioxane ring of diastereoisomers **4a/b** was assigned based on the ^1^H NMR spectra of intermediates **28a/b** (Figure 4A). Because of the steric bulk, one may suppose that both the substituents in 2- and 6-positions of the cis isomers are equatorially oriented, whereas in the trans isomers, only one of the two substituents adopts the equatorial position and the other substituent is axially oriented. In the ^1^H NMR spectrum of the iodo derivative **28b**, precursor of the final methiodide **4b**, the protons of the CH_2_I chain (3.55 ppm) are deshielded compared to the same protons of diastereomer **28a** (3.22 ppm), precursor of methiodide **4a**. This deshielding effect for CH_2_I protons of diastereomer **28b** (see Supporting Information, Figure S4) suggests an axial position for the side chain, as already evidenced in 1,4-dioxane analogues bearing a CH_2_I chain^35^ and, consequently, the relationship between the biphenyl substituent and the chain is trans (Figure 4A).

Similar considerations can be made for diastereomers **30a/b**, precursors of the final methiodides **6a/b**. Indeed, the signals for CH_2_I protons of **30a** and **30b** are positioned at 3.18 and 3.52 ppm, respectively, indicating a trans configuration between the substituents in the 1,4-dioxane nucleus of **30b**.

**Scheme 2:**
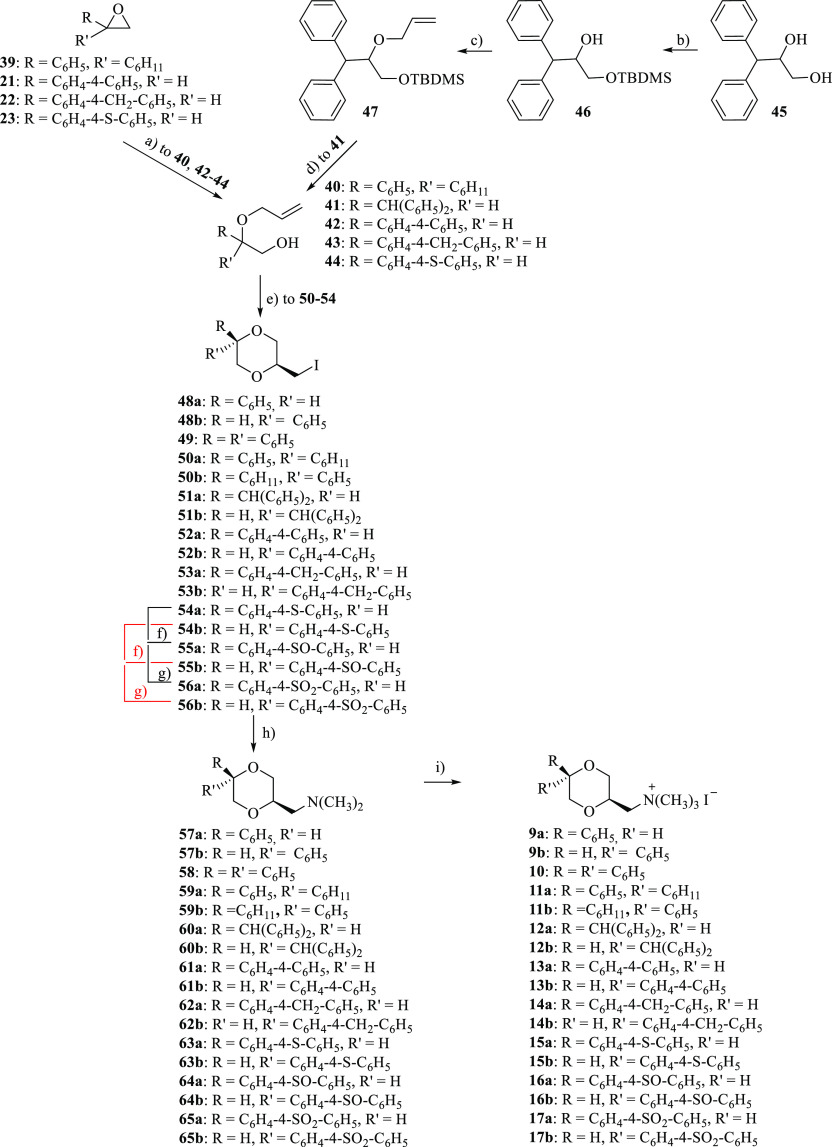
Reagents: (a) HClO_4_, CH_2_=CHCH_2_OH; (b) TBDMSCl, DMAP, (CH_3_CH_2_)_3_N, CH_2_Cl_2_; (c) CH_2_=CHCH_2_Br, NaH, THF. (d) TBAF 1 M in THF, CH_3_COOH; (e) (CH_3_COO)_2_Hg; H_2_O, KI, I_2_; (f) 1 equiv *m*-CPBA, CH_2_Cl_2_, 30 min; (g) 2 equiv *m*-CPBA, CH_2_Cl_2_, 2 h; (h) (CH_3_)_2_NH, C_6_H_6_; (i) CH_3_I, (CH_3_CH_2_)_2_O Only one enantiomer of the racemic mixture is shown.

The novel compounds **9–17** were prepared following the procedure depicted in Scheme 2 and were obtained as racemates. The opening of oxiranes **39**,^36^**21**,^33^**22,** and **23**(34) with allyl alcohol in the presence of perchloric acid yielded compounds **40**, **42**, **43,** and **44**, respectively. The olefine **41** was prepared starting from 3,3-diphenylpropane-1,2-diol (**45**),^37^ whose primary hydroxyl group was selectively protected with *tert*-butyldimethylsilyl chloride (TBDMSCl) to give compound **46**, which was treated with allyl bromide in the presence of NaH affording olefine **47**. The cleavage of the silyl ether with tetrabutylammonium fluoride (TBAF) yielded the corresponding primary alcohol **41**. The intermediates **48** and **49** were obtained as previously described in the literature.^13^

The mixtures of diastereomers **50–54** were obtained starting from olefins **40–44** in the same reaction conditions used for the preparation of **28–30**. The diastereomers were separated by column chromatography. The thioethers **54a** and **54b** were oxidized to give sulfoxides **55a** and **55b**, respectively, and sulfones **56a** and **56b** as above described for **31** and **32**. Similarly to what was observed for **31a** and **31b**, also for **55a** and **55b**, only one of the two diastereomers was obtained. The amination of **48–56** with dimethylamine afforded the corresponding amines **57–65**, which were transformed into the methiodides **9–17** by treatment with methyl iodide (Scheme 2).

The cis and trans configurations between the substituents in 2- and 5-positions of diastereoisomers **9a** and **9b**, respectively, were assigned based on the previously published structures of compounds **48a** and **48b**.^13^ The structures of diastereoisomers **11–15** were assigned by ^1^H NMR spectroscopy. Because of the steric bulk, it may be supposed that in the trans isomers both the substituents in 2- and 5-positions of the 1,4-dioxane nucleus are in the equatorial position, whereas in the cis isomers, only one of the two substituents is equatorially oriented and the other is axially oriented. Analogous to what occurs for the diastereomers **48a** and **48b**,^13^ in the ^1^H NMR spectrum of the iodo derivative **52a**, precursor of the final methiodide **13a**, the protons of the CH_2_I chain (3.57 ppm) are deshielded compared to the same protons of diastereomer **52b** (3.19 ppm), precursor of methiodide **13b** (see Supporting Information, Figure S5). This deshielded effect for CH_2_I protons of diastereomer **52a** suggests an axial position for the side chain, as also evidenced in 1,4-dioxane analogues bearing a 2-CH_2_I chain^35^ and, therefore, a cis configuration between the chain and the biphenyl substituent (Figure 4B).

Similar considerations can be made for diastereomers **51a/b**, **53a/b**, and **54a/b**, precursors of the final methiodides **12a/b**, **14a/b**, and **15a/b**. Indeed, the CH_2_I protons of diastereomers **51a**, **53a**, and **54a** are more deshielded (3.38, 3.58, and 3.52 ppm, respectively) compared to those of diastereomers **51b**, **53b**, and **54b** (3.08, 3.17, and 3.16 ppm, respectively), demonstrating a trans configuration between the substituents in the 1,4-dioxane nucleus for **51a**, **53a**, and **54a**.

**Figure 5:**
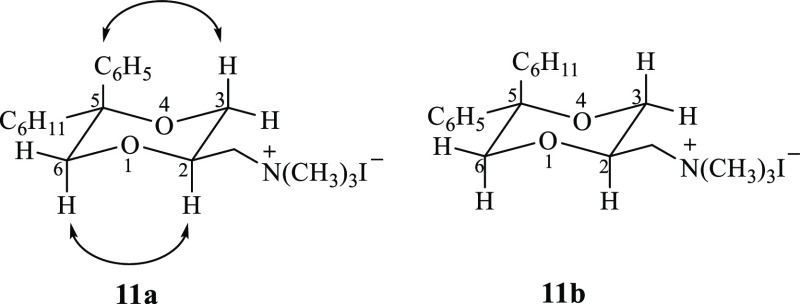
Structure of compounds **11a** and **11b**. The arrows indicate the observed NOEs upon irradiation.

The relative orientation between the CH_2_I fragment and the 5-substituents of **11a** and **11b** was assigned by ^1^H NMR analysis (NOESY studies, see Supporting Information, Figure S7). In particular, evident NOEs were observed between the axial proton in the 3-position and the hydrogen atoms of the phenyl ring in the 5-position and between the axial protons in 2- and 6-positions (4.21 and 3.88 ppm, respectively) of **11a**, indicating that the 5-phenyl nucleus and the 2-side chain are axially and equatorially oriented, respectively. Therefore, the relative configuration between the 2-side chain and the 5-phenyl substituent is cis in **11a** and, consequently, trans in **11b** (Figure 5).

**Scheme 3:**
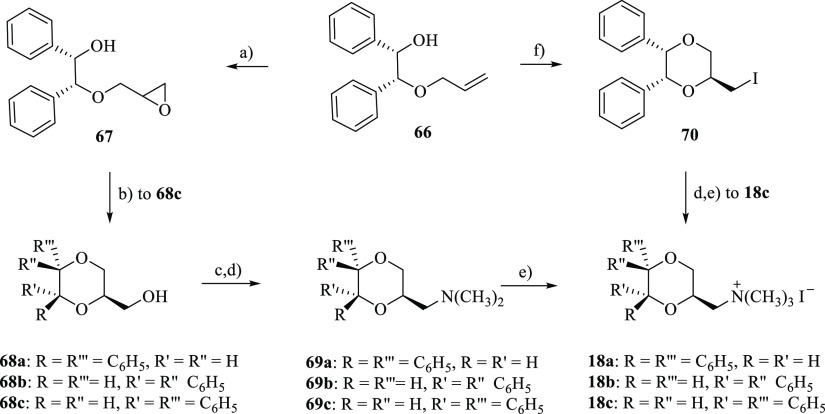
Reagents: (a) *m*-CPBA, CH_2_Cl_2_; (b) CF_3_COOH, CHCl_3_; (c) *p*-TsCl, Pyridine; (d) (CH_3_)_2_NH, C_6_H_6_; (i) CH_3_I, (CH_3_CH_2_)_2_O; (f) (CH_3_COO)_2_Hg; H_2_O, KI, I_2_ Only one enantiomer of the racemic mixture is shown.

Compounds **18a–c** were synthesized following the procedure described in Scheme 3 and were obtained as racemates. The alcohol intermediates **68a** and **68b**, synthesized as previously described,^38^ and **68c**, obtained by treatment of olefine **66**(39) with *m*-CPBA and subsequent reaction of oxirane **67** with trifluoroacetic acid, were reacted with *p*-toluenesulfonyl chloride followed by treatment with dimethylamine to give **69a**, **69b,** and **69c**, whose reaction with methyl iodide afforded the final methiodides **18a**, **18b,** and **18c**, respectively.

**Figure 6:**
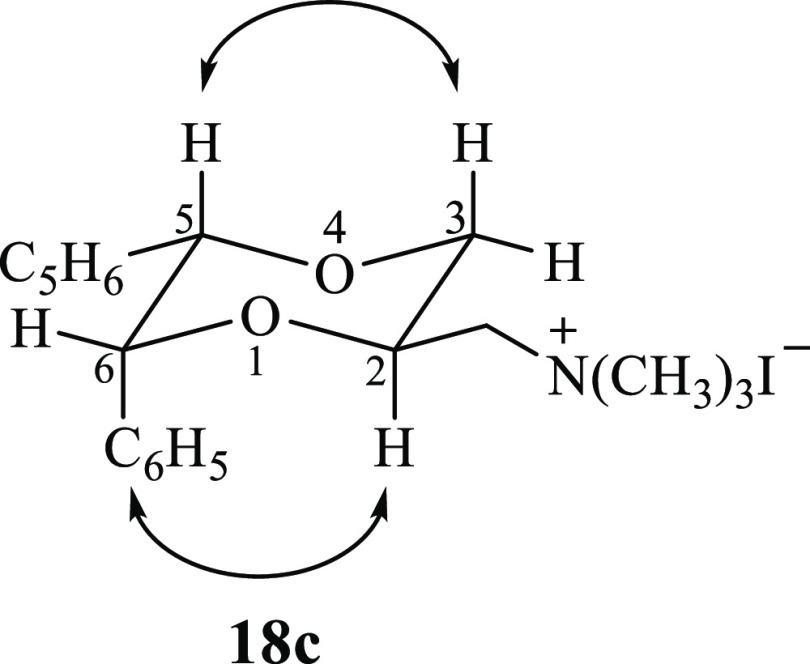
Structure of compound **18c**. The arrows indicate the observed NOEs upon irradiation.

The stereochemical relationship among the substituents of the diastereomers **18a** and **18b** was determined based on the previously assigned structure of the alcohol intermediates **68a** and **68b**.^38^ The stereochemical relationship among the substituents in 2-, 5-, and 6-positions of **18c** was assigned by ^1^H NMR analysis (NOESY studies, see Supporting Information, Figure S8). In the ^1^H NMR spectrum of **18c**, the axial hydrogen atom in the 3-position at δ 3.69 ppm showed two large coupling constants (*J* = 11.2 Hz and *J* = 10.0 Hz), one with the geminal equatorial hydrogen atom and the other with the axial hydrogen atom in the 2-position. Hence, the chain in the 2-position is equatorially orientated. Moreover, NOEs were observed between the axial proton in the 3-position and the proton in the 5-position at 3.69 and 5.22 ppm, respectively, and between the axial proton in the 2-position at 4.24 ppm and the phenyl ring in the 6-position, indicating that the 2-side chain is trans oriented with both phenyl substituents (Figure 6).

In the effort to obtain the fourth diastereomer, in which the stereochemical relationship among the three substituents is cis, the olefine **66**(39) was treated with mercury(II) acetate, followed by an aqueous solution of potassium iodide and iodine. However, also in this case, only one diastereomer (**70**) was obtained. The amination with dimethylamine and subsequent reaction with methyl iodide yielded the same diastereomer (**18c**) obtained following the previously described procedure.

**Scheme 4:**
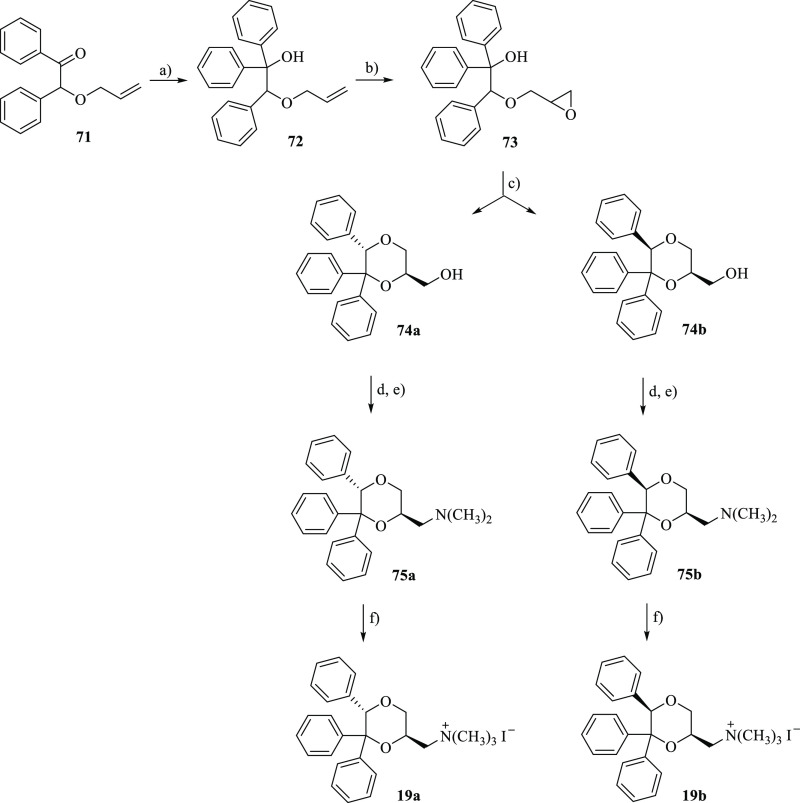
Reagents: (a) C_6_H_5_MgCl, THF/diethyl Ether; (b) *m*-CPBA, CH_2_Cl_2_; (c) CF_3_COOH, CHCl_3_; (d) *p*-TsCl, Pyridine; (e) (CH_3_)_2_NH, C_6_H_6_; (f) CH_3_I, (CH_3_CH_2_)_2_O Only one enantiomer of the racemic mixture is shown.

Compounds **19a** and **19b** were prepared following the procedure described in Scheme 4 and were obtained as racemates. Olefine **72**, obtained by reaction of the α-allyloxy ketone **71**(40) with phenylmagnesium chloride, was treated with *m*-CPBA in CH_2_Cl_2_, affording oxirane **73**, whose treatment with trifluoroacetic acid in CHCl_3_ led to alcohols **74a** and **74b**, which were separated by flash chromatography. Treatment of the alcohols with *p*-toluenesulfonyl chloride followed by reaction with dimethylamine afforded the amines **75a** and **75b**, whose treatment with methyl iodide gave **19a** and **19b**, respectively.

**Figure 7:**
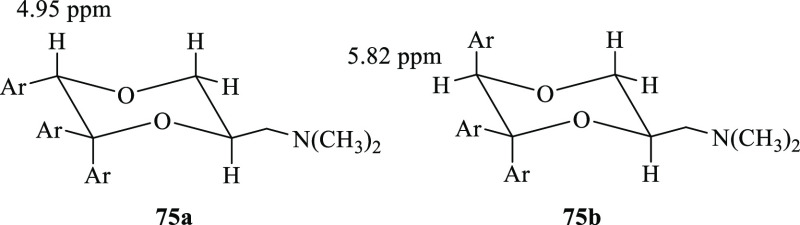
Chemical structures of **19a** and **19b**.

The relative configuration between the 2-substituent and the 5-phenyl group of the diastereomers **19a** and **19b** was assigned by ^1^H NMR spectroscopy. In the ^1^H NMR spectrum of the tertiary amine **75a**, precursor of **19a**, the axial hydrogen atom in the 3-position at 3.78 showed two large coupling constants (*J* = 11.2 Hz and *J* = 10.4 Hz), one with the geminal equatorially located proton and one with the axial proton in the 2-position. Hence, the CH_2_N(CH_3_)_2_ fragment in the 2-position assumes the equatorial position. Analogously, as shown by the ^1^H NMR spectrum of **75b**, precursor of **19b**, the CH_2_N(CH_3_)_2_ fragment in the 2-position is equatorial because the axial proton in the 3-position at 3.58 showed two large coupling constants (*J* = 11.5 Hz and *J* = 10.3 Hz), one with the geminal equatorially positioned hydrogen atom and one with the axially oriented hydrogen atom in the 2-position. Moreover, the proton in the 5-position of **75b** (5.82 ppm) is deshielded compared to the same proton of **75a** (4.95 ppm) (see Supporting Information, Figure S6). The observation that in the ^1^H NMR spectra of the *cis* and *trans* diastereomers of 5-phenyl-1,4-dioxane-2-carboxylic acid and 6-phenyl-1,4-dioxane-2-carboxylic acid, whose structure had previously been determined by NOE measurements,^13^ the equatorially oriented protons are deshielded compared to the axially oriented protons allows us to hypothesize that the proton in the 5-position is axially oriented in **75a** and equatorially oriented in **75b**. Therefore, the relative configuration between the 2- CH_2_N(CH_3_)_2_ chain and the 5-phenyl ring is trans in **19a** and cis in **19b** (Figure 7).

The enantiomers (+)-**3b** and (−)-**3b** were separated by preparative HPLC performed on the intermediate amine (±)-**33b** using a Regis Technologies Whelk-O 1 (*R*,*R*) H (25 cm × 2 cm) column as the chiral stationary phase and *n*-hexane/2-propanol 85/15 v/v as the mobile phase at a flow rate of 18 mL/min. The enantiomeric excess (e.e.), determined by analytical HPLC using a Regis Technologies Whelk-O 1 (*R*,*R*) H (25 cm × 0.46 cm) column as the chiral stationary phase and *n*-hexane/2-propanol 85/15 v/v as the mobile phase at a flow rate of 1 mL/min, proved to be >99.5% for both enantiomers.

**Figure 8:**
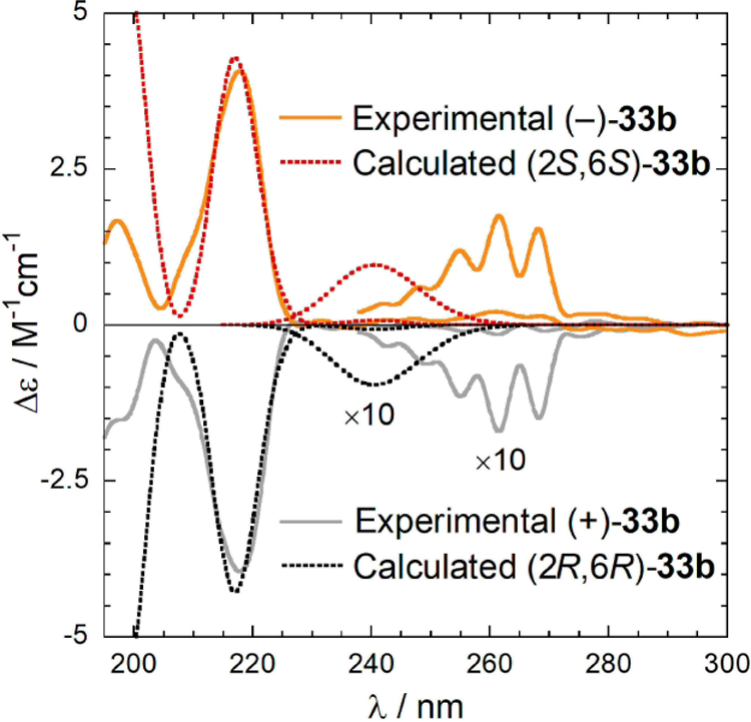
ECD spectra of the oxalate salts of (+)-**33b** and (−)-**33b** measured in acetonitrile. The two regions 190–300 and 235–400 nm were measured with 0.05 and 1 cm cells, respectively.

The absolute configuration of **33b** was determined by quantum mechanical simulations of ECD. The ECD spectra of the two enantiomers of the tertiary amine **33b** (Figure 8), measured on the oxalate salt dissolved in acetonitrile, contain the typical bands of a benzene chromophore attached to a chiral moiety:^41^ the ^1^L_b_ band between 240 and 280 nm, which is electric-dipole forbidden, shows the characteristic vibrational fine structure, and the ^1^L_a_ band between 210 and 225 nm, which is electric-dipole allowed, is more intense.^42,43^

Time-dependent density functional theory (TDDFT) calculations have been shown to be practical means to simulate the CD spectra of this series of ligands, especially with reference to the ^1^L_a_ band.^43^ In fact, the ^1^L_b_ band is more problematic because of some known issues of TDDFT for aromatic hydrocarbons^44^ and because vibronic calculations are needed to reproduce the vibrational pattern.^45^ This fact practically limits the comparison between experiment and calculation to a single band, namely, ^1^L_a_. To exclude possible pitfalls, the present computational protocol based on DFT calculations^46,47^ was first validated on the tertiary amines of (*S*)-**2** and (*R*)-**2**, whose absolute configuration is known.^24^

**Figure 9:**
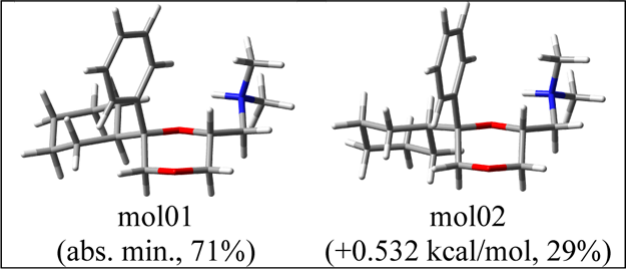
Two populated conformers of the ammonium ion of (2*R*,6*R*)-**33b**.

Conformational analysis and DFT geometry optimizations run on the ammonium ion of (2*R*,6*R*)-**33b** led to only two populated conformers at r.t. (Figure 9). They mainly differ in the rotation around the cyclohexyl-C2 bond, while the rest of the structure is preserved. The preferential orientation of the 2-side chain is dictated by an intramolecular NH–O1 hydrogen bond.

TDDFT calculations were run with several different DFT functionals and basis sets (see ECD and NMR Calculations), either *in vacuo* or including a solvent model for acetonitrile. All explored combinations predicted a negative rotational strength for the ^1^L_a_ band of both conformers, in a very consistent way. Thus, the prediction of the diagnostic ECD band is very robust. In Figure 8, the experimental spectrum is compared with the spectrum calculated at the CAM-B3LYP/def2-SVP/PCM level. As can be seen, the relative energy of the ^1^L_b_ band is overestimated by calculations and the vibrational pattern is missing. Still, the correct negative rotational strength is reproduced for this band too. The agreement between experimental and calculated ECD spectra is satisfactory. Therefore, the absolute configuration is (2*R*,6*R*) for (+)-**33b** and (2*S*,6*S*) for (−)-**33b**.

### Binding Studies

The pharmacological profile of methiodides **3–19** was assessed by radioligand binding assays with human recombinant hM_1_–hM_5_ receptor subtypes stably expressed in Chinese hamster ovary (CHO) cell lines using [^3^H]*N*-methylscopolamine ([^3^H]NMS) as a radioligand to label mAChRs, following previously described protocols.^48,49^ The affinities, expressed as p*K*_i_, are shown in Table 1 along with those of compound **2**, oxybutynin and trospium, which are included for useful comparison.

**Table 1:** Equilibrium Binding Affinity of **2–19**, Oxybutynin, and Trospium

	p*K*_i_^a^				
compd	hM_1_	hM_2_	hM_3_	hM_4_	hM_5_
**2**	9.10^b^	8.24^b^	8.44^b^	8.58^b^	8.36^b^
**3a**	8.03 ± 0.09	7.36 ± 0.12	7.84 ± 0.13	7.51 ± 0.08	7.26 ± 0.05
**3b**	9.28 ± 0.19	7.91 ± 0.13	9.07 ± 0.03	9.03 ± 0.16	8.41 ± 0.1
**4a**	<5	5.06 ± 0.02	<5	<5	<5
**4b**	<5	5.27 ± 0.12	<5	<5	<5
**5a/b**	<5	5.31 ± 0.05	<5	<5	5.13 ± 0.10
**6a**	5.50 ± 0.05	5.47 ± 0.07	<5	5.43 ± 0.05	5.58 ± 0.13
**6b**	5.51 ± 0.01	6.71 ± 0.01	5.77 ± 0.05	5.70 ± 0.02	5.95 ± 0.13
**7a**	<5	5.52 ± 0.02	<5	<5	5.21 ± 0.08
**7b**	5.17 ± 0.08	6.38 ± 0.10	5.88 ± 0.14	5.42 ± 0.02	5.74 ± 0.16
**8a**	5.08 ± 0.09	5.13 ± 0.01	<5	<5	5.21 ± 0.14
**8b**	5.29 ± 0.07	6.70 ± 0.01	5.63 ± 0.14	5.20 ± 0.07	5.80 ± 0.06
**9a**	5.63 ± 0.07	5.06 ± 0.10	5.14 ± 0.10	5.10 ± 0.07	<5
**9b**	<5	<5	<5	<5	<5
**10**	7.44 ± 0.12	6.75 ± 0.09	6.87 ± 0.04	6.81 ± 0.06	6.80 ± 0.06
**11a**	8.01 ± 0.06	7.26 ± 0.08	7.43 ± 0.09	7.15 ± 0.09	7.45 ± 0.08
**11b**	7.81 ± 0.04	7.22 ± 0.11	7.36 ± 0.10	7.23 ± 0.10	7.32 ± 0.09
**12a**	6.28 ± 0.08	5.87 ± 0.09	5.77 ± 0.03	5.60 ± 0.06	5.49 ± 0.06
**12b**	6.08 ± 0.07	6.06 ± 0.08	5.62 ± 0.11	5.52 ± 0.06	5.20 ± 0.01
**13a**	<5	<5	<5	<5	<5
**13b**	<5	5.49 ± 0.02	<5	<5	<5
**14a**	<5	5.65 ± 0.05	5.65 ± 0.12	<5	5.53 ± 0.13
**14b**	<5	<5	<5	<5	<5
**15a**	5.43 ± 0.09	6.70 ± 0.13	6.01 ± 0.11	5.70 ± 0.01	6.00 ± 0.14
**15b**	5.57 ± 0.07	5.86 ± 0.04	5.35 ± 0.06	5.14 ± 0.01	5.40 ± 0.15
**16a**	<5	5.52 ± 0.02	<5	<5	5.21 ± 0.08
**16b**	5.17 ± 0.09	6.38 ± 0.10	5.88 ± 0.14	5.42 ± 0.02	5.72 ± 0.15
**17a**	<5	6.39 ± 0.04	5.14 ± 0.10	<5	5.05 ± 0.08
**17b**	<5	<5	<5	<5	<5
**18a**	5.85 ± 0.09	5.72 ± 0.09	5.33 ± 0.12	5.25 ± 0.08	5.34 ± 0.07
**18b**	6.30 ± 0.09	5.65 ± 0.09	5.81 ± 0.10	5.40 ± 0.09	5.52 ± 0.08
**18c**	7.19 ± 0.07	6.62 ± 0.08	6.45 ± 0.01	6.40 ± 0.06	6.21 ± 0.01
**19a**	6.17 ± 0.16	5.36 ± 0.08	5.70 ± 0.11	5.41 ± 0.01	5.40 ± 0.19
**19b**	6.26 ± 0.19	5.67 ± 0.04	5.96 ± 0.12	5.93 ± 0.03	5.84 ± 0.23
oxybutynin^b^	8.62	7.93	8.82	8.44	7.85
trospium^c^	8.46	8.94	8.99	8.84	8.22

a Inhibition binding constants (p*K*_*i*_) for hM_1_–hM_5_ mAChRs expressed in CHO-K1 cell membranes. The values represent the arithmetic mean ± S.E.M. of at least three independent experiments, each one performed in duplicate.

b Taken from ref (24).

c Taken from ref (50).

The analysis of data reveals that among all the modifications, the replacement of one of the two phenyl rings of **2** with a cyclohexyl group, affording **3**, proved to be the most favorable for the interaction with mAChRs. In particular, the diastereomer **3b**, with a cis configuration between the CH_2_N^+^(CH_3_)_3_ chain in the 2-position and the cyclohexyl fragment in the 6-position of the 1,4-dioxane ring, shows p*K*_i_ values for all mAChR subtypes, except for M_2_, higher than those of the 6,6-diphenyl derivative **2**. Compound **3b** displays a selectivity profile analogous to that of the clinically approved drug oxybutynin, with affinities for M_1_, M_3_, and M_4_ higher than those for M_2_ and M_5_ subtypes. Interestingly, the M_3_/M_2_ selectivity ratio of **3b** (14.5) is significantly higher than those of the lead **2** and trospium (1.6 and 1.1, respectively). The M_3_/M_2_ selectivity profile of **3b** is noteworthy because the presence of a quaternary ammonium head, enhancing the charge transfer interactions that it elicits with the surrounding aromatic residues, generally increases the p*K*_i_ values for all muscarinic subtypes at the expense of the selectivity ratios. Indeed, these aromatic side chains, and in particular four tyrosine residues, represent a structural signature which is completely conserved by all mAChR subtypes.

The trans configuration between the substituents in 2- and 6-positions of the diastereomer **3a** is detrimental for the binding affinity for all the mAChR subtypes, confirming that stereochemistry plays a crucial role in the interaction of 1,4-dioxane derivatives with the mAChRs.^22,24,30^

The replacement of the 6,6-diphenyl group of **2** with a *para*-biphenyl group, affording the diastereomers **4a** and **4b**, induces a dramatic decrease in affinity for all the mAChR subtypes. The higher flexibility of the terminal phenyl group of **4** obtained by introducing a methylene button (mixture **5a/b**) or a sulfur atom (diastereomers **6a** and **6b**) between the two phenyl groups does not improve mAChR affinity. Similar results are obtained by oxidizing the sulfur atom of **6** to sulfoxide and sulfone, affording compounds **7** and **8**, respectively. In the pairs of diastereomers **6a/6b**, **7a/7b**, and **8a/8b**, the trans isomers show p*K*_i_ values slightly higher than those of the corresponding cis isomers.

The shift of the diphenyl group from the 6- to 5-position of the 1,4-dioxane ring of **2**, affording compound **10**, is also detrimental for the binding to the five mAChR subtypes. The removal of one aromatic group of **10**, obtaining the diastereomers **9a** and **9b**, further decreases the mAChR affinity. Similar to what was observed for the 6-substituted ligands, the replacement of an aromatic group of **10** with a cyclohexyl ring is favorable for the binding to the five mAChRs. In this case, stereochemistry seems not to play a role in the binding at mAChRs, both diastereomers **11a** and **11b** showing similar p*K*_i_ values, with a preference for the M_1_ subtype. The increased distance between the diphenyl lipophilic moiety and the ammonium head of **10**, yielding the diastereomers **12a** and **12b**, decreases the p*K*_i_ values for all the mAChRs. Analogous to what was observed for the corresponding 6-substituted derivatives, all the other modifications performed on the 6,6-diphenyl group of **10** (*i.e.*, its replacement with C_6_H_4_-4-C_6_H_5_, C_6_H_4_-4-CH_2_-C_6_H_5_, C_6_H_4_-4-S-C_6_H_5_, C_6_H_4_-4-SO-C_6_H_5_, and C_6_H_4_-4-SO_2_-C_6_H_5_), affording **13–17**, are detrimental for the affinity for mAChRs. Though with low affinity, the diphenylsulfone **17a** shows selectivity for M_2_ over the other subtypes. This selectivity profile agrees with what was reported for other muscarinic derivatives bearing the diphenylsulfone moiety.^51^

Compared to the 5-mono-phenyl derivatives **9a** and **9b** and the previously described 6-mono-phenyl derivatives,^24^ the presence of a phenyl substituent in both 5- and 6-positions of the 1,4-dioxane ring (**18a**, **18b** and **18c**) seems to be advantageous, especially when the two phenyl groups are in a cis stereochemical relationship (**18c**). Instead, the insertion of a phenyl substituent in the 5-position of the 6,6-diphenyl derivative **2**, affording **19a** and **19b**, markedly reduces the binding affinities.

The well-established influence of chirality on the biological activity of mAChR ligands^22,24,30^ prompted us to prepare and study the enantiomers of the most interesting ligand **3b**. Moreover, considering that the basic function of mAChR antagonists can also be a tertiary amine,^24^ the racemic **33b** and its enantiomers were included in this study.

The p*K*_i_ values of (±)-**3b**, (±)-**33b** and their enantiomers (2*R*,6*R*)-(+)-**3b** and (2*S*,6*S*)-(−)-**3b**, (2*R*,6*R*)-(+)-**33b** e (2*S*,6*S*)-(−)-**33b** are reported in Table 2 together with those of the lead compound (±)-**2** and its enantiomers (*R*)-(+)-**2** and (*S*)-(−)-**2**.

**Table 2:** Equilibrium Binding Affinity of (±)-**2**, (±)-**3b**, (±)-**33b**, and Their Enantiomers

	p*K*_i_^a^				
Compd	hM_1_	hM_2_	hM_3_	hM_4_	hM_5_
(±)-**2**	9.10^b^	8.24^b^	8.44^b^	8.58^b^	8.36^b^
(*R*)-(+)-**2**	7.79^b^	7.48^b^	7.21^b^	6.82^b^	6.97^b^
(*S*)-(−)-**2**	9.30^b^	8.55^b^	8.83^b^	8.83^b^	8.77^b^
ER	32	12	42	102	63
(±)-**3b**	9.28 ± 0.22	7.91 ± 0.13	9.07 ± 0.03	9.03 ± 0.16	8.41 ± 0.36
(2*R*,6*R*)-(+)-**3b**	8.30 ± 0.25	7.86 ± 0.14	7.51 ± 0.12	7.45 ± 0.11	7.68 ± 0.35
(2*S*,6*S*)-(−)-**3b**	9.52 ± 0.19	8.22 ± 0.10	9.05 ± 0.10	9.17 ± 0.19	9.18 ± 0.18
ER	17	2	35	52	24
(±)-**33b**	8.86 ± 0.16	7.88 ± 0.08	8.72 ± 0.10	8.62 ± 0.12	8.62 ± 0.17
(2*R*,6*R*)-(+)-**33b**	7.68 ± 0.16	7.17 ± 0.11	6.73 ± 0.06	7.01 ± 0.08	7.16 ± 0.27
(2*S*,6*S*)-(−)-**33b**	9.10 ± 0.28	8.10 ± 0.10	9.02 ± 0.10	9.09 ± 0.15	8.83 ± 0.10
ER	26	9	195	120	47

a See footnote a in the legend of Table 1.

b Taken from ref (24).

As expected, the data reveal how the racemic tertiary amine (±)-**33b** shows high affinity for all mAChRs, though with p*K*_i_ values slightly lower than those of the corresponding ammonium salt (±)-**3b**. Moreover, it maintains the interesting selectivity for M_3_ over M_2_ subtype (M_3_/M_2_ = 7.0) already observed with methiodide (±)-**3b** (M_3_/M_2_ = 14.5). Between the enantiomers of the tertiary amine [(2*R*,6*R*)-(+)-**33b** and (2*S*,6*S*)-(−)-**33b**] as well as those of the quaternary ammonium salt [(2*R*,6*R*)-(+)-**3b** and (2*S*,6*S*)-(−)-**3b**], the eutomers are the ones in which the absolute configuration of the carbon atom in position 2 is *S* [(2*S*,6*S*)-(−)-**33b** and (2*S*,6*S*)-(−)-**3b**, respectively]. Such a configuration is the same of the eutomer (*S*)-(−)-**2**, suggesting that these derivatives bind to the same mAChR sites. The eudismic ratios (ERs) between the enantiomers of the tertiary amine are significantly higher than those between the corresponding enantiomers of the methiodide for all mAChR subtypes, especially for M_3_, for which the eutomer (2*S*,6*S*)-(−)-**33b** shows a p*K*_i_ value 195-fold higher than that of the distomer (2*R*,6*R*)-(+)-**33b**.

### Docking Studies

**Figure 10:**
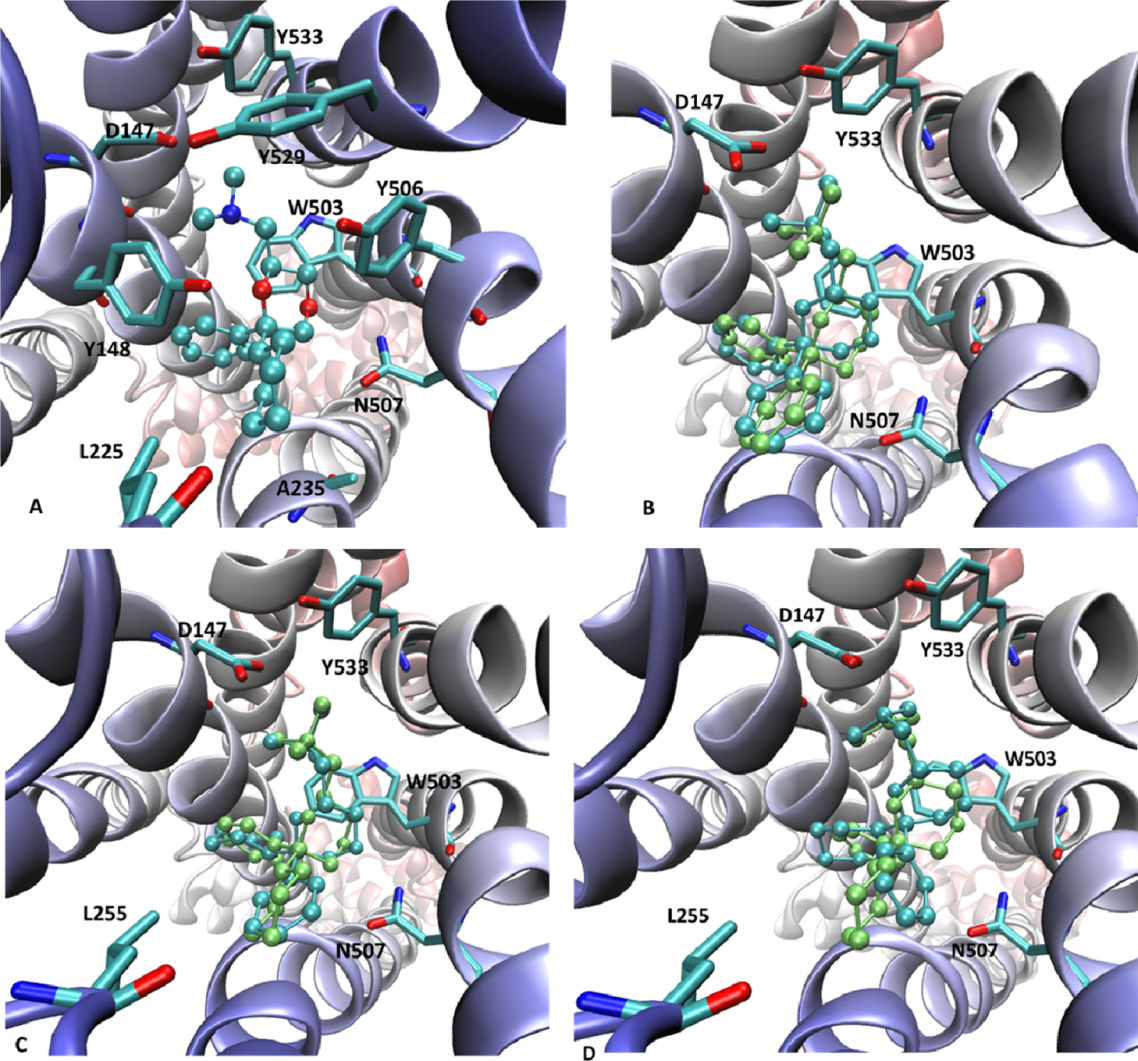
Main interactions stabilizing the putative complex for (*2S*,*6S*)-**3b** with the M_3_ mAChR structure (PDB Id: 5ZHP) (A). Comparison between the complexes for the two enantiomers of **2** (B), **3b** (C), and **33b** (D). In all comparisons, the eutomer is in lime and the distomer in azure.

To investigate the factors influencing the observed enantioselectivity of the three pairs of enantiomers (*R*)-**2**/(*S*)-**2**, (2*R*,6*R*)-**3b**/(2*S*,6*S*)-**3b**, and (2*R*,6*R*)-**33b**/(2*S*,6*S*)-**33b**, docking simulations were carried out on the human M_3_ mAChR structure in complex with a selective antagonist (PDB Id: 5ZHP).^52^Figure 10A, showing the putative complex as computed for compound (*2S*,*6S*)-**3b**, endowed with the highest affinity, reveals the following set of interactions: (a) the charged ammonium head is engaged by a set of contacts comprising the key ion-pair with Asp147^3.32^ plus several charge transfer interactions with surrounding aromatic side chains (*e.g.*, Tyr148^3.33^, Trp503^6.48^, Tyr506^6.51^, Tyr529^7.39^, and Tyr533^7.43^); (b) the O4 dioxane atom is involved in a key H-bond with Asn507^6.52^, while the O1 atom is shielded by the close ammonium head and cannot elicit significant interactions; (c) the phenyl ring can stabilize π–π stacking interactions with a set of surrounding aromatic residues such as Tyr148^3.33^, Trp199^4.57^ and Trp503^6.48^; (d) the cyclohexyl ring is accommodated within a subpocket in which it can contact alkyl side chains such as Leu225^ECL2^, Ala235^5.43^, and Ala238^5.46^. On these grounds, one may argue that the observed enantioselectivity can be ascribed to four moieties, the arrangement of which is influenced by the chiral centers: (a) the O4 dioxane atom, a feature which involves all three pairs of enantiomers; (b) the cyclohexyl and (c) the phenyl rings which concern only the compounds **3b** and **33b**; (d) the ammonium head which seems to play a marginal role for **2** and **3** reasonably due to the symmetry of the trimethyl ammonium group, while the need to properly arrange the proton toward Asp147^3.32^, and the *N*-methyl groups toward the aromatic residues, may impact on the enantioselectivity of **33b**.

Hence, inspection of Figure 10B, comparing the computed poses for the two enantiomers of compound **2**, reveals that they suitably and similarly arrange the phenyl rings and the ammonium head, while the pose of the dioxane ring differs in the two complexes. In detail, Figure 10B shows that the eutomer (*S*)-**2** is able to establish a strong H-bond with Asn507^6.52^, while the distomer (*R*)-**2** less suitably arranges the O4 atom (as defined by both N–H···O distance, 2.1 Å *vs* 2.7 Å, and corresponding angle, 171.5 *vs* 103.6, for (*S*)-**2** and (*R*)-**2**, respectively) which, therefore, weakly contacts Asn507^6.52^.

Similarly, Figure 10C, comparing the best obtained complexes for the two enantiomers of compound **3b**, shows that both of them are able to conveniently accommodate the dioxane ring (*e.g.*, the N–H···O4 distance with Asn507^6.52^ is equal to 2.3 Å in both complexes) and the ammonium head but unavoidably differ for the arrangements of the two rings in the 6-position. Indeed, while the eutomer (*2S*,6*S*)-**3b** properly accommodates the phenyl and the cyclohexyl rings as described above, the distomer (*2R*,6*R*)-**3b** is constrained to approach the phenyl ring toward the alkyl side chains with the cyclohexyl ring completely surrounded by aromatic residues. Notably, the capacity of both enantiomers of **3b** to stabilize similar H-bonds with Asn507^6.52^ suggests that the greater (despite always restricted) flexibility of the cyclohexyl ring with respect to the phenyl one allows the distomer (2*R*,6*R*)-**3b** to minimize the configurational effects on the pose of the dioxane ring.

Finally, Figure 10D, comparing the best poses as computed for the two enantiomers of **33b**, highlights that they differ for the arrangement of both the O4 dioxane atom and the cyclohexyl/phenyl rings. In detail, while the eutomer (*2S*,6*S*)-**33b** can elicit the key H-bond with Asn507^6.52^ (N–H···O4 distance with Asn507^6.52^ is equal to 2.6 Å) and to insert the cyclohexyl and phenyl rings within the suitable subpockets, the distomer (*2R*,6*R*)-**33b** cannot contact Asn507^6.52^ (N–H···O4 distance with Asn507^6.52^ is equal to 3.8 Å) and accommodates the two rings in the 6-position within the wrong subpockets. Notably, the unique difference between **3b** and **33b** involves the ammonium head which is a quaternary salt only in the former. Figure 10D indicates that both enantiomers of **33b** are able to properly arrange the ammonium head even though the lack of the symmetric trimethyl group in **33b** increases the relevance of the C2 configuration and can explain why the enantiomers of **33b** are constrained to differ for the arrangement of the O4 dioxane atom, while both enantiomers of **3b** are able to properly accommodate the dioxane ring by minimizing the effects of the C2 configuration.

These observations find encouraging confirmations in the reported ERs, thus allowing for some meaningful considerations. First, the observed differences in the dioxane arrangement exert a conceivably greater impact on affinity compared to those in the cyclohexyl/phenyl rings as seen when comparing the ER values of **2** and **3b**. Again, the combination of both factors (dioxane and cyclohexyl/phenyl rings) reveals a synergistic effect by showing an ER value for **33b** markedly higher than the previous ones. Such a synergistic effect can be explained at an atomic level by considering that, while both enantiomers of **2** are able to stabilize the H-bond with Asn507^6.52^ even though the distomer elicits weaker interactions (as seen in the reported geometrical parameters), the **33b** distomer is substantially unable to approach Asn507^6.52^, thus missing this key interaction. Finally, similar trends can also be seen when analyzing the corresponding affinity values and, in particular, the affinities of the distomers. Indeed, while the eutomers show comparable affinity values with **3b** and **33b** which reveal slightly higher values probably due to the favorable hydrophobic interaction stabilized by the cyclohexyl ring, the distomers show greater differences in affinity which are ascribable to their reduced interactions. Hence, (2*R*,6*R*)-**3b** which only fails in properly arranging the rings in the 6-position reveals the greatest affinity, followed by (*R*)-**2** which elicits a weak H-bond with Asn507^6.52^. The lowest affinity is shown by (2*R*,6*R*)-**33b**, which does not stabilize the mentioned H-bond and unsuitably arranges the rings in the 6-position.

For completeness and even though the affinity values of the single enantiomers were not measured, docking simulations also involved other proposed derivatives by focusing attention on those with p*K*_i_ values on M_3_ mAChR greater than 6. While avoiding systematic analyses, the docking results allow for some general considerations. The lower affinity values of the ligands bearing cyclohexyl/phenyl rings in 5 (instead of 6, *e.g.*, **10**, **11a**, and **11b**) can be ascribed to the steric hindrance exerted by these rings on the O4 dioxane atom which weakens the key H-bond with Asn507^6.52^. In contrast, the reduced steric hindrance exerted on the O1 dioxane atom allows this to be engaged in additional H-bonds as seen for (2*S*,6*S*)-**11a** with Tyr148^3.33^. The low affinity of ligands bearing a 4-(phenylthio)phenyl moiety (**15a** and **15b**) and similar diphenyl groups is explainable by considering that these bulky substituents constrain the ligands to assume inconvenient poses, where even the ammonium head assumes suboptimal arrangements, without adding any additional contacts. Finally, the lower affinity values of the ligands with substituents in both 5- and 6-positions (*e.g.*, **18c**) is ascribable to the same factors affecting the binding of compounds substituted only in 5, namely, the greater steric hindrance on the O4 dioxane atom which weakens the H-bond with Asn507^6.52^.

### Functional Studies on MSCs from Mouse Bone Marrow

**Figure 11:**
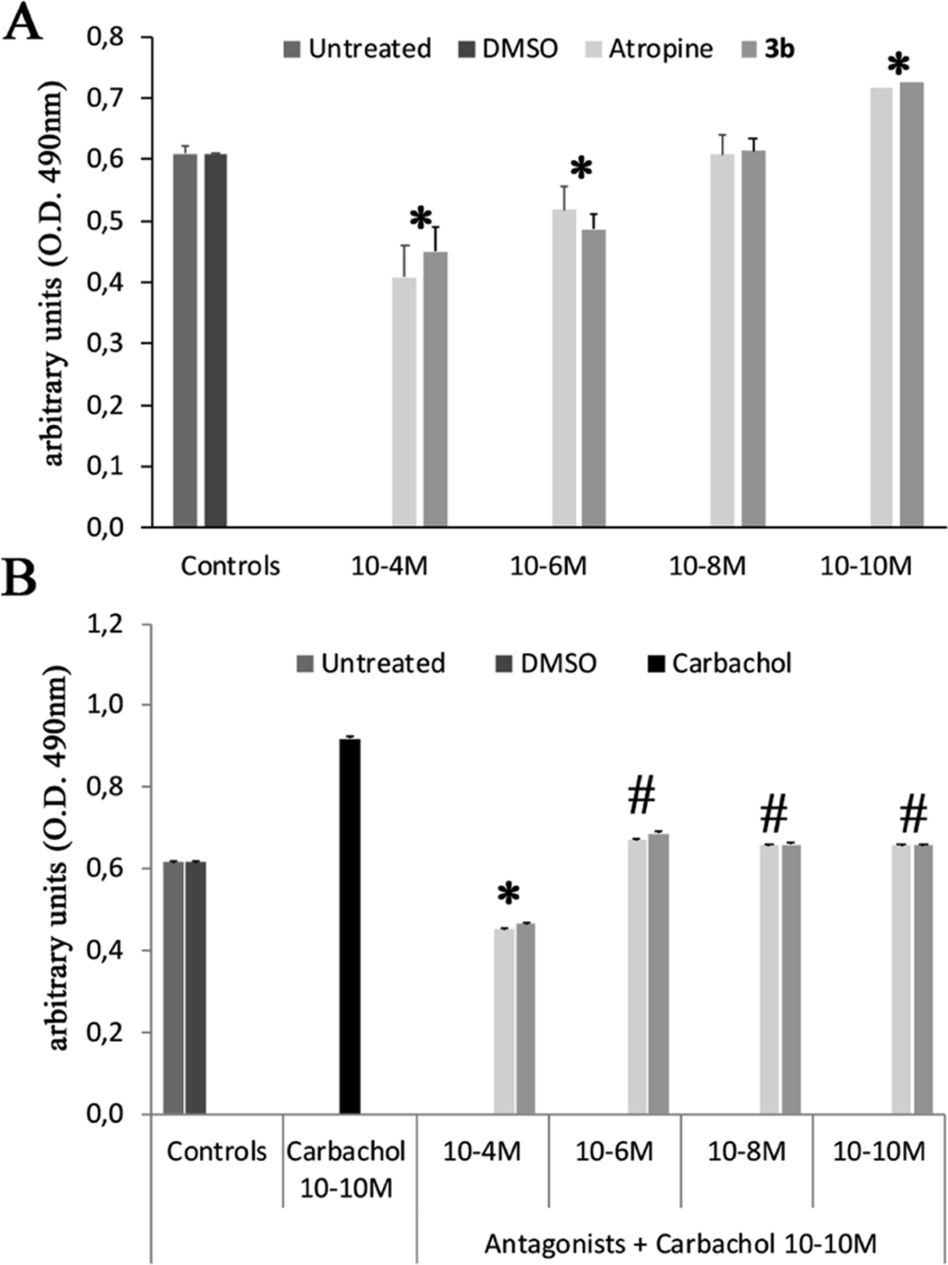
(A) Dose–response effect of **3b** and atropine on the metabolic activity of viable MSCs. The graphic represents the mean ± SEM of four independent experiments; *p < 0.05 *vs* controls (Untreated MSCs and DMSO). (B) Effects of carbachol (10^–10^ M) on the metabolic activity of MSCs in the absence or in the presence of different doses of **3b** or atropine. The graphic represents the mean ± SEM of four independent experiments; *p < 0.05 *vs* controls; #*p* < 0.05 *vs* carbachol.

It is well established that bone marrow MSC behavior is influenced by a variety of signaling systems. In context, cholinergic intramural stimuli and, in particular, muscarinic signals orchestrate MSCs viability and commitment.^11,12^ Considering also the pluripotent MSC nature and their contribution to bone, blood, and systemic homeostasis,^53^ viability studies on MSCs were performed to determine the functional profile of **3b**, the most interesting compound in this series. Namely, the effect of this compound was similar to that of the well-known mAChR antagonist atropine because it was able to down-regulate MSCs viability when used at high concentration (10^–4^ M), while increased cell viability when used at low concentration (10^–10^ M) (Figure 11A). Successively, the efficacy of **3b** in contrasting cell viability induced by the well-known mAChR agonist carbachol was evaluated. The data reported in Figure 11B indicate that, analogous to atropine, the new compound **3b** is able to contrast the increase of carbachol-induced MSC viability, confirming its mAChR antagonist profile. Further research on the intracellular mechanistic outcomes of **3b** on MSCs remains mandatory.

## Conclusions

In the present study, the 6,6-diphenyl structural element of the potent mAChR antagonist **2** was replaced by lipophilic substituents in 5- and/or 6-position of the 1,4-dioxane nucleus. Among the novel compounds, the 6-cyclohexyl-6-phenyl derivative **3b**, with a cis configuration between the CH_2_N^+^(CH_3_)_3_ chain in the 2-position and the cyclohexyl ring in the 6-position, showed p*K*_i_ values for all mAChR subtypes, except for M_2_, higher than those of **2**. Moreover, its selectivity profile is similar to that of the therapeutically used drug oxybutynin, with p*K*_i_ values for M_1_, M_3_, and M_4_ subtypes higher than those for M_2_ and M_5_ subtypes. The study of the enantiomers of **3b** and those of the corresponding tertiary amine **33b**, whose absolute configuration was determined by quantum mechanical simulations of ECD, provided useful information about the role played by chirality in the interaction with mAChRs. In particular, the absolute configuration of the carbon atom in the 2-position of the eutomers (2*S*,6*S*)-(−)-**3b** and (2*S*,6*S*)-(−)-**33b** is the same as (*S*)-(−)-**2**, suggesting that these derivatives bind to the same mAChR sites. The ERs between the enantiomers of the tertiary amine **33b** proved to be higher than those between the corresponding enantiomers of methiodide **3b** for all mAChR subtypes, especially for M_3_. Docking studies on the M_3_ mAChR-resolved structure allowed us to shed light on the binding mode of the proposed compounds. In particular, while the enantiomers of **33b** differ for the arrangement of O4 dioxane atom, both enantiomers of **3b** are able to properly accommodate the dioxane ring by minimizing the effect of the C2 configuration. Finally, the assays on MSCs from mouse bone marrow showed for **3b** a functional profile similar to that of the mAChR antagonist atropine concerning both the dose–response effect produced on the metabolic activity of viable MSCs and the effect in contrasting the increase of carbachol-induced MSC viability.

Compared to the tertiary amine drugs clinically used for the treatment of OAB, **3b** presents a quaternary ammonium function that should prevent the crossing of BBB, minimizing central anticholinergic activity and, therefore, limiting CNS side effects. The prediction by SwissADME that **3b** is a potential P-gp substrate makes the profile of such a compound more and more interesting.^54^ Not to mention that the transformation into a quaternary amine markedly enhances the metabolic stability of this compound. Indeed, the metabolic prediction based on the similarity analysis using the MetaQSAR database on the tertiary amine indicates the oxidation in alpha to the N atom as a truly probable metabolic reaction which is largely inhibited by the presence of a permanent positive charge.^55^ Moreover, the M_3_/M_2_ selectivity ratio of **3b** (14.5), which is significantly higher than those of the quaternary ammonium compounds **2** and trospium (1.6 and 1.1, respectively), might limit cardiovascular side effects. Therefore, the methiodide **3b** might represent a valuable lead compound for the design of novel antagonists potentially useful in peripheral diseases in which M_3_ receptors are involved.

## Experimental Section

### Chemistry

#### General

Melting points (mp) were taken in glass capillary tubes on a Büchi SMP-20 apparatus and are uncorrected. ^1^H NMR and ^13^C NMR spectra were recorded on Varian GEM200, Varian Mercury AS400, or Bruker 500 MHz instruments, and chemical shifts (ppm) are reported relative to tetramethylsilane. Spin multiplicities are given as s (singlet), d (doublet), dd (double doublet), t (triplet), or m (multiplet). IR spectra were recorded on a PerkinElmer 297 instrument, and spectral data (not shown because of the lack of unusual features) were obtained for all compounds reported and are consistent with the assigned structures. The microanalyses were recorded on a FLASH 2000 instrument (Thermo Fisher Scientific). The elemental composition of the compounds agreed to within ±0.4% of the calculated value. Optical activity was measured at 20 °C with a PerkinElmer 241 polarimeter. Analytical chiral HPLC was performed on a Shimadzu chromatography system using a Regis Technologies (*R*,*R*)-Whelk-O 1 (25 cm × 0.46 cm) column. Preparative chiral HPLC was performed on a Shimadzu chromatography system using a Regis Technologies (*R*,*R*)-Whelk-O 1 (25 cm × 2 cm). Mass spectra were obtained using a Hewlett Packard 1100 MSD instrument utilizing electron-spray ionization (ESI). The compounds were detected, and a purity of >95% was confirmed by UV absorption at 220 nm. All reactions were monitored by thin-layer chromatography using silica gel plates (60 F254; Merck), visualizing with ultraviolet light. Chromatographic separations were performed on silica gel columns (Kieselgel 40, 0.040–0.063 mm, Merck) by flash chromatography. Compounds were named following IUPAC rules as applied by ChemBioDraw Ultra (version 11.0) software for systematically naming organic chemicals. The purity of the novel compounds was determined by combustion analysis and was ≥95%.

#### 1-((2R*,6S*)-6-Cyclohexyl-6-phenyl-1,4-dioxan-2-yl)-*N*,*N*,*N*-trimethylmethanaminium Iodide (**3a**)

A solution of **33a** (0.13 g, 0.4 mmol) in Et_2_O (10 mL) was treated with an excess of methyl iodide and left at r.t. in the dark for 24 h. The solid was filtered and recrystallized from EtOH (91% yield); mp 270–271 °C. ^1^H NMR (DMSO): δ 0.42–1.91 (m, 11H, cyclohexyl), 3.02–3.56 (m, 14H, N(CH_3_)_3_, CH_2_N, dioxane), 3.79 (m, 1H, dioxane), 4.62 (d, *J* = 12.1, 1H, dioxane), 7.18–7.40 (m, 5H, ArH). ^13^C NMR (DMSO): δ 26.0, 26.4, 26.6, 27.0, 28.5, 37.5 (cyclohexyl); 54.1 (N(CH_3_)_3_); 63.6, 66.8, 67.9, 70.0, 78.9 (CH_2_N and dioxane); 126.0, 127.4, 127.8 (ArH); 140.5 (Ar). ESI/MS *m*/*z*: 318.2 [M]^+^, 763.4 [2M + I]^+^. Anal. Calcd (C_20_H_32_INO_2_) C, H, N.

#### 1-((2R*,6R*)-6-Cyclohexyl-6-phenyl-1,4-dioxan-2-yl)-*N*,*N*,*N*-trimethylmethanaminium Iodide (**3b**)

This compound was prepared starting from **33b** following the procedure described for **3a**: a white solid was obtained, which was recrystallized from 2-PrOH (87% yield); mp 245–246 °C. ^1^H NMR (DMSO): δ 0.62–1.94 (m, 11H, cyclohexyl), 2.98–3.61 (m, 14H, N(CH_3_)_3_, CH_2_N, dioxane), 3.86 (m, 1H, dioxane), 4.62 (d, *J* = 12.2, 1H, dioxane), 7.21–7.54 (m, 5H, ArH). ^13^C NMR (DMSO): δ 26.5, 27.3, 47.6 (cyclohexyl); 54.2 (N(CH_3_)_3_); 64.9, 65.9, 68.0, 69.2, 80.2 (CH_2_N and dioxane); 127.7, 128.3, 128.7 (ArH); 139.8 (Ar). ESI/MS *m*/*z*: 318.2 [M]^+^, 763.4 [2M + I]^+^. Anal. Calcd (C_20_H_32_INO_2_) C, H, N.

#### 1-((2*S*,6*S*)-6-Cyclohexyl-6-phenyl-1,4-dioxan-2-yl)-*N*,*N*,*N*-trimethylmethanaminium Iodide [(2*S*,6*S*)-(−)-**3b**]

This compound was prepared starting from (2*S*,6*S*)-(−)-**33b** following the procedure described for **3a**: a white solid was obtained, which was recrystallized from 2-PrOH (88% yield). [α]_D_^20^ = −42.5 (c 1, CH_3_OH); mp and ^1^H NMR spectrum were identical to those of racemic compound (±)-**3b**. Anal. Calcd (C_20_H_32_INO_2_) C, H, N. C, 53.94; H, 7.24; N, 3.14. Found: C, 54.06; H, 7.41; N, 3.29.

#### 1-((2*R*,6*R*)-6-Cyclohexyl-6-phenyl-1,4-dioxan-2-yl)-*N*,*N*,*N*-trimethylmethanaminium Iodide [(2*R*,6*R*)-(+)-**3b**]

This compound was prepared starting from (2*R*,6*R*)-(+)-**33b** following the procedure described for **3a**: a white solid was obtained, which was recrystallized from 2-PrOH (85% yield). [α]_D_^20^ = +42.9 (c 1, CH_3_OH); mp and ^1^H NMR spectrum were identical to those of racemic compound (±)-**3b**. Anal. Calcd (C_20_H_32_INO_2_) C, H, N.

#### 1-((2R*,6S*)-6-([1,1′-Biphenyl]-4-yl)-1,4-dioxan-2-yl)-*N*,*N*,*N*-trimethylmethanaminium Iodide (**4a**)

This compound was prepared starting from **34a** following the procedure described for **3a**: a white solid was obtained, which was recrystallized from EtOH (88% yield); mp 242–243 °C. ^1^H NMR (DMSO): δ 3.03–3.58 (m, 13H, CH_2_N, N(CH_3_)_3_, dioxane), 3.76 (dd, *J* = 11.4, 2.4 Hz, 1H, dioxane), 3.95 (dd, *J* = 11.4, 2.8 Hz, 1H, dioxane), 4.45 (m, 1H, dioxane), 4.89 (dd, *J* = 10.3, 2.6 Hz, 1H, dioxane), 7.24–7.75 (m, 9H, ArH). ESI/MS *m*/*z*: 312.2 [M]^+^, 751.3 [2M + I]^+^. Anal. Calcd (C_20_H_26_INO_2_) C, H, N.

#### 1-((2R*,6R*)-6-([1,1′-Biphenyl]-4-yl)-1,4-dioxan-2-yl)-*N*,*N*,*N*-trimethylmethanaminium Iodide (**4b**)

This compound was prepared starting from **34b** following the procedure described for **3a**: a white solid was obtained, which was recrystallized from MeOH (89% yield); mp 256–257 °C. ^1^H NMR (DMSO): δ 3.15 (s, 9H, N(CH_3_)_3_), 3.43–3.95 (m, 5H, CH_2_N and dioxane), 4.30 (dd, *J* = 13.7, 10.1 Hz, 1H, dioxane), 4.51 (m, 1H, dioxane), 5.16 (dd, *J* = 9.5, 2.9 Hz, 1H, dioxane), 7.32–7.73 (m, 9H, ArH). ESI/MS *m*/*z*: 312.2 [M]^+^, 751.3 [2M + I]^+^. Anal. Calcd (C_20_H_26_INO_2_) C, H, N.

#### 1-((2R*,6S*)-6-(4-Benzylphenyl)-1,4-dioxan-2-yl)-*N*,*N*,*N*-trimethylmethanaminium Iodide and 1-((2R*,6R*)-6-(4-Benzylphenyl)-1,4-dioxan-2-yl)-*N*,*N*,*N*-trimethylmethanaminium Iodide (**5a/b**)

This mixture of cis/trans (6:4) diastereomers was prepared starting from **35a/b** following the procedure described for **3a**: a white solid was obtained, which was recrystallized from MeOH (89% yield); mp 228–232 °C. ^1^H NMR (DMSO): δ 2.94–3.98 (s, 17H cis + 17H trans, N(CH_3_)_3_, CH_2_N, CH_2_Ar and dioxane), 4.19–4.53 (m, 1H cis + 1H trans, dioxane), 4.79 (dd, 1H *cis*, *J* = 13.7, 10.1 Hz, 1H, dioxane), 5.06 (dd, 1H trans, *J* = 13.7, 10.1 Hz, 1H, dioxane), 7.08–7.38 (m, 9H cis + 9H trans, ArH). ESI/MS *m*/*z*: 326.2 [M]^+^. Anal. Calcd (C_21_H_28_INO_2_) C, H, N.

#### 1-((2R*,6S*)-6-(4-(Phenylthio)phenyl)-1,4-dioxan-2-yl)-*N*,*N*,*N*-trimethylmethanaminium Iodide (**6a**)

This compound was prepared starting from **36a** following the procedure described for **3a**: a white solid was obtained, which was recrystallized from MeOH (89% yield); mp 196–198 °C. ^1^H NMR (DMSO): δ 3.12 (s, 9H, N(CH_3_)_3_), 3.18–3.52 (m, 4H, CH_2_N, dioxane), 3.71 (m, 1H, dioxane), 3.93 (dd, *J* = 10.1, 3.3 Hz, 1H, dioxane), 4.42 (m, 1H, dioxane), 4.82 (dd, *J* = 10.1, 2.2 Hz, 1H, dioxane), 7.23–7.48 (m, 9H, ArH). ESI/MS *m*/*z*: 344.2 [M]^+^. Anal. Calcd (C_20_H_26_INO_2_S) C, H, N, S.

#### 1-((2R*,6R*)-6-(4-(Phenylthio)phenyl)-1,4-dioxan-2-yl)-*N*,*N*,*N*-trimethylmethanaminium Iodide (**6b**)

This compound was prepared starting from **36b** following the procedure described for **3a**: a white solid was obtained, which was recrystallized from EtOH (89% yield); mp 197–198 °C. ^1^H NMR (DMSO): δ 3.02–3.98 (m, 14, N(CH_3_)_3_, CH_2_N, dioxane), 4.22 (dd, *J* = 9.6, 2.7 Hz, 1H, dioxane), 4.48 (m, 1H, dioxane), 5.11 (dd, *J* = 13.4, 10.0 Hz, 1H, dioxane), 7.21–7.42 (m, 9H, ArH). ESI/MS *m*/*z*: 344.2 [M]^+^. Anal. Calcd (C_20_H_26_INO_2_S) C, H, N, S.

#### 1-((2R*,6S*)-6-(4-(Phenylsulfinyl)phenyl)-1,4-dioxan-2-yl)-*N*,*N*,*N*-trimethylmethanaminium Iodide (**7a**)

This compound was prepared starting from **37a** following the procedure described for **3a**: a white solid was obtained, which was recrystallized from EtOH (82% yield); mp 166–167 °C. ^1^H NMR (DMSO): δ 3.03–3.59 (m, 13H, CH_2_N, N(CH_3_)_3_ and dioxane), 3.78 (m, 1H, dioxane), 3.93 (dd, *J* = 10.2, 3.3 Hz, 1H, dioxane), 4.45 (m, 1H, dioxane), 4.88 (dd, *J* = 10.2, 2.5 Hz, 1H, dioxane), 7.42–7.81 (m, 9H, ArH). ESI/MS *m*/*z*: 360.2 [M]^+^. Anal. Calcd (C_20_H_26_INO_3_S) C, H, N, S.

#### 1-((2R*,6R*)-6-(4-(Phenylsulfinyl)phenyl)-1,4-dioxan-2-yl)-*N*,*N*,*N*-trimethylmethanaminium Iodide (**7b**)

This compound was prepared starting from **37b** following the procedure described for **3a**: a white solid was obtained, which was recrystallized from EtOH (79% yield); mp 173–174 °C. ^1^H NMR (DMSO): δ 3.10 (m, 9H, N(CH_3_)_3_), 3.24–3.98 (m, 5H, CH_2_N, dioxane), 4.22 (dd, *J* = 13.9, 9.7 Hz, 1H, dioxane), 4.46 (m, 1H, dioxane), 5.15 (dd, *J* = 9.3, 2.9 Hz, 1H, dioxane), 7.42–7.80 (m, 9H, ArH). ESI/MS *m*/*z*: 360.2 [M]^+^. Anal. Calcd (C_20_H_26_INO_3_S) C, H, N, S.

#### 1-((2R*,6S*)-6-(4-(Phenylsulfonyl)phenyl)-1,4-dioxan-2-yl)-*N*,*N*,*N*-trimethylmethanaminium Iodide (**8a**)

This compound was prepared starting from **38a** following the procedure described for **3a**: a white solid was obtained, which was recrystallized from EtOH (87% yield); mp 128–129 °C. ^1^H NMR (DMSO): δ 3.00–3.55 (m, 13H, CH_2_N, N(CH_3_)_3_, dioxane), 3.74 (dd, *J* = 11.2, 2.2 Hz, 1H, dioxane), 3.94 (dd, *J* = 11.5, 2.5 Hz, 1H, dioxane), 4.43 (m, 1H, dioxane), 4.93 (dd, *J* = 10.5, 2.6 Hz, 1H, dioxane), 7.51–8.02 (m, 9H, ArH). ESI/MS *m*/*z*: 376.2 [M]^+^. Anal. Calcd (C_20_H_26_INO_4_S) C, H, N, S.

#### 1-((2R*,6R*)-6-(4-(Phenylsulfonyl)phenyl)-1,4-dioxan-2-yl)-*N*,*N*,*N*-trimethylmethanaminium Iodide (**8b**)

This compound was prepared starting from **38b** following the procedure described for **3a**: a white solid was obtained, which was recrystallized from EtOH (86% yield); mp 231–232 °C. ^1^H NMR (DMSO): δ 3.11 (s, 9H, N(CH_3_)_3_), 3.38–3.72 (m, 3H, CH_2_N, dioxane), 3.78 (dd, *J* = 11.7, 3.6 Hz, 1H, dioxane), 3.92 (dd, *J* = 11.5, 2.8 Hz, 1H, dioxane), 4.26 (dd, *J* = 13.5, 10.1 Hz, 1H, dioxane), 4.51 (m, 1H, dioxane), 5.21 (dd, *J* = 8.8, 2.8 Hz, 1H, dioxane), 7.52–7.99 (m, 9H, ArH). ESI/MS *m*/*z*: 376.2 [M]^+^. Anal. Calcd (C_20_H_26_INO_4_S) C, H, N, S.

#### 1-((2R*,5R*)-5-Phenyl-1,4-dioxan-2-yl)-*N*,*N*,*N*-trimethylmethanaminium Iodide (**9a**)

This compound was prepared starting from **57a** following the procedure described for **3a**: a white solid was obtained, which was recrystallized from EtOH (92% yield); mp 204–205 °C. ^1^H NMR (DMSO): δ 3.12 (s, 9H, N(CH_3_)_3_), 3.42 (m, 2H, CH_2_N), 3.70–3.96 (m, 3H, dioxane), 4.13–4.42 (m, 2H, dioxane), 4.66 (dd, *J* = 8.2, 3.0 Hz, 1H, dioxane), 7.31–7.47 (m, 5, ArH). ESI/MS *m*/*z*: 236.2 [M]^+^, 599.2 [2M + I]^+^. Anal. Calcd (C_14_H_22_INO_2_) C, H, N.

#### 1-((2R*,5S*)-5-Phenyl-1,4-dioxan-2-yl)-*N*,*N*,*N*-trimethylmethanaminium Iodide (**9b**)

This compound was prepared starting from **57b** following the procedure described for **3a**: a white solid was obtained, which was recrystallized from EtOH (93% yield); mp 216–217 °C. ^1^H NMR (DMSO): δ 3.17 (m, 9H, N(CH_3_)_3_), 3.36–3.62 (m, 4H, CH_2_N, dioxane), 3.89 (m, 2H, dioxane), 4.29 (m, 1H, dioxane), 4.57 (dd, *J* = 10.3, 2.6 Hz, 1H, dioxane), 7.26–7.42 (m, 5H, ArH). ESI/MS *m*/*z*: 236.2 [M]^+^, 599.2 [2M + I]^+^. Anal. Calcd (C_14_H_22_INO_2_) C, H, N.

#### 1-(5,5-Diphenyl-1,4-dioxan-2-yl)-*N*,*N*,*N*-trimethylmethanaminium Iodide (**10**)

This compound was prepared starting from **58** following the procedure described for **3a**: a white solid was obtained, which was recrystallized from 2-PrOH (93% yield); mp 255–256 °C. ^1^H NMR (DMSO): δ 3.08 (m, 9H, N(CH_3_)_3_), 3.18–3.34 (m, 3H, CH_2_N, dioxane), 3.75 (dd, *J* = 11.2, 2.7 Hz, 1H, dioxane), 3.86 (d, *J* = 12.1 Hz, 1H, dioxane), 4.38 (m, 1H, dioxane), 4.85 (d, *J* = 12.1 Hz, 1H, dioxane), 7.19–7.57 (m, 10H, ArH). ESI/MS *m*/*z*: 312.2 [M]^+^, 751.3 [2M + I]^+^. Anal. Calcd (C_20_H_26_INO_2_) C, H, N.

#### 1-((2R*,5R*)-5-Cyclohexyl-5-phenyl-1,4-dioxan-2-yl)-*N*,*N*,*N*-trimethylmethanaminium Iodide (**11a**)

This compound was prepared starting from **59a** following the procedure described for **3a**: a white solid was obtained, which was recrystallized from 2-PrOH (75% yield); mp 161–162 °C. ^1^H NMR (DMSO): δ 0.53–1.82 (m, 11H, cyclohexyl), 2.98–3.21 (m, 12H, N(CH_3_)_3_, CH_2_N, dioxane), 3.54 (dd, *J* = 11.5, 2.6 Hz, 1H, dioxane), 3.88 (d, *J* = 12.4 Hz, 1H, dioxane), 4.21 (m, 1H, dioxane), 4.66 (d, *J* = 12.4 Hz, 1H, dioxane), 7.21–7.44 (m, 5H, ArH). ESI/MS *m*/*z*: 318.2 [M]^+^, 763.4 [2M + I]^+^. Anal. Calcd (C_20_H_32_INO_2_) C, H, N.

#### 1-((2R*,5S*)-5-Cyclohexyl-5-phenyl-1,4-dioxan-2-yl)-*N*,*N*,*N*-trimethylmethanaminium Iodide (**11b**)

This compound was prepared starting from **59b** following the procedure described for **3a**: a white solid was obtained, which was recrystallized from EtOH (79% yield); mp 175–176 °C. ^1^H NMR (DMSO): δ 0.40–2.24 (m, 11H, cyclohexyl), 3.02–3.82 (m, 14H, N(CH_3_)_3_, CH_2_N, dioxane), 4.18 (m, 1H, dioxane), 4.45 (d, *J* = 12.3 Hz, 1H, dioxane), 7.18–7.42 (m, 5H, ArH). ESI/MS *m*/*z*: 318.2 [M]^+^, 763.4 [2M + I]^+^. Anal. Calcd (C_20_H_32_INO_2_) C, H, N.

#### 1-((2R*,5R*)-5-Benzhydryl-1,4-dioxan-2-yl)-*N*,*N*,*N*-trimethylmethanaminium Iodide (**12a**)

This compound was prepared starting from **60a** following the procedure described for **3a**: a white solid was obtained, which was recrystallized from EtOH (74% yield); mp 218–219 °C. ^1^H NMR (DMSO): δ 2.90–3.68 (m, 15H, N(CH_3_)_3_, CH_2_N, dioxane), 3.92 (m, 1H, dioxane), 4.28 (d, 1H, CH(Ar)_2_), 4.56 (m, 1H, dioxane), 7.05–7.62 (m, 10H, ArH). ESI/MS *m*/*z*: 326.2 [M]^+^ Anal. Calcd (C_21_H_28_INO_2_) C, H, N.

#### 1-((2R*,5S*)-5-Benzhydryl-1,4-dioxan-2-yl)-*N*,*N*,*N*-trimethylmethanaminium Iodide (**12b**)

This compound was prepared starting from **60b** following the procedure described for **3a**: a white solid was obtained, which was recrystallized from MeOH (81% yield); mp 266–267 °C. ^1^H NMR (DMSO): δ 2.95–3.46 (m, 14H, N(CH_3_)_3_, CH_2_N, dioxane), 3.70 (m, 1H, dioxane), 3.91 (d, 1H, CH(Ar)_2_), 4.12 (m, 1H, dioxane), 4.39 (d, *J* = 10.6, 2.8 Hz, 1H, dioxane), 7.08–7.46 (m, 10H, ArH). ESI/MS *m*/*z*: 326.2 [M]^+^. Anal. Calcd (C_21_H_28_INO_2_) C, H, N.

#### 1-((2R*,5R*)-5-([1,1′-Biphenyl]-4-yl)-1,4-dioxan-2-yl)-*N*,*N*,*N*-trimethylmethanaminium Iodide (**13a**)

This compound was prepared starting from **61a** following the procedure described for **3a**: a white solid was obtained, which was recrystallized from EtOH (91% yield); mp 257–259 °C. ^1^H NMR (DMSO): δ 3.02–3.54 (m, 11H, N(CH_3_)_3_, CH_2_N), 3.71–4.01 (m, 3H, dioxane), 4.19 (dd, *J* = 13.7, 10.0 Hz, 1H, dioxane), 4.40 (m, 1H, dioxane), 4.63 (dd, *J* = 8.3, 2.9 Hz, 1H, dioxane), 7.28–7.78 (m, 9H, ArH). ESI/MS *m*/*z*: 312.2 [M]^+^. Anal. Calcd (C_20_H_26_INO_2_) C, H, N.

#### 1-((2R*,5S*)-5-([1,1′-Biphenyl]-4-yl)-1,4-dioxan-2-yl)-*N*,*N*,*N*-trimethylmethanaminium Iodide (**13b**)

This compound was prepared starting from **61b** following the procedure described for **3a**: a white solid was obtained, which was recrystallized from MeOH (91% yield); mp 300–301 °C. ^1^H NMR (DMSO): δ 3.05–3.68 (m, 14H, N(CH_3_)_3_, CH_2_N, dioxane), 3.91 (m, 1H, dioxane), 4.31 (m, 1H, dioxane), 4.60 (d, *J* = 10.2, 2.6 Hz, 1H, dioxane), 7.25–7.72 (m, 9H, ArH). ESI/MS *m*/*z*: 312.2 [M]^+^, 751.3 [2M + I]^+^. Anal. Calcd (C_20_H_26_INO_2_) C, H, N.

#### 1-((2R*,5R*)-5-(4-Benzylphenyl)-1,4-dioxan-2-yl)-*N*,*N*,*N*-trimethylmethanaminium Iodide (**14a**)

This compound was prepared starting from **62a** following the procedure described for **3a**: a white solid was obtained, which was recrystallized from MeOH (88% yield); mp 161–162 °C. ^1^H NMR (DMSO): δ 3.00–3.92 (m, 14H, N(CH_3_)_3_, CH_2_N, dioxane), 3.94 (s, 2H, CH_2_Ar), 4.12 (m, 1H, dioxane), 4.38 (m, 1H, dioxane), 4.60 (m, 1H, dioxane), 7.08–7.42 (m, 9H, ArH). ESI/MS *m*/*z*: 326.2 [M]^+^. Anal. Calcd (C_21_H_28_INO_2_) C, H, N.

#### 1-((2R*,5S*)-5-(4-Benzylphenyl)-1,4-dioxan-2-yl)-*N*,*N*,*N*-trimethylmethanaminium Iodide (**14b**)

This compound was prepared starting from **62b** following the procedure described for **3a**: a white solid was obtained, which was recrystallized from MeOH (83% yield); mp 206–207 °C. ^1^H NMR (DMSO): δ 3.02 (s, 9H, N(CH_3_)_3_), 3.30–3.62 (m, 4H, CH_2_N, dioxane), 3.78–3.88 (m, 2H, dioxane), 3.93 (s, 2H, CH_2_Ar), 4.29 (m, 1H, dioxane), 4.50 (dd, *J* = 10.4, 2.7 Hz, 1H, dioxane), 7.09–7.34 (m, 9H, ArH). ESI/MS *m*/*z*: 326.2 [M]^+^. Anal. Calcd (C_21_H_28_INO_2_) C, H, N.

#### 1-((2R*,5R*)-5-(4-(Phenylthio)phenyl)-1,4-dioxan-2-yl)-*N*,*N*,*N*-trimethylmethanaminium Iodide (**15a**)

This compound was prepared starting from **63a** following the procedure described for **3a**: a white solid was obtained, which was recrystallized from EtOH (83% yield); mp 168–169 °C. ^1^H NMR (DMSO): δ 3.02–3.29 (m, 11H, N(CH_3_)_3_, CH_2_N), 3.65–3.97 (m, 3H, dioxane), 4.14 (dd, *J* = 13.9, 9.9 Hz, 1H, dioxane), 4.39 (m, 1H, dioxane), 4.66 (dd, *J* = 8.4, 3.2 Hz, 1H, dioxane), 7.20–7.44 (m, 9H, ArH). ESI/MS *m*/*z*: 344.2 [M]^+^, 815.3 [2M + I]^+^. Anal. Calcd (C_20_H_26_INO_2_S) C, H, N, S.

#### 1-((2R*,5S*)-5-(4-(Phenylthio)phenyl)-1,4-dioxan-2-yl)-*N*,*N*,*N*-trimethylmethanaminium Iodide (**15b**)

This compound was prepared starting from **63b** following the procedure described for **3a**: a white solid was obtained, which was recrystallized from EtOH (91% yield); mp 173–175 °C. ^1^H NMR (DMSO): δ 3.01–3.62 (m, 14H, N(CH_3_)_3_, CH_2_N, dioxane), 3.90 (dd, *J* = 11.6, 3.4 Hz, 1H, dioxane), 4.27 (m, 1H, dioxane), 4.58 (dd, *J* = 10.4, 2.6 Hz, 1H, dioxane), 7.20–7.46 (m, 9H, ArH). ESI/MS *m*/*z*: 344.2 [M]^+^. Anal. Calcd (C_20_H_26_INO_2_S) C, H, N, S.

#### 1-((2R*,5R*)-5-(4-(Phenylsulfinyl)phenyl)-1,4-dioxan-2-yl)-*N*,*N*,*N*-trimethylmethanaminium Iodide (**16a**)

This compound was prepared starting from **64a** following the procedure described for **3a**: a white solid was obtained, which was recrystallized from EtOH (91% yield); mp 78–80 °C. ^1^H NMR (DMSO): δ 2.95–3.94 (m, 14H, N(CH_3_)_3_, CH_2_N, dioxane), 4.10 (dd, *J* = 13.9, 9.9 Hz, 1H, dioxane), 4.38 (m, 1H, dioxane), 4.72 (dd, *J* = 7.6, 3.5 Hz, 1H, dioxane), 7.38–7.83 (m, 9H, ArH). ESI/MS *m*/*z*: 360.2 [M]^+^. Anal. Calcd (C_20_H_26_INO_3_S) C, H, N, S.

#### 1-((2R*,5S*)-5-(4-(Phenylsulfinyl)phenyl)-1,4-dioxan-2-yl)-*N*,*N*,*N*-trimethylmethanaminium Iodide (**16b**)

This compound was prepared starting from **64b** following the procedure described for **3a**: a white solid was obtained, which was recrystallized from EtOH (91% yield); mp 175–176 °C. ^1^H NMR (DMSO): δ 2.91–3.59 (m, 14H, CH_2_N, N(CH_3_)_3_, dioxane), 3.86 (m, 1H, dioxane), 4.25 (m, 1H, dioxane), 4.60 (dd, *J* = 10.4, 2.6 Hz, 1H, dioxane), 7.40–7.81 (m, 9H, ArH). ESI/MS *m*/*z*: 360.2 [M]^+^. Anal. Calcd (C_20_H_26_INO_3_S) C, H, N, S.

#### 1-((2R*,5R*)-5-(4-(Phenylsulfonyl)phenyl)-1,4-dioxan-2-yl)-*N*,*N*,*N*-trimethylmethanaminium Iodide (**17a**)

This compound was prepared starting from **65a** following the procedure described for **3a**: a white solid was obtained, which was recrystallized from EtOH (88% yield); mp 218–219 °C. ^1^H NMR (DMSO): δ 2.98–3.48 (m, 11H, N(CH_3_)_3_, CH_2_N), 3.67 (m, 1H, dioxane), 3.75–3.92 (m, 2H, dioxane), 4.08 (dd, *J* = 13.7, 9.7 Hz, 1H, dioxane), 4.39 (m, 1H, dioxane), 4.79 (dd, *J* = 10.3, 2.5 Hz, 1H, dioxane), 7.48–8.07 (m, 9H, ArH). ESI/MS *m*/*z*: 376.2 [M]^+^. Anal. Calcd (C_20_H_26_INO_4_S) C, H, N, S.

#### 1-((2R*,5S*)-5-(4-(Phenylsulfonyl)phenyl)-1,4-dioxan-2-yl)-*N*,*N*,*N*-trimethylmethanaminium Iodide (**17b**)

This compound was prepared starting from **65b** following the procedure described for **3a**: a white solid was obtained, which was recrystallized from EtOH (90% yield); mp 192–193 °C. ^1^H NMR (DMSO): δ 2.96–3.60 (m, 13H, N(CH_3_)_3_, CH_2_N, dioxane), 3.79–4.00 (m, 2H, dioxane), 4.29 (m, 1H, dioxane), 4.64 (dd, *J* = 10.1, 2.9 Hz, 1H, dioxane), 7.48–8.00 (m, 9H, ArH). ESI/MS *m*/*z*: 376.2 [M]^+^. Anal. Calcd (C_20_H_26_INO_4_S) C, H, N, S.

#### 1-((2R*,5S*,6S*)-5,6-Diphenyl-1,4-dioxan-2-yl)-*N*,*N*,*N*-trimethylmethanaminium Iodide (**18a**)

This compound was prepared starting from **69a** following the procedure described for **3a**: a white solid was obtained, which was recrystallized from 2-PrOH (90% yield); mp 190–191 °C. ^1^H NMR (DMSO): δ 3.07–3.68 (m, 12H, N(CH_3_)_3_, CH_2_N, dioxane), 3.90 (m, 1H, dioxane), 4.60 (m, 2H, dioxane), 5.06 (d, *J* = 11.0 Hz, 1H, dioxane), 6.85–7.37 (m, 10H, ArH). ESI/MS *m*/*z*: 312.2 [M]^+^, 751.3 [2M + I]^+^. Anal. Calcd (C_20_H_26_INO_2_) C, H, N.

#### 1-((2R*,5R*,6R*)-5,6-Diphenyl-1,4-dioxan-2-yl)-*N*,*N*,*N*-trimethylmethanaminium Iodide (**18b**)

This compound was prepared starting from **69b** following the procedure described for **3a**: a white solid was obtained, which was recrystallized from EtOH (81% yield); mp 239–240 °C. ^1^H NMR (DMSO): δ 3.02–3.68 (m, 12H, CH_2_N, N(CH_3_)_3_, dioxane), 3.97 (dd, *J* = 10.4 and 3.5 Hz, 1H, dioxane), 4.47 (d, *J* = 11.1 Hz, 1H, dioxane), 4.62 (m, 1H, dioxane), 4.78 (d, *J* = 11.1 Hz, 1H, dioxane), 6.92–7.32 (m, 10H, ArH). ESI/MS *m*/*z*: 312.2 [M]^+^, 751.3 [2M + I]^+^. Anal. Calcd (C_20_H_26_INO_2_) C, H, N.

#### 1-((2R*,5S*,6R*)-5,6-Diphenyl-1,4-dioxan-2-yl)-*N*,*N*,*N*-trimethylmethanaminium Iodide (**18c**)

This compound was prepared starting from **69c** following the procedure described for **3a**: a white solid was obtained, which was recrystallized from EtOH (80% yield); mp 193–194 °C. ^1^H NMR (DMSO): δ 2.97 (s, 9H, N(CH_3_)_3_), 3.40–3.61 (m, 2H, CH_2_N), 3.69 (dd, *J* = 11.2 and 10.0 Hz, 1H, dioxane), 4.12 (dd, *J* = 11.2 and 2.1 Hz, 1H, dioxane), 4.24 (m, 1H, dioxane), 5.22 (d, *J* = 3.6 Hz, 1H, dioxane), 5.39 (d, *J* = 3.6 Hz, 1H, dioxane), 7.05–7.58 (m, 10H, ArH). ESI/MS *m*/*z*: 312.2 [M]^+^, 751.3 [2M + I]^+^. Anal. Calcd (C_20_H_26_INO_2_) C, H, N.

#### 1-((2R*,5S*)-5,6,6-Triphenyl-1,4-dioxan-2-yl)-*N*,*N*,*N*-trimethylmethanaminium Iodide (**19a**)

This compound was prepared starting from **75a** following the procedure described for **3a**: a white solid was obtained, which was recrystallized from 2-PrOH (80% yield); mp 275–276 °C. ^1^H NMR (DMSO): δ 2.89–3.51 (m, 11H, N(CH_3_)_3_, CH_2_N), 3.62–4.18 (m, 3H, dioxane), 5.01 (s, 1H, dioxane), 6.62–7.62 (m, 15H, ArH). ESI/MS *m*/*z*: 388.2 [M]^+^. Anal. Calcd (C_26_H_30_INO_2_) C, H, N.

#### 1-((2R*,5R*)-5,6,6-Triphenyl-1,4-dioxan-2-yl)-*N*,*N*,*N*-trimethylmethanaminium Iodide (**19b**)

This compound was prepared starting from **75b** following the procedure described for **3a**: a white solid was obtained, which was recrystallized from 2-PrOH (82% yield); mp 262–263 °C. ^1^H NMR (DMSO): δ 3.01–3.52 (m, 11H, N(CH_3_)_3_, CH_2_N), 3.81–4.20 (m, 3H, dioxane), 6.15 (s, 1H, dioxane), 6.87–7.80 (m, 15H, ArH). ESI/MS *m*/*z*: 388.2 [M]^+^. Anal. Calcd (C_26_H_30_INO_2_) C, H, N.

#### 1-((2R*,6S*)-6-Cyclohexyl-6-phenyl-1,4-dioxan-2-yl)-*N*,*N*-dimethylmethanamine (**33a**)

A solution of **27a**(16) (0.19 g, 0.5 mmol) and dimethylamine (10 mL) in dry benzene (20 mL) was heated in a sealed tube for 72 h at 110 °C. After evaporation of the solvent, the residue was dissolved in CHCl_3_, which was washed with NaOH 2 N and dried over Na_2_SO_4_. The solvent was concentrated *in vacuo* to give a residue, which was purified by column chromatography, eluting with CHCl_3_/CH_3_OH (9.5:0.5). An oil was obtained (90% yield). ^1^H NMR (CDCl_3_): δ 0.60–1.92 (m, 11H, cyclohexyl), 2.33 (s, 6H, N(CH_3_)_2_), 2.34–2.54 (m, 2H, CH_2_N), 3.21 (dd, 1H, dioxane), 3.40 (d, 1H, dioxane), 3.89 (dd, 1H, dioxane), 4.19 (m, 1H, dioxane), 4.46 (d, 1H, dioxane), 7.21–7.32 (m, 5H, ArH).

#### 1-((2R*,6R*)-6-Cyclohexyl-6-phenyl-1,4-dioxan-2-yl)-*N*,*N*-dimethylmethanamine (**33b**)

This compound was prepared starting from **27b**(16) following the procedure described for **33a**: an oil was obtained (91% yield). ^1^H NMR (CDCl_3_): δ 0.61–1.89 (m, 11H, cyclohexyl), 2.15–2.46 (m, 8H, CH_2_N, N(CH_3_)_2_), 3.28 (t, *J* = 11.3 Hz, 1H, dioxane), 3.60–3.85 (m, 3H, dioxane), 4.52 (d, *J* = 12.1 Hz, 1H, dioxane), 7.20–7.52 (m, 5H, ArH). The free base was transformed into the oxalate salt, which was crystallized from EtOH: mp 142–143 °C. ^1^H NMR (DMSO): δ 0.65–1.83 (m, 11H, cyclohexyl), 2.76 (s, 6H, N(CH_3_)_2_), 2.90–3.11 (m, 2H, CH_2_N), 3.16 (t, *J* = 10.9 Hz, 1H, dioxane), 3.55 (m, 2H, dioxane), 3.78 (m, 1H, dioxane), 4.62 (d, *J* = 12.2 Hz, 1H, dioxane), 7.21–7.48 (m, 5H, ArH), 8.21 (br s, 2H, COOH). ^13^C NMR (DMSO): δ 26.4, 26.4, 26.5, 26.5, 27.2, 44.1 (cyclohexyl); 47.6 (N(CH_3_)_2_); 57.8, 65.5, 68.2, 69.5, 80.0 (CH_2_N and dioxane); 127.4, 128.3, 128.5 (ArH); 140.0 (Ar); 164.5 (COOH). ESI/MS *m*/*z*: 304.2 [M + H]^+^, 326.2 [M + Na]^+^ Anal. Calcd (C_19_H_29_NO_2_^.^C_2_H_2_O_4_) C, H, N.

#### Enantiomeric Resolution of (±)-**33b**

The enantiomers of (±)-**33b** were separated by chiral HPLC by using a Regis Technologies Whelk-O 1 (*R*,*R*) H (25 cm × 2 cm, 10 μm particle size) column; mobile phase: *n*-hexane/2-propanol 85/15% v/v; flow rate 18 mL/min; detection was monitored at a wavelength of 220 nM. Retention times: 5.6 min for compound (−)-**33b** and 11.4 min for compound (+)-**33b**. ee >99.5% for both enantiomers.

(2*S*,6*S*)-(−)-**33b**: [α]_D_^20^ = −31.2 (c 1, CHCl_3_). The ^1^H NMR spectrum was identical to that of racemic compound (±)-**33b**. The free base was transformed into the oxalate salt, which was recrystallized from EtOH: [α]_D_^20^ = +47.7 (c 1, CH_3_OH), mp 142–143 °C. Anal. Calcd (C_21_H_31_NO_6_) C, H, N.

(2*R*,6*R*)-(+)-**33b**: [α]_D_^20^ = +31.5 (c 1, CHCl_3_). The ^1^H NMR spectrum was identical to that of racemic compound (±)-**33b**. The free base was transformed into the oxalate salt, which was recrystallized from EtOH: [α]_D_^20^ = +46.9 (c 1, CH_3_OH), mp 142–143 °C. Anal. Calcd (C_21_H_31_NO_6_) C, H, N.

#### 1-((2R*,6S*)-6-([1,1′-Biphenyl]-4-yl)-1,4-dioxan-2-yl)-*N*,*N*-dimethylmethanamine (**34a**)

This compound was prepared starting from **28a** following the procedure described for **33a**: an oil was obtained (91% yield). ^1^H NMR (CDCl_3_): δ 2.32 (s, 6H, N(CH_3_)_2_), 2.45 (m, 2H, CH_2_N), 3.36–3.47 (m, 2H, dioxane), 3.85–3.95 (m, 3H, dioxane), 4.72 (dd, 1H, dioxane), 7.34–7.62 (m, 9H, ArH).

#### 1-((2R*,6R*)-6-([1,1′-Biphenyl]-4-yl)-1,4-dioxan-2-yl)-*N*,*N*-dimethylmethanamine (**34b**)

This compound was prepared starting from **28b** following the procedure described for **33a**: an oil was obtained (93% yield). ^1^H NMR (CDCl_3_): δ 2.29 (s, 6H, N(CH_3_)_2_), 2.63 (m, 2H, CH_2_N), 3.66–4.00 (m, 5H, dioxane), 4.89 (dd, 1H, dioxane), 7.32–7.62 (m, 9H, ArH).

#### 1-((2R*,6S*)-6-(4-Benzylphenyl)-1,4-dioxan-2-yl)-*N*,*N*-dimethylmethanamine and 1-((2R*,6R*)-6-(4-benzylphenyl)-1,4-dioxan-2-yl)-*N*,*N*-dimethylmethanamine (**35a/b**)

This mixture of cis/trans (6:4) diastereomers was prepared starting from **29a/b** following the procedure described for **33a**: an oil was obtained (91% yield). ^1^H NMR (CDCl_3_): δ 2.28 (s, 6H trans, N(CH_3_)_2_), 2.32 (s, 6H cis, N(CH_3_)_2_), 2.47 (m, 2H cis, CH_2_N), 2.67 (m, 2H trans, CH_2_N), 3.28–4.05 (m, 7H cis + 7H trans, dioxane and CH_2_Ar), 4.65 (dd, 1H cis, dioxane), 4.80 (dd, 1H trans, dioxane), 7.08–7.39 (m, 9H cis + 9H trans, ArH).

#### 1-((2R*,6S*)-6-(4-(Phenylthio)phenyl)-1,4-dioxan-2-yl)-*N*,*N*-dimethylmethanamine (**36a**)

This compound was prepared starting from **30a** following the procedure described for **33a**: an oil was obtained (90% yield). ^1^H NMR (CDCl_3_): δ 2.32 (s, 6H, N(CH_3_)_2_), 2.43 (m, 2H, CH_2_N), 3.32 (m, 2H, dioxane), 3.80–4.05 (m, 3H, dioxane), 4.66 (dd, *J* = 2.7, 10.6 Hz, 1H, dioxane) 7.20–7.38 (m, 9H, ArH).

#### 1-((2R*,6R*)-6-(4-(Phenylthio)phenyl)-1,4-dioxan-2-yl)-*N*,*N*-dimethylmethanamine (**36b**)

This compound was prepared starting from **30b** following the procedure described for **33a**: an oil was obtained (92% yield). ^1^H NMR (CDCl_3_): δ 2.28 (s, 6H, N(CH_3_)_2_), 2.62 (m, 2H, CH_2_N), 3.64–3.98 (m, 5H, dioxane), 4.81 (dd *J* = 3.3, 8.0 Hz, 1H, dioxane) 7.27–7.40 (m, 9H, ArH).

#### 1-((2R*,6S*)-6-(4-(Phenylsulfinyl)phenyl)-1,4-dioxan-2-yl)-*N*,*N*-dimethylmethanamine (**37a**)

This compound was prepared starting from **31a** following the procedure described for **33a**: an oil was obtained (90% yield). ^1^H NMR (CDCl_3_): δ 2.28 (s, 6H, N(CH_3_)_2_), 2.41 (m, 2H, CH_2_N), 3.23 (m, 2H, dioxane), 3.80–4.00 (m, 3H, dioxane), 4.68 (dd, 1H, dioxane), 7.38–7.65 (m, 9H, ArH).

#### 1-((2R*,6R*)-6-(4-(Phenylsulfinyl)phenyl)-1,4-dioxan-2-yl)-*N*,*N*-dimethylmethanamine (**37b**)

This compound was prepared starting from **31b** following the procedure described for **33a**: an oil was obtained (93% yield). ^1^H NMR (CDCl_3_): δ 2.22 (s, 6H, N(CH_3_)_2_), 2.58 (m, 2H, CH_2_N), 3.60–3.98 (m, 5H, dioxane), 4.82 (dd, 1H, dioxane), 7.41–7.68 (m, 9H, ArH).

#### 1-((2R*,6S*)-6-(4-(Phenylsulfonyl)phenyl)-1,4-dioxan-2-yl)-*N*,*N*-diimethylmethanamine (**38a**)

This compound was prepared starting from **32a** following the procedure described for **33a**: an oil was obtained (90% yield). ^1^H NMR (CDCl_3_): δ 2.28 (s, 6H, N(CH_3_)_2_), 2.41 (m, 2H, CH_2_N), 3.20–3.40 (m, 2H, dioxane), 3.80–4.01 (m, 3H, dioxane), 4.72 (dd, 1H, dioxane), 7.42–8.00 (m, 9H, ArH).

#### 1-((2R*,6R*)-6-(4-(Phenylsulfonyl)phenyl)-1,4-dioxan-2-yl)-*N*,*N*-dimethylmethanamine (**38b**)

This compound was prepared starting from **32b** following the procedure described for **33a**: an oil was obtained (90% yield). ^1^H NMR (CDCl_3_): δ 2.24 (s, 6H, N(CH_3_)_2_), 2.60 (m, 2H, CH_2_N), 3.61–3.98 (m, 5H, dioxane), 4.86 (dd, 1H, dioxane), 7.44–7.99 (m, 9H, ArH).

#### 1-((2R*,5R*)-5-Phenyl-1,4-dioxan-2-yl)-*N*,*N*-diimethylmethanamine (**57a**)

This compound was prepared starting from **48a**(13) following the procedure described for **33a**: an oil was obtained (95% yield). ^1^H NMR (CDCl_3_): δ 2.31 (s, 6H, N(CH_3_)_2_), 2.38 (m, 2H, CH_2_N), 2.85 (dd, 1H, dioxane), 3.68–3.99 (m, 4H, dioxane), 4.62 (dd, 1H, dioxane), 7.25–7.46 (m, 5H, ArH).

#### 1-((2R*,5S*)-5-Phenyl-1,4-dioxan-2-yl)-*N*,*N*-dimethylmethanamine (**57b**)

This compound was prepared starting from **48b**(13) following the procedure described for **33a**: an oil was obtained (85% yield). ^1^H NMR (CDCl_3_): δ 2.19–2.53 (m, 8H, CH_2_N, N(CH_3_)_2_), 3.54 (dd, 1H, dioxane), 3.75–4.03 (m, 4H, dioxane), 4.58 (dd, 1H, dioxane), 7.30–7.40 (m, 5H, ArH).

#### 1-(5,5-Diphenyl-1,4-dioxan-2-yl)-*N*,*N*-dimethylmethanamine (**58**)

This compound was prepared starting from **49** following the procedure described for **33a**: an oil was obtained (75% yield). ^1^H NMR (CDCl_3_): δ 1.98–2.42 (m, 8H, CH_2_N, N(CH_3_)_2_), 3.30 (dd, 1H, dioxane), 3.68 (m, 2H, dioxane), 3.87 (m, 1H, dioxane), 4.61 (d, 1H, dioxane), 7.12–7.52 (m, 10H, ArH).

#### 1-((2R*,5R*)-5-Cyclohexyl-5-phenyl-1,4-dioxan-2-yl)-*N*,*N*-dimethylmethanamine (**59a**)

This compound was prepared starting from **50a** following the procedure described for **33a**: an oil was obtained (80% yield). ^1^H NMR (CDCl_3_): δ 0.58–1.88 (m, 11H, cyclohexyl), 2.00–2.40 (m, 8H, CH_2_N, N(CH_3_)_2_), 3.28 (dd, 1H, dioxane), 3.55 (dd, 1H, dioxane), 3.71–3.91 (m, 2H, dioxane), 4.60 (d, 1H, dioxane), 7.22–7.42 (m, 5H, ArH).

#### 1-((2R*,5S*)-5-Cyclohexyl-5-phenyl-1,4-dioxan-2-yl)-N,*N*-diimethylmethanamine(**59b**)

This compound was prepared starting from **50b** following the procedure described for **33a**: an oil was obtained (85% yield). ^1^H NMR (CDCl_3_): δ 0.58–1.87 (m, 11H, cyclohexyl), 2.18–2.80 (m, 8H, N(CH_3_)_2_, CH_2_N), 3.58–3.82 (m, 4H, dioxane), 4.42 (d, 1H, dioxane), 7.21–7.40 (m, 5H, ArH).

#### 1-((2R*,5R*)-5-Benzhydryl-1,4-dioxan-2-yl)-*N*,*N*-dimethylmethanamine (**60a**)

This compound was prepared starting from **51a** following the procedure described for **33a**: an oil was obtained (85% yield). ^1^H NMR (CDCl_3_): δ 2.19–2.73 (m, 8H, CH_2_N, N(CH_3_)_2_), 3.57–3.78 (m, 5H, dioxane), 4.30 (m, 1H, dioxane), 4.42 (d, 1H, CH(Ar)_2_), 7.14–7.40 (m, 10H, ArH).

#### 1-((2R*,5S*)-5-Benzhydryl-1,4-dioxan-2-yl)-*N*,*N*-dimethylmethanamine (**60b**)

This compound was prepared starting from **51b** following the procedure described for **33a**: an oil was obtained (82% yield). ^1^H NMR (CDCl_3_): δ 2.10–2.47 (m, 8H, CH_2_N, N(CH_3_)_2_), 3.38 (m, 2H, dioxane), 3.60–3.92 (m, 4H, dioxane), 4.28 (m, 1H, dioxane), 7.12–7.40 (m, 10H, ArH).

#### 1-((2R*,5R*)-5-([1,1′-Biphenyl]-4-yl)-1,4-dioxan-2-yl)-*N*,*N*-dimethylmethanamine (**61a**)

This compound was prepared starting from **52a** following the procedure described for **33a**: an oil was obtained (85% yield). ^1^H NMR (CDCl_3_): δ 2.28–2.85 (m, 8H, CH_2_N, N(CH_3_)_2_), 3.72–4.08 (m, 5H, dioxane), 4.69 (dd, 1H, dioxane), 7.31–7.63 (m, 9H, ArH).

#### 1-((2R*,5S*)-5-([1,1′-Biphenyl]-4-yl)-1,4-dioxan-2-yl)-*N*,*N*-dimethylmethanamine (**61b**)

This compound was prepared starting from **52b** following the procedure described for **33a**: an oil was obtained (80% yield). ^1^H NMR (CDCl_3_): δ 2.18–2.56 (m, 8H, CH_2_N, N(CH_3_)_2_), 3.55 (m, 2H, dioxane), 3.83 (m, 1H, dioxane), 4.00 (m, 2H, dioxane), 4.62 (dd, 1H, dioxane), 7.30–7.62 (m, 9H, ArH).

#### 1-((2R*,5R*)-5-(4-Benzylphenyl)-1,4-dioxan-2-yl)-*N*,*N*-dimethylmethanamine (**62a**)

This compound was prepared starting from **53a** following the procedure described for **33a**: an oil was obtained (86% yield). ^1^H NMR (CDCl_3_): δ 2.30–2.92 (m, 8H, CH_2_N, N(CH_3_)_2_), 3.68–4.02 (m, 7H, dioxane, ArCH_2_Ar), 4.62 (dd, 1H, dioxane), 7.17–7.38 (m, 9H, ArH).

#### 1-((2R*,5S*)-5-(4-Benzylphenyl)-1,4-dioxan-2-yl)-*N*,*N*-dimethylmethanamine (**62b**)

This compound was prepared starting from **53b** following the procedure described for **33a**: an oil was obtained (81% yield). ^1^H NMR (CDCl_3_): δ 2.18–2.52 (m, 8H, CH_2_N, N(CH_3_)_2_), 3.52 (m, 2H dioxane), 3.81–4.02 (m, 5H, dioxane, ArCH_2_Ar), 4.54 (dd, 1H, dioxane), 7.16–7.36 (m, 9H, ArH).

#### 1-((2R*,5R*)-5-(4-(Phenylthio)phenyl)-1,4-dioxan-2-yl)-*N*,*N*-dimethylmethanamine (**63a**)

This compound was prepared starting from **54a** following the procedure described for **33a**: an oil was obtained (86% yield). ^1^H NMR (CDCl_3_): δ 2.32–2.88 (m, 8H, CH_2_N, N(CH_3_)_2_), 3.65–3.99 (m, 5H, dioxane), 4.62 (dd, 1H, dioxane), 7.20–7.40 (m, 9H, ArH).

#### 1-((2R*,5S*)-5-(4-(Phenylthio)phenyl)-1,4-dioxan-2-yl)-*N*,*N*-dimethylmethanamine (**63b**)

This compound was prepared starting from **54b** following the procedure described for **33a**: an oil was obtained (87% yield). ^1^H NMR (CDCl_3_): δ 2.17–2.52 (m, 8H, CH_2_N, N(CH_3_)_2_), 3.50 (m, 2H, dioxane), 3.72–4.02 (m, 3H, dioxane), 4.56 (dd, 1H, dioxane), 7.22–7.38 (m, 9H, ArH).

#### 1-((2R*,5R*)-5-(4-(Phenylsulfinyl)phenyl)-1,4-dioxan-2-yl)-*N*,*N*-dimethylmethanamine (**64a**)

This compound was prepared starting from **55a** following the procedure described for **33a**: a solid was obtained (84% yield). ^1^H NMR (CDCl_3_): δ 2.22–2.82 (m, 8H, N(CH_3_)_2_, CH_2_N), 3.60–3.95 (m, 5H, dioxane), 4.62 (dd, 1H, dioxane), 7.41–7.69 (m, 9H, ArH).

#### 1-((2R*,5S*)-5-(4-(Phenylsulfinyl)phenyl)-1,4-dioxan-2-yl)-*N*,*N*-dimethylmethanamine (**64b**)

This compound was prepared starting from **64b** following the procedure described for **3a**: a white solid was obtained (84% yield); mp 96–99 °C. ^1^H NMR (CDCl_3_): δ 2.16–2.47 (m, 8H, CH_2_N, N(CH_3_)_2_), 3.44 (m, 2H, dioxane), 3.77 (m, 1H, dioxane), 3.92 (m, 2H, dioxane), 4.55 (dd, 1H, dioxane), 7.38–7.65 (m, 9H, ArH).

#### 1-((2R*,5R*)-5-(4-(Phenylsulfonyl)phenyl)-1,4-dioxan-2-yl)-*N*,*N*-dimethylmethanamine (**65a**)

This compound was prepared starting from **56a** following the procedure described for **33a**: an oil was obtained (85% yield). ^1^H NMR (CDCl_3_): δ 2.28–2.81 (m, 8H, CH_2_N, N(CH_3_)_2_), 3.60–3.98 (m, 5H, dioxane), 4.66 (dd, 1H, dioxane), 7.46–7.99 (m, 9H, ArH).

#### 1-((2R*,5S*)-5-(4-(Phenylsulfonyl)phenyl)-1,4-dioxan-2-yl)-*N*,*N*-dimethylmethanamine (**65b**)

This compound was prepared starting from **56b** following the procedure described for **33a**: an oil was obtained (84% yield). ^1^H NMR (CDCl_3_): δ 2.16–2.50 (m, 8H, CH_2_N, N(CH_3_)_2_), 3.50 (m, 2H, dioxane), 3.70–4.02 (m, 3H, dioxane), 4.60 (dd, 1H, dioxane), 7.43–7.95 (m, 9H, ArH).

#### 1-((2R*,5S*,6S*)-5,6-Diphenyl-1,4-dioxan-2-yl)-*N*,*N*-diimethylmethanamine (**69a**)

Tosyl chloride (1.8 g, 9.4 mmol) was added to a stirred solution of **68a**(38) (2 g, 7.4 mmol) in pyridine (5 mL) at 0 °C over 30 min. After 3 h at 0 °C, the mixture was left for 20 h at 4 °C in the freezer. Then, it was poured into ice and concentrated HCl (5 mL) and extracted with CHCl_3_. The organic layers were washed with 2 N HCl (15 mL), NaHCO_3_ saturated solution (15 mL), and H_2_O (15 mL) and then dried over Na_2_SO_4_. The evaporation of the solvent afforded the intermediate tosyl derivative, which was used in the next step without further purification. Dimethylamine (10 mL) was added to a solution of tosyl derivative in dry benzene (20 mL), and the mixture was heated in a sealed tube for 72 h at 110 °C. After evaporation of the solvent, the residue was dissolved in CHCl_3_, which was washed with NaOH 2 N and dried over Na_2_SO_4_. The solvent was concentrated *in vacuo* to give a residue, which was purified by column chromatography, eluting with CHCl_3_/CH_3_OH (9.5:0.5). An oil was obtained (85% yield). ^1^H NMR (CDCl_3_): δ 2.32 (s, 6H, N(CH_3_)_2_), 2.52 (m, 2H, CH_2_N), 3.69 (dd, 1H, dioxane), 4.05–4.20 (m, 2H, dioxane), 4.37 (d, 1H, dioxane), 4.52 (d, 1H, dioxane), 6.96–7.23 (m, 10H, ArH).

#### 1-((2R*,5R*,6R*)-5,6-Diphenyl-1,4-dioxan-2-yl)-*N*,*N*-dimethylmethanamine (**69b**)

This compound was prepared starting from **68b**(38) following the procedure described for **69a**: an oil was obtained (80% yield). ^1^H NMR (CDCl_3_): δ 2.38 (m, 6, N(CH_3_)_2_), 2.82–3.12 (m, 2H, CH_2_N), 3.98–4.18 (m, 3H, dioxane), 4.40 (d, 1H, dioxane), 4.67 (d, 1H, dioxane), 6.97–7.25 (m, 10H, ArH).

#### 1-((2R*,5S*,6R*)-5,6-Diphenyl-1,4-dioxan-2-yl)-*N*,*N*-dimethylmethanamine (**69c**)

This compound was prepared starting from **68c** following the procedure described for **69a**: an oil was obtained (82% yield). ^1^H NMR (CDCl_3_): δ 2.14 (m, 6H, N(CH_3_)_2_), 2.18–2.46 (m, 2H, CH_2_N), 2.99 (dd, 1H, dioxane), 3.74 (dd, *J* = 11.5 and 10.3 Hz, 1H, dioxane), 3.98 (m, 1H, dioxane), 4.19 (dd, 1H, dioxane), 5.11 (d, 1H, dioxane), 5.20 (d, 1H, dioxane), 7.10–7.38 (m, 10H, ArH).

#### 1-((2R*,5S*)-5,6,6-Triphenyl-1,4-dioxan-2-yl)-*N*,*N*-dimethylmethanamine (**75a**)

This compound was prepared starting from **74a** following the procedure described for **69a**: an oil was obtained (72% yield). ^1^H NMR (CDCl_3_): δ 2.22 (s, 6H, N(CH_3_)_2_), 2.48 (m, 2H), 3.78 (dd, *J* = 11.2 Hz and *J* = 10.4 Hz, 1H, dioxane), 3.98 (m, 1H, dioxane), 4.22 (dd, 1H, dioxane), 4.95 (s, 1H, dioxane), 6.71–7.60 (m, 15H, ArH).

#### 1-((2R*,5R*)-5,6,6-Triphenyl-1,4-dioxan-2-yl)-*N*,*N*-diimethylmethanamine (**75b**)

This compound was prepared starting from **74b** following the procedure described for **69a**: an oil was obtained (75% yield). ^1^H NMR (CDCl_3_): δ 2.23–2.72 (m, 8H, CH_2_N, N(CH_3_)_2_), 3.40 (dd, 1H, dioxane), 3.58 (dd, *J* = 11.5 Hz and *J* = 10.3 Hz, 1H, dioxane), 3.99 (m, 1H, dioxane), 5.82 (s, 1H, dioxane), 6.87–7.72 (m, 15H, ArH).

### ECD and NMR Calculations

Merck molecular force field (MMFF) and DFT calculations were run with Spartan’18 (Wavefunction, Inc., Irvine CA, 2014), with standard parameters and convergence criteria. DFT and TDDFT calculations were run with Gaussian’16 (Rev. B.02, Gaussian, Inc., Wallingford CT, 2016),^56^ with default grids and convergence criteria. The calculations were run on the N-protonated forms of **2** and **33b** (charge +1). Conformational searches were run with the Monte Carlo algorithm implemented in Spartan’18 using MMFF. All structures thus obtained were first optimized with the DFT method using ωB97X-D functional and 6-31G(d) basis set *in vacuo* and then re-optimized using ωB97X-D functional and 6-31G+(d) basis set, first *in vacuo* then using the SMD solvent model for acetonitrile. TDDFT calculations were run using several combinations of functionals (ωB97X-D, B3LYP, CAM-B3LYP, wB97X-D, BH&HLYP, M11), basis sets (def2-SVP and def2-TZVP), either *in vacuo* or using IEF-PCM solvent model for acetonitrile; they included at least 16 excited states (roots). Boltzmann populations were estimated at 300 K from internal energies. ECD spectra were generated using the program SpecDis,^57,58^ by applying a Gaussian band shape with 0.25 eV exponential half-width, shifted by 15 nm, scaled by a factor 2, from dipole-length rotational strengths.

### Binding Studies

#### Cell Culture and Membrane Preparation

CHO-K1 cells stably transfected with the human muscarinic receptor subtypes (hM_1–5_) were grown in Dulbecco’s modified Eagle’s medium (DMEM) with nutrient mixture F12 (DMEM/F12, 50/50), containing 10% fetal bovine serum, penicillin (100 U/mL), streptomycin (100 U/mL), l-glutamine (4 mM), and geneticin (G-418, 50 μg/mL) at 37 °C in a 5% CO_2_ humidified incubator. In order to harvest the cells, the culture medium was removed;the cells were washed with PBS and then trypsinized by trypsin–EDTA treatment for 2–3 min. Serum (0.7 mL) was added to inactivate the trypsin, and the cells were spun down by centrifuging at 300*g* for 5 min. The cells were then resuspended in ice-cold 25 mM sodium phosphate buffer containing 5 mM MgCl_2_, pH 7.4 (binding buffer) and homogenized using a cell disrupter (Ultra-Turrax, setting 3, 30 s). The homogenate was sedimented by centrifugation (17,000*g*, 15 min). The supernatant was discarded, and the resulting membrane pellets were resuspended with Ultra-Turrax in the same buffer to give a final protein concentration of 1–2 mg/mL. The protein content was determined by the method of Bradford (1976) with bovine serum albumin (Sigma) as a standard and stored at −80 °C.

#### Inhibition Radioligand Binding Assay

Inhibition radioligand binding assays were conducted as previously described^48,49^ with 0.2 nM [^3^H]NMS in binding buffer in a final volume of 250 μL. Nonspecific binding was defined in the presence of 10 μM atropine. Briefly, membrane fractions (about 25–70 μg/mL of protein) were incubated with radioligand and unlabeled test compounds for 2 h at r.t. Bound and free radioactivity were separated by filtering the assay mixture through UniFilter GF/B plates using a FilterMate Cell Harvester (PerkinElmer Life and Analytical Science). The filter bound radioactivity was counted by a TopCount NXT Microplate Scintillation Counter (PerkinElmer Life and Analytical Science). Data (cpm) were normalized to percentage-specific binding and analyzed using a four-parameter logistic equation in GraphPad Prism 5.02; IC_50_ values were determined, and K_i_ values were calculated.^59^ The values reported in Tables 1 and 2 represent the arithmetic mean ± S.E.M. of at least three independent experiments, each one performed in duplicate.

### Docking Studies

Docking simulations involved the ligands with p*K*_i_ values on M_3_ mAChR greater than 6 and the recently resolved M_3_ mAChR structure in complex with a selective antagonist (PDB Id: 5ZHP).^52^ The protein structure was completed by adding hydrogen atoms, and the ionizable groups were set to be compatible to physiological pH using the VEGA suite of programs.^60^ The prepared structure was finally minimized by using the NAMD program^61^ and keeping fixed the backbone atoms to retain the experimental folding. The structure of the considered ligands was optimized by PM7-based semi-empirical calculations.^62^ Docking simulations were performed by PLANTS^63^ by focusing the searches within a 8.0 Å radius around the bound resolved antagonist. The simulations were carried out using the ChemPLP primary score with speed equal to 1 and 10 poses were generated for each ligand. The obtained complexes were optimized by using NAMD and by keeping fixed all atoms outside a 10 Å radius sphere around the docked ligand and then rescored by ReScore+.^64^

### Functional Studies on MSCs from Mouse Bone Marrow

#### MSC Collection and Culture

The *in vitro* studies were performed by using bone marrow MSCs as a model. Male BALB/c mice (Harlan Italy SrL, Milano, Italy) (8 weeks old; body weight ∼24.5 g; *n* = 4) were kept in a laminar-flow cage in a standardized environmental condition. Food (Harlan, Italy), and water was supplied ad libitum. Mice were sacrificed by CO_2_ narcosis and cervical dislocation in accordance with the recommendations of the Italian Ethical Committee and under the supervision of authorized investigators. Long bones (femurs and tibiae) were dissected and cleaned from skin, muscle, and connective tissues as much as possible. Bones were placed in a culture dish containing sterile PBS. Then, the bone cavity was flushed in DMEM with a syringe in order to collect the bone marrow cells into a 50 mL sterile tube. The procedure was repeated until all marrow was removed. Cell suspension was filtered through a cell strainer (70 μm size) to remove cell clumps or bone debris. Then, bone marrow cells were plated in 100 mm culture dishes in DMEM containing 10% heat-inactivated-fetal calf serum (HIFCS), penicillin, and streptomycin. In order to obtain a population of bone marrow MSCs, the protocol by Solimani and Nadri^65^ was followed. Cells were incubated at 37 °C with 5% CO_2_ in a humidified chamber. After 3 h, the nonadherent cells that accumulate on the surface of the dish were removed by changing the medium and replacing it with a fresh complete medium. After 8 h of culture, the medium was further replaced with fresh complete medium. The last step was repeated every 8 h for up to 72 h of initial culture. Then, the adherent cells were washed with sterile PBS and added with a fresh medium every 3–4 days. After 2 weeks of initiating culture, cells were washed with PBS, detached by trypsinization, counted, and plated at the density of 5,000 cells/well in 96 culture plates (Costar Corp., Milano, Italy) in DMEM containing 10% HIFCS, penicillin, and streptomycin.

#### Experimental Protocol

MSCs were treated with compound **3b** (from 10^–4^ to 10^–10^ M) for 24 h. Control cultures were performed by incubating the cells with the only vehicle (DMSO) or by untreated cells. Parallel other cultures were incubated with **3b** from 10^–4^ to 10^–10^ M for 1 h, and then, the culture medium was replaced with a fresh medium. The MSCs were maintained in the presence of carbachol at 10^–10^ M for 24 h. At the end of each procedure, the MSCs viability was measured by MTS assay. Specifically, cells were incubated with Cell Titer 96 Aqueous One Solution Reagent (Promega Italia, Milano, Italy) for 2 h in a humidified 5% CO_2_ atmosphere. The quantity of the formazan product was directly proportional to the number of living cells in culture. The colored formazan was measured by reading the absorbance at 490 nm using a 6-well plate reader.

## Supporting Information Available

^1^H NMR and ^13^C NMR spectra of **3a**, **3b**, and the oxalate salt of **33b**; ^1^H NMR spectra of **28a/b**, **52a/b**, and **75a/b**; ^1^H NMR and NOESY spectra of **11a** and **18c**; HPLC chromatograms of (±)-**3b** and its enantiomers; Table S1 reporting the elemental analysis results for compounds **3–19** and **33b** and enantiomers of **3b** and **33b**; and experimental procedures for the synthesis of intermediates **22**, **24**–**26**, **28–32**, **40–44**, **46**, **47**, **50–56**, **67**, **68c**, **70**, and **72–74** (PDF)
Molecular formula strings (CSV)
Atomic coordinates of (*R*)-**2** (PDB)
Atomic coordinates of (*S*)-**2** (PDB)
Atomic coordinates of (2*R*,6*R*)-**3b** (PDB)
Atomic coordinates of (2*S*,6*S*)-**3b** (PDB)
Atomic coordinates of (2*R*,6*R*)-**33b** (PDB)
Atomic coordinates of (2*S*,6*S*)-**33b** (PDB)

## Supplementary Material

jm9b02100_si_002.csv

jm9b02100_si_003.pdb

jm9b02100_si_004.pdb

jm9b02100_si_005.pdb

jm9b02100_si_006.pdb

jm9b02100_si_007.pdb

jm9b02100_si_008.pdb

## Abbreviations

mAChRs: muscarinic acetylcholine receptors
cAMP: adenosine 3′,5′-cyclic monophosphate
CNS: central nervous system
OAB: overactive bladder
DMSO: dimethyl sulfoxide
*m*-CPBA: *meta*-chloroperoxybenzoic acid
r.t.: room temperature
TBDMSCl: *tert*-butyldimethylsilyl chloride
e.e.: enantiomeric excess
ECD: electronic circular dichroism
TDDFT: time-dependent density functional theory
CHO: chinese hamster ovary
[^3^H]NMS: [^3^H]*N*-methylscopolamine
MSC: mesenchymal stem cells
mp: melting point.

## References

[ref1] Kruse A. C.; Kobilka B. K.; Gautam D.; Sexton P. M.; Christopoulos A.; Wess J. Muscarinic acetylcholine receptors: novel opportunities for drug development. Nat. Rev. Drug Discovery 2014, 13, 549–560. 10.1038/nrd4295. 24903776 PMC5818261

[ref2] Thomsen M.; Sørensen G.; Dencker D. Physiological roles of CNS muscarinic receptors gained from knockout mice. Neuropharmacology 2018, 136, 411–420. 10.1016/j.neuropharm.2017.09.011. 28911965 PMC5845799

[ref3] De Angelis F.; Tata A. M. Analgesic effects mediated by muscarinic receptors: mechanisms and pharmacological approaches. Cent. Nerv. Syst. Agents Med. Chem. 2016, 16, 218–226. 10.2174/1871524916666160302103033. 26931765

[ref4] Ehlert F. J.; Ostrom R. S.; Sawyer G. W. Subtypes of the muscarinic receptor in smooth muscle. Life Sci. 1997, 61, 1729–1740. 10.1016/s0024-3205(97)00433-5. 9365220

[ref5] Harvey R. D. Muscarinic receptor agonists and antagonists: effects on cardiovascular function. Muscarinic Receptors; Handbook of Experimental Pharmacology; Springer, 2012; Vol. 208, pp 299–316. 10.1007/978-3-642-23274-9_13 22222704

[ref6] Proctor G. B.; Carpenter G. H. Regulation of salivary gland function by autonomic nerves. Auton. Neurosci. 2007, 133, 3–18. 10.1016/j.autneu.2006.10.006. 17157080

[ref7] Landgraf D.; Barth M.; Layer P. G.; Sperling L. E. Acetylcholine as a possible signaling molecule in embryonic stem cells: studies on survival, proliferation and death. Chem. Biol. Interact. 2010, 187, 115–119. 10.1016/j.cbi.2010.03.007. 20223227

[ref8] Shah N.; Khurana S.; Cheng K.; Raufman J.-P. Muscarinic receptors and ligands in cancer. Am. J. Physiol. Cell Physiol. 2009, 296, C221–C232. 10.1152/ajpcell.00514.2008. 19036940 PMC2643856

[ref9] Razani-Boroujerdi S.; Behl M.; Hahn F. F.; Pena-Philippides J. C.; Hutt J.; Sopori M. L. Role of muscarinic receptors in the regulation of immune and inflammatory responses. J. Neuroimmunol. 2008, 194, 83–88. 10.1016/j.jneuroim.2007.11.019. 18190972 PMC2323336

[ref10] Grando S. A. Muscarinic receptor agonists and antagonists: effects on keratinocyte functions. Muscarinic Receptors; Handbook of Experimental Pharmacology; Springer, 2012; Vol. 208, pp 429–450. 10.1007/978-3-642-23274-9_18 22222709

[ref11] Hoogduijn M. J.; Cheng A.; Genever P. G. Functional nicotinic and muscarinic receptors on mesenchymal stem cells. Stem Cell. Dev. 2009, 18, 103–112. 10.1089/scd.2008.0032. 18393628

[ref12] Piovesana R.; Melfi S.; Fiore M.; Magnaghi V.; Tata A. M. M2 muscarinic receptor activation inhibits cell proliferation and migration of rat adipose-mesenchymal stem cells. J. Cell. Physiol. 2018, 233, 5348–5360. 10.1002/jcp.26350. 29227527

[ref13] Quaglia W.; Piergentili A.; Del Bello F.; Farande Y.; Giannella M.; Pigini M.; Rafaiani G.; Carrieri A.; Amantini C.; Lucciarini R.; Santoni G.; Poggesi E.; Leonardi A. Structure–Activity Relationships in 1,4-Benzodioxan-Related Compounds. 9.(1) From 1,4-Benzodioxane to 1,4-Dioxane Ring as a Promising Template of Novel α1D-Adrenoreceptor Antagonists, 5-HT1AFull Agonists, and Cytotoxic Agents. J. Med. Chem. 2008, 51, 6359–6370. 10.1021/jm800461k. 18817363

[ref14] Mammoli V.; Bonifazi A.; Del Bello F.; Diamanti E.; Giannella M.; Hudson A. L.; Mattioli L.; Perfumi M.; Piergentili A.; Quaglia W.; Titomanlio F.; Pigini M. Favourable involvement of α_2A_-adrenoreceptor antagonism in the I_2_-imidazoline binding sites-mediated morphine analgesia enhancement. Bioorg. Med. Chem. 2012, 20, 2259–2265. 10.1016/j.bmc.2012.02.016. 22370341

[ref15] Bonifazi A.; Piergentili A.; Del Bello F.; Farande Y.; Giannella M.; Pigini M.; Amantini C.; Nabissi M.; Farfariello V.; Santoni G.; Poggesi E.; Leonardi A.; Menegon S.; Quaglia W. Structure–activity relationships in 1,4-benzodioxan-related compounds. 11. Reversed enantioselectivity of 1,4-dioxane derivatives in α_1_-adrenergic and 5-HT_1A_ receptor binding sites recognition. J. Med. Chem. 2013, 56, 584–588. 10.1021/jm301525w. 23252794

[ref16] Bonifazi A.; Del Bello F.; Mammoli V.; Piergentili A.; Petrelli R.; Cimarelli C.; Pellei M.; Schepmann D.; Wünsch B.; Barocelli E.; Bertoni S.; Flammini L.; Amantini C.; Nabissi M.; Santoni G.; Vistoli G.; Quaglia W. Novel potent N-methyl-D-aspartate (NMDA) receptor antagonists or σ_1_ receptor ligands based on properly substituted 1,4-dioxane ring. J. Med. Chem. 2015, 58, 8601–8615. 10.1021/acs.jmedchem.5b01214. 26430967

[ref17] Del Bello F.; Bonifazi A.; Giannella M.; Giorgioni G.; Piergentili A.; Petrelli R.; Cifani C.; Micioni Di Bonaventura M. V.; Keck T. M.; Mazzolari A.; Vistoli G.; Cilia A.; Poggesi E.; Matucci R.; Quaglia W. The replacement of the 2-methoxy substituent of N-((6,6-diphenyl-1,4-dioxan-2-yl)methyl)-2-(2-methoxyphenoxy)ethan-1-amine improves the selectivity for 5-HT_1A_ receptor over α_1_-adrenoceptor and D_2_-like receptor subtypes. Eur. J. Med. Chem. 2017, 125, 233–244. 10.1016/j.ejmech.2016.09.026. 27662034

[ref18] Del Bello F.; Bonifazi A.; Giorgioni G.; Quaglia W.; Amantini C.; Morelli M. B.; Santoni G.; Battiti F. O.; Vistoli G.; Cilia A.; Piergentili A. Chemical manipulations on the 1,4-dioxane ring of 5-HT_1A_ receptor agonists lead to antagonists endowed with antitumor activity in prostate cancer cells. Eur. J. Med. Chem. 2019, 168, 461–473. 10.1016/j.ejmech.2019.02.056. 30844609

[ref19] Morelli M. B.; Amantini C.; Nabissi M.; Santoni G.; Wünsch B.; Schepmann D.; Cimarelli C.; Pellei M.; Santini C.; Fontana S.; Mammoli V.; Quaglia W.; Bonifazi A.; Giannella M.; Giorgioni G.; Piergentili A.; Del Bello F. Role of the NMDA receptor in the antitumor activity of chiral 1,4-dioxane ligands in MCF-7 and SKBR3 breast cancer cells. ACS Med. Chem. Lett. 2019, 10, 511–516. 10.1021/acsmedchemlett.8b00536. 30996788 PMC6466546

[ref20] Del Bello F.; Ambrosini D.; Bonifazi A.; Newman A. H.; Keck T. M.; Giannella M.; Giorgioni G.; Piergentili A.; Cappellacci L.; Cilia A.; Franchini S.; Quaglia W. Multitarget 1,4-dioxane compounds combining favorable D_2_-like and 5-HT_1A_ receptor interactions with potential for the treatment of Parkinson’s disease or schizophrenia. ACS Chem. Neurosci. 2019, 10, 2222–2228. 10.1021/acschemneuro.8b00677. 30609891 PMC8378419

[ref21] Piergentili A.; Quaglia W.; Giannella M.; Del Bello F.; Bruni B.; Buccioni M.; Carrieri A.; Ciattini S. Dioxane and oxathiane nuclei: suitable substructures for muscarinic agonists. Bioorg. Med. Chem. 2007, 15, 886–896. 10.1016/j.bmc.2006.10.040. 17084634

[ref22] Piergentili A.; Quaglia W.; Giannella M.; Del Bello F.; Buccioni M.; Matucci R.; Matucci R. Rapid novel divergent synthesis and muscarinic agonist profile of all four optical isomers of N,N,N-trimethyl(6-methyl-1,4-dioxan-2-yl)methanaminium iodide. Bioorg. Med. Chem. Lett. 2008, 18, 614–618. 10.1016/j.bmcl.2007.11.073. 18077164

[ref23] Piergentili A.; Quaglia W.; Del Bello F.; Giannella M.; Pigini M.; Barocelli E.; Bertoni S.; Matucci R.; Nesi M.; Bruni B.; Di Vaira M. Properly substituted 1,4-dioxane nucleus favours the selective M_3_ muscarinic receptor activation. Bioorg. Med. Chem. 2009, 17, 8174–8185. 10.1016/j.bmc.2009.10.027. 19896386

[ref24] Del Bello F.; Barocelli E.; Bertoni S.; Bonifazi A.; Camalli M.; Campi G.; Giannella M.; Matucci R.; Nesi M.; Pigini M.; Quaglia W.; Piergentili A. 1,4-Dioxane, a suitable scaffold for the development of novel M_3_ muscarinic receptor antagonists. J. Med. Chem. 2012, 55, 1783–1787. 10.1021/jm2013216. 22243489

[ref25] Del Bello F.; Bonifazi A.; Giorgioni G.; Petrelli R.; Quaglia W.; Altomare A.; Falcicchio A.; Matucci R.; Vistoli G.; Piergentili A. Novel muscarinic acetylcholine receptor hybrid ligands embedding quinuclidine and 1,4-dioxane fragments. Eur. J. Med. Chem. 2017, 137, 327–337. 10.1016/j.ejmech.2017.06.004. 28609709

[ref26] Abrams P.; Andersson K.-E. Muscarinic receptor antagonists for overactive bladder. BJU Int. 2007, 100, 987–1006. 10.1111/j.1464-410x.2007.07205.x. 17922784

[ref27] Campbell N.; Boustani M.; Limbil T.; Ott C.; Fox C.; Maidment I.; Schubert C. C.; Munger S.; Fick D.; Miller D.; Gulati R. The cognitive impact of anticholinergics: a clinical review. Clin. Interv. Aging 2009, 4, 225–333. 10.2147/cia.s5358. 19554093 PMC2697587

[ref28] Callegari E.; Malhotra B.; Bungay P. J.; Webster R.; Fenner K. S.; Kempshall S.; Laperle J. L.; Michel M. C.; Kay G. G. A comprehensive non-clinical evaluation of the CNS penetration potential of antimuscarinic agents for the treatment of overactive bladder. Br. J. Clin. Pharmacol. 2011, 72, 235–246. 10.1111/j.1365-2125.2011.03961.x. 21392072 PMC3162653

[ref29] Kessler T. M.; Bachmann L. M.; Minder C.; Löhrer D.; Umbehr M.; Schünemann H. J.; Kessels A. G. H. Adverse Event assessment of antimuscarinics for treating overactive bladder: a network meta-analytic approach. PLoS One 2011, 6, e16718 10.1371/journal.pone.0016718. 21373193 PMC3044140

[ref30] Portoghese P. S. Relationships between stereostructure and pharmacological activities. Annu. Rev. Pharmacol. 1970, 10, 51–76. 10.1146/annurev.pa.10.040170.000411. 4911023

[ref31] Molander G. A.; Elia M. D. Suzuki–Miyaura Cross-Coupling Reactions of Benzyl Halides with Potassium Aryltrifluoroborates. J. Org. Chem. 2006, 71, 9198–9202. 10.1021/jo061699f. 17109547 PMC2515367

[ref32] Corey E. J.; Chaykovsky M. Dimethyloxosulfonium methylide ((CH_3_)_2_SOCH_2_) and dimethylsulfonium methylide ((CH_3_)_2_SCH_2_). Formation and application to organic synthesis. J. Am. Chem. Soc. 1965, 87, 1353–1364. 10.1021/ja01084a034.

[ref33] Ono S.; Yamafuji T.; Chaki H.; Morita H.; Todo Y.; Maekawa M.; Kitamura K.; Tai M.; Narita H. Studies on cognitive enhancing agents. II. Antiamnestic and antihypoxic activities of 1-aryl-2-(2-aminoethoxy)ethanols. Chem. Pharm. Bull. 1995, 43, 1488–1491. 10.1248/cpb.43.1488. 7586071

[ref34] von Hopff H.; Wandeler R. Über schwefelhaltige und andere, aromatisch substituierte mono- und diepoxide. Helv. Chim. Acta 1962, 45, 992–996. 10.1002/hlca.19620450326.

[ref35] Virgili A.; Cervelló J. Composition of iodopropylideneglycerol: an example of the application of 2D NMR spectroscopy to structure elucidation. Magn. Reson. Chem. 1996, 34, 434–439. 10.1002/(sici)1097-458x(199606)34:6<434::aid-omr897>3.0.co;2-o.

[ref36] Take K.; Okumura K.; Tsubaki K.; Terai T.; Shiokawa Y. Agents for the treatment of overactive detrusor. III. Synthesis and structure-activity relationships of N-(4-amino-2-butynyl)acetamide derivatives. Chem. Pharm. Bull. 1992, 40, 1415–1423. 10.1248/cpb.40.1415. 1394662

[ref37] Micheel F.; Schleifstein Z.-H. Synthese von Isochroman-Derivaten aus DL-Glycerinaldehyd und Benzol in flüssigem Fluorwasserstoff. Chem. Ber. 1972, 105, 650–657. 10.1002/cber.19721050231. 4645600

[ref38] Aubé J.; Mossman C. J.; Dickey S. (2S, 3S, 5S)- and (2S, 3S, 5R)-5-carboxaldehyde-2,3-diphenyl-1,4-dioxane as surrogates for optically pure 2,3-O-isopropyiideneglyceraldehyde in asymmetric synthesis. Tetrahedron 1992, 48, 9819–9826. 10.1016/s0040-4020(01)92276-8.

[ref39] Jha S. C.; Joshi N. N. Intramolecular dehydrohalogenation during base-mediated reaction of diols with dihaloalkanes. J. Org. Chem. 2002, 67, 3897–3899. 10.1021/jo011046t. 12027709

[ref40] Wildemann H.; Dünkelmann P.; Müller M.; Schmidt B. A short olefin metathesis-based route to enantiomerically pure arylated dihydropyrans and α,β-unsaturated δ-valero lactones. J. Org. Chem. 2003, 68, 799–804. 10.1021/jo0264729. 12558401

[ref41] Smith H. E. Chiroptical properties of the benzene chromophore. A method for the determination of the absolute configurations of benzene compounds by application of the benzene sector and benzene chirality rules. Chem. Rev. 1998, 98, 1709–1740. 10.1021/cr9410124. 11848945

[ref42] Snatzke G.; Ho P. C. Circular dichroism. XLVI. Rules for benzene Cotton-effects. Tetrahedron 1971, 27, 3645–3653. 10.1016/s0040-4020(01)97775-0.

[ref43] Pescitelli G.; Di Bari L.; Caporusso A. M.; Salvadori P. The prediction of the circular dichroism of the benzene chromophore: TDDFT calculations and sector rules. Chirality 2008, 20, 393–399. 10.1002/chir.20460. 17724654

[ref44] Grimme S.; Parac M. Substantial Errors from Time-Dependent Density Functional Theory for the Calculation of Excited States of Large π Systems. ChemPhysChem 2003, 4, 292–295. 10.1002/cphc.200390047. 12674603

[ref45] Pescitelli G.; Barone V.; Di Bari L.; Rizzo A.; Santoro F. Vibronic coupling dominates the electronic circular dichroism of the benzene chromophore 1Lb band. J. Org. Chem. 2013, 78, 7398–7405. 10.1021/jo401112v. 23834013

[ref46] Pescitelli G.; Bruhn T. Good computational practice in the assignment of absolute configurations by TDDFT calculations of ECD spectra. Chirality 2016, 28, 466–474. 10.1002/chir.22600. 27098594

[ref47] Górecki M.; Zullo V.; Iuliano A.; Pescitelli G. On the absolute stereochemistry of Tolterodine: a circular dichroism study. Pharmaceuticals 2019, 12, 21 10.3390/ph12010021. PMC6469158 30691175

[ref48] Del Bello F.; Bonifazi A.; Giorgioni G.; Cifani C.; Micioni Di Bonaventura M. V.; Petrelli R.; Piergentili A.; Fontana S.; Mammoli V.; Yano H.; Matucci R.; Vistoli G.; Quaglia W. 1-[3-(4-Butylpiperidin-1-yl)propyl]-1,2,3,4-tetrahydroquinolin-2-one (77-LH-28-1) as a model for the rational design of a novel class of brain penetrant ligands with high affinity and selectivity for dopamine D_4_ receptor. J. Med. Chem. 2018, 61, 3712–3725. 10.1021/acs.jmedchem.8b00265. 29589445

[ref49] Bonifazi A.; Yano H.; Del Bello F.; Farande A.; Quaglia W.; Petrelli R.; Matucci R.; Nesi M.; Vistoli G.; Ferré S.; Piergentili A. Synthesis and biological evaluation of a novel series of heterobivalent muscarinic ligands based on xanomeline and 1-[3-(4-butylpiperidin-1-yl)propyl]-1,2,3,4-tetrahydroquinolin-2-one (77-LH-28-1). J. Med. Chem. 2014, 57, 9065–9077. 10.1021/jm501173q. 25275964

[ref50] Mansfield K. J.; Chandran J. J.; Vaux K. J.; Millard R. J.; Christopoulos A.; Mitchelson F. J.; Burcher E. Comparison of receptor binding characteristics of commonly used muscarinic antagonists in human bladder detrusor and mucosa. J. Pharmacol. Exp. Ther. 2009, 328, 893–899. 10.1124/jpet.108.145508. 19029429

[ref51] Boyle C. D.; Lachowicz J. E. Orally active and selective benzylidene ketal M_2_ muscarinic receptor antagonists for the treatment of Alzheimer’s disease. Drug Dev. Res. 2002, 56, 310–320. 10.1002/ddr.10084.

[ref52] Liu H.; Hofmann J.; Fish I.; Schaake B.; Eitel K.; Bartuschat A.; Kaindl J.; Rampp H.; Banerjee A.; Hübner H.; Clark M. J.; Vincent S. G.; Fisher J. T.; Heinrich M. R.; Hirata K.; Liu X.; Sunahara R. K.; Shoichet B. K.; Kobilka B. K.; Gmeiner P. Structure-guided development of selective M3 muscarinic acetylcholine receptor antagonists. Proc. Natl. Acad. Sci. U.S.A. 2018, 115, 12046–12050. 10.1073/pnas.1813988115. 30404914 PMC6255194

[ref53] Agas D.; Marchetti L.; Douni E.; Sabbieti M. G. The unbearable lightness of bone marrow homeostasis. Cytokine Growth Factor Rev. 2015, 26, 347–359. 10.1016/j.cytogfr.2014.12.004. 25563564

[ref54] Daina A.; Michielin O.; Zoete V. SwissADME: a free web tool to evaluate pharmacokinetics, drug-likeness and medicinal chemistry friendliness of small molecules. Sci. Rep. 2017, 7, 42717 10.1038/srep42717. 28256516 PMC5335600

[ref55] Pedretti A.; Mazzolari A.; Vistoli G.; Testa B. MetaQSAR: an integrated database engine to manage and analyze metabolic data. J. Med. Chem. 2018, 61, 1019–1030. 10.1021/acs.jmedchem.7b01473. 29244953

[ref56] Frisch M. J.; Trucks G. W.; Schlegel H. B.; Scuseria G. E.; Robb M. A.; Cheeseman J. R.; Scalmani G.; Barone V.; Mennucci B.; Petersson G. A.; Nakatsuji H.; Caricato M.; Li X.; Hratchian H. P.; Izmaylov A. F.; Bloino J.; Zheng G.; Sonnemberg J. L.; Hada M.; Ehara M.; Toyota K.; Fukuda R.; Hasegawa J.; Ishida M.; Nakajima T.; Honda Y.; Kitao O.; Nakai H.; Vreven T.; Montgomery J. A. Jr.; Peralta J. E.; Ogliaro F.; Bearpark M.; Heyd J. J.; Brothers E.; Kudin K. N.; Staroverov V. N.; Kobayashi R.; Normand J.; Raghavachari K.; Rendell A.; Burant J. C.; Iyengar S. S.; Tomasi J.; Cossi M.; Rega N.; Millam J. M.; Klene M.; Knox J. E.; Cross J. B.; Bakken V.; Adamo C.; Jaramillo J.; Gomperts R.; Stratmann R. E.; Yazyev O.; Austin A. J.; Cammi R.; Pomelli C.; Ochterski J. W.; Martin R. L.; Morokuma K.; Zakrzewski V. G.; Voth G. A.; Salvador P.; Dannenberg J. J.; Dapprich S.; Daniels A. D.; Farkas O.; Foresman J. B.; Ortiz J. V.; Cioslowski J.; Fox D. J.; Gaussian 09, Revision A.02; Gaussian, Inc.: Wallingford CT, 2016.

[ref57] Bruhn T.; Schaumlöffel A.; Hemberger Y.; Pescitelli G. SpecDis, version 1.71, Berlin, Germany, 2017, http:/specdis-software.jimdo.com.

[ref58] Bruhn T.; Schaumlöffel A.; Hemberger Y.; Bringmann G. SpecDis: Quantifying the comparison of calculated and experimental electronic circular dichroism spectra. Chirality 2013, 25, 243–249. 10.1002/chir.22138. 23532998

[ref59] Cheng Y.-C.; Prusoff W. H. Relationship between the inhibition constant (K_i_) and the concentration of inhibitor which causes 50 percent inhibition (I_50_) of an enzymatic reaction. Biochem. Pharmacol. 1973, 22, 3099–3108. 10.1016/0006-2952(73)90196-2. 4202581

[ref60] Pedretti A.; Villa L.; Vistoli G. VEGA: a versatile program to convert, handle and visualize molecular structure on Windows-based PCs. J. Mol. Graphics Modell. 2002, 21, 47–49. 10.1016/s1093-3263(02)00123-7. 12413030

[ref61] Phillips J. C.; Braun R.; Wang W.; Gumbart J.; Tajkhorshid E.; Villa E.; Chipot C.; Skeel R. D.; Kalé L.; Schulten K. Scalable molecular dynamics with NAMD. J. Comput. Chem. 2005, 26, 1781–1802. 10.1002/jcc.20289. 16222654 PMC2486339

[ref62] Stewart J. J. P. Optimization of parameters for semiempirical methods VI: more modifications to the NDDO approximations and re-optimization of parameters. J. Mol. Model. 2013, 19, 1–32. 10.1007/s00894-012-1667-x. 23187683 PMC3536963

[ref63] Korb O.; Stützle T.; Exner T. E. Empirical Scoring Functions for Advanced Protein–Ligand Docking with PLANTS. J. Chem. Inf. Model. 2009, 49, 84–96. 10.1021/ci800298z. 19125657

[ref64] Vistoli G.; Mazzolari A.; Testa B.; Pedretti A. Binding space concept: a new approach to enhance the reliability of docking scores and its application to predicting butyrylcholinesterase hydrolytic activity. J. Chem. Inf. Model. 2017, 57, 1691–1702. 10.1021/acs.jcim.7b00121. 28633528

[ref65] Soleimani M.; Nadri S. A protocol for isolation and culture of mesenchymal stem cells from mouse bone marrow. Nat. Protoc. 2009, 4, 102–106. 10.1038/nprot.2008.221. 19131962

