# Supplementary material for: Novel Potent Muscarinic Receptor Antagonists: Investigation on the Nature of Lipophilic Substituents in the 5- and/or 6-Positions of the 1,4-Dioxane Nucleus
Source: J Med Chem. 2020 May 6;63(11):5763–82. doi: 10.1021/acs.jmedchem.9b02100 (PMC8007111; doi:10.1021/acs.jmedchem.9b02100)
Supplement: Supplementary file 1 — jm9b02100_si_001.pdf [file jm9b02100_si_001.pdf]

Supporting Information

Novel potent muscarinic receptor antagonists: investigation on the nature of lipophilic substituents in the 5- and/or 6-positions of the 1,4-dioxane nucleus

Fabio Del Bello, Alessandro Bonifazi, Gianfabio Giorgioni, Alessandro Piergentili*, Maria Giovanna Sabbieti, Dimitrios Agas, Marzia Dell'Aera, Rosanna Matucci, Marcin Górecki, Gennaro Pescitelli, Giulio Vistoli, Wilma Quaglia

Table of Contents:

-Figure 1S: ^{1}H NMR and ^{13}C NMR spectra of **3a**.

-Figure 2S: ^{1}H NMR and ^{13}C NMR spectra of **3b**.

-Figure 3S: ^{1}H NMR and ^{13}C NMR spectra of the oxalate salt of **33b**.

-Figure 4S: ^{1}H NMR spectra of **28a** and **28b**.

-Figure 5S: ^{1}H NMR spectra of **52a** and **52b**.

-Figure 6S: ^{1}H NMR spectra of **75a** and **75b**.

-Figure 7S: ^{1}H NMR and NOESY spectra of **11a**.

-Figure 8S: ^{1}H NMR and NOESY spectra of **18c**.

-Figure 9S: HPLC chromatograms of (±)-**3b** and its enantiomers.

-Table 1S: elemental analysis results for compounds **3-19**, **33b** and enantiomers of **3b** and **33b**.

-Experimental procedures for the synthesis of intermediates **22**, **24-26**, **28-32**, **40-44**, **46**, **47**, **50-56**, **67**, **68c**, **70**, **72-74**.

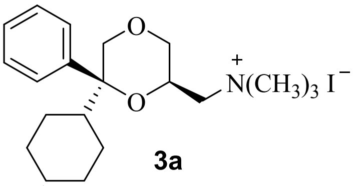

3a

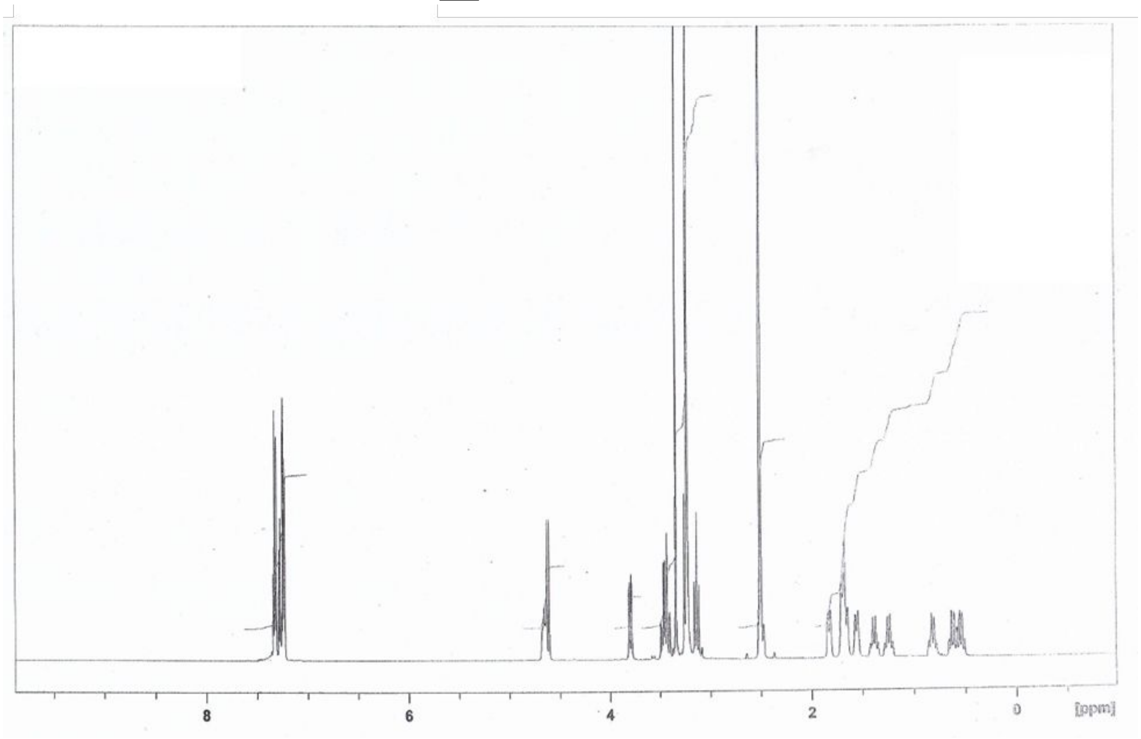

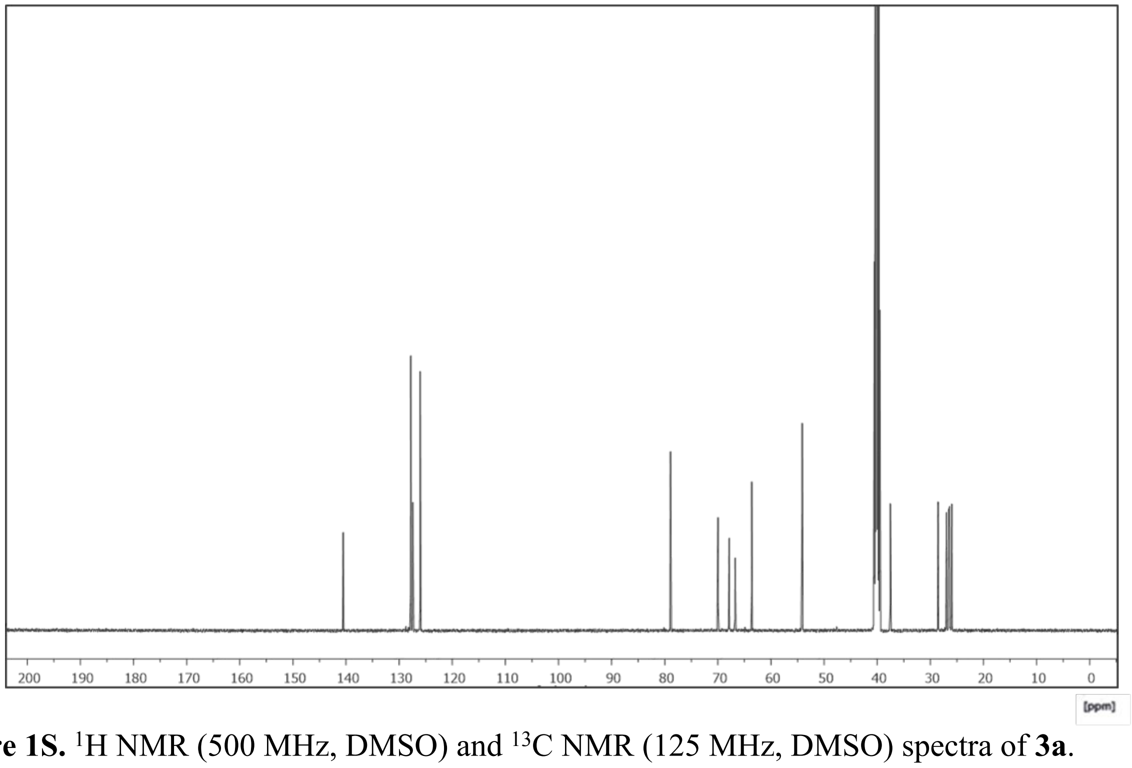

Figure 1S. ^{1}H NMR (500 MHz, DMSO) and ^{13}C NMR (125 MHz, DMSO) spectra of **3a**.

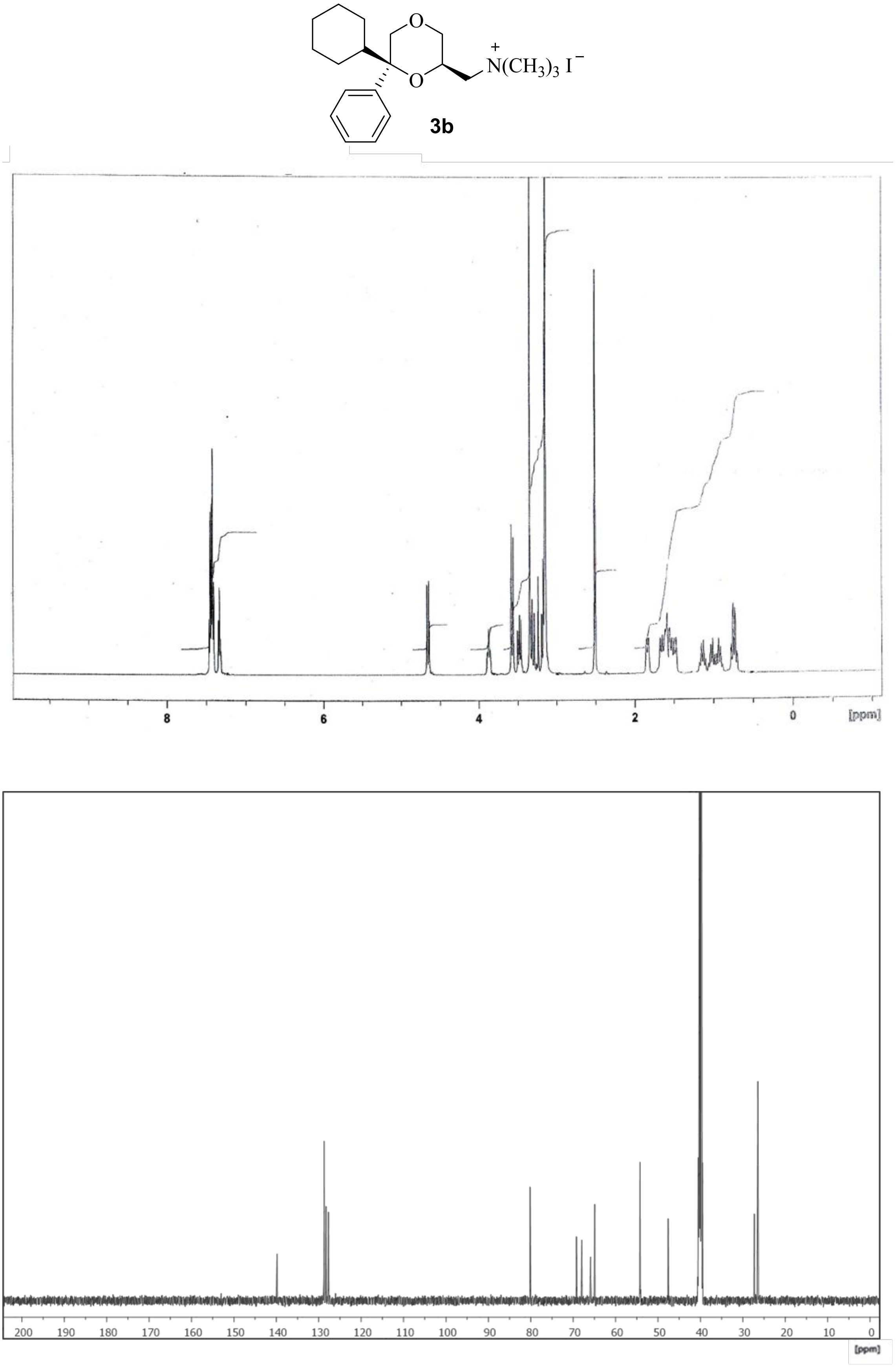

Figure 2S. ^{1}H NMR (500 MHz, DMSO) and ^{13}C NMR (125 MHz, DMSO) spectra of **3b**.

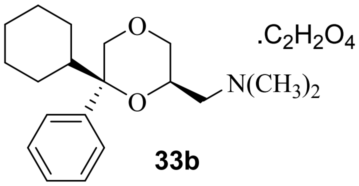

33b

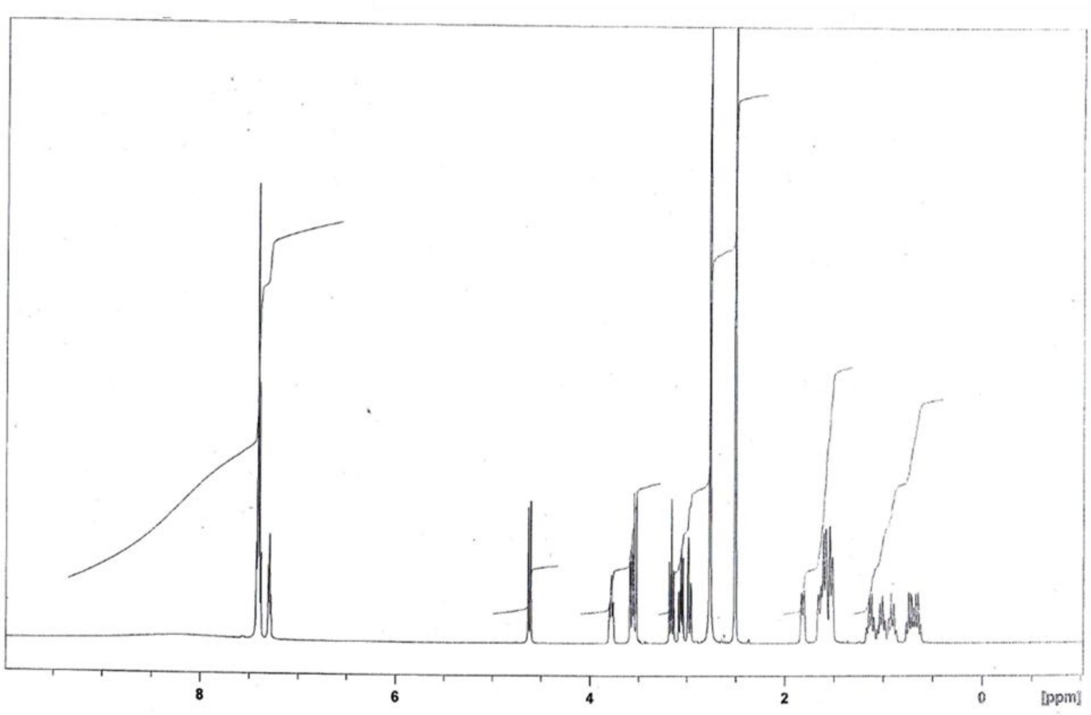

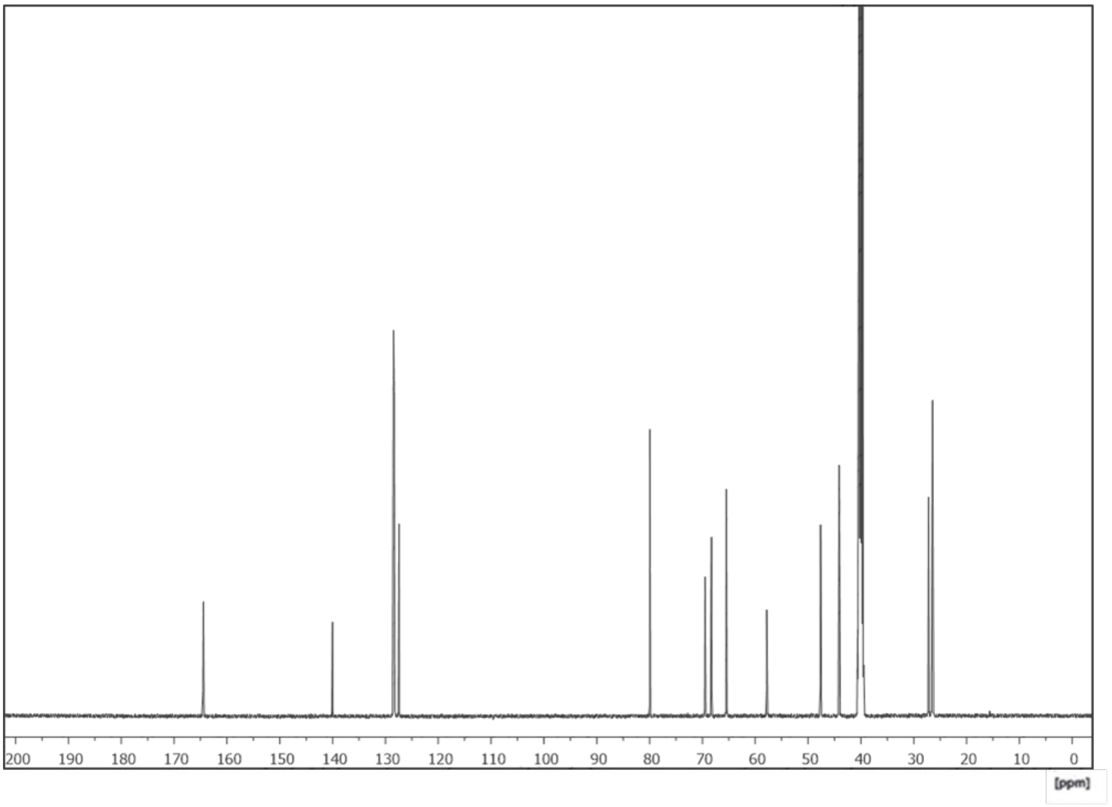

Figure 3S. ^{1}H NMR (500 MHz, DMSO) and ^{13}C NMR (125 MHz, DMSO) spectra of the oxalate salt of **33b**.

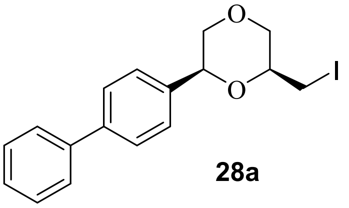

28a

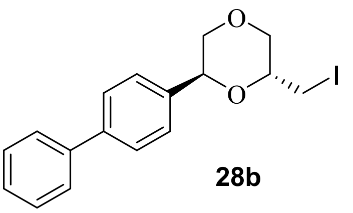

28b

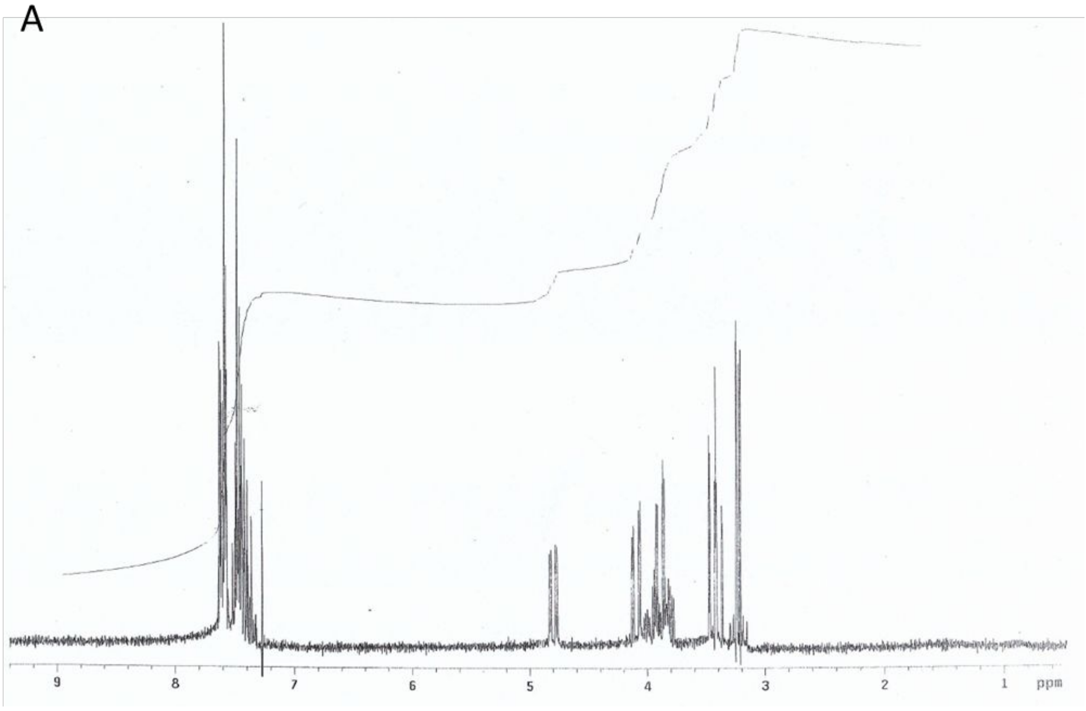

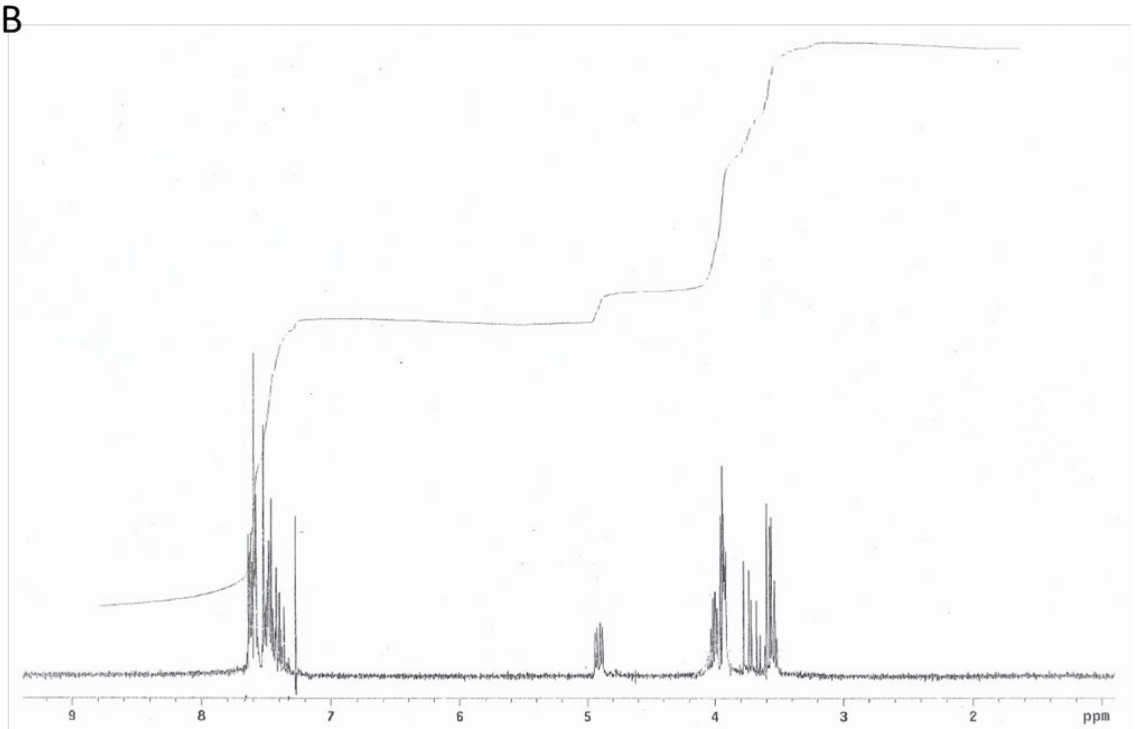

Figure 4S. ^{1}H NMR spectra (200 MHz, $CDCl_3$) of **28a** (A) and **28b** (B).

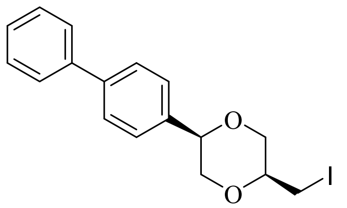

52a

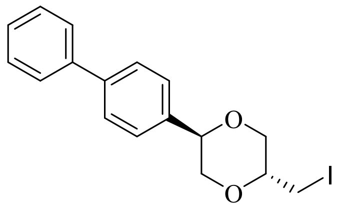

52b

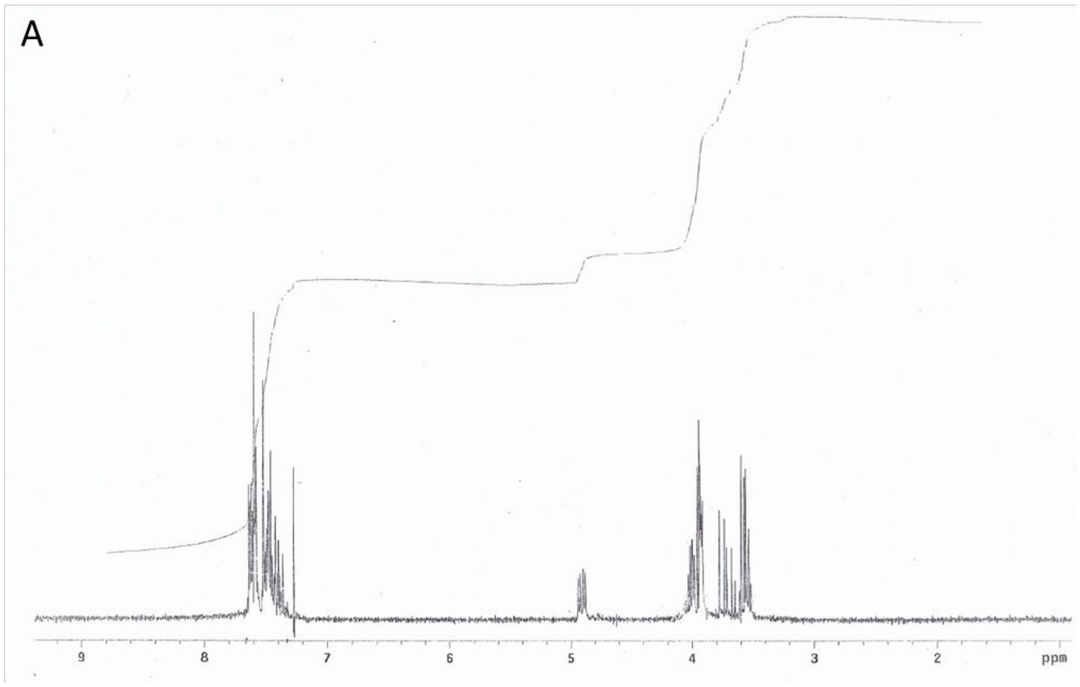

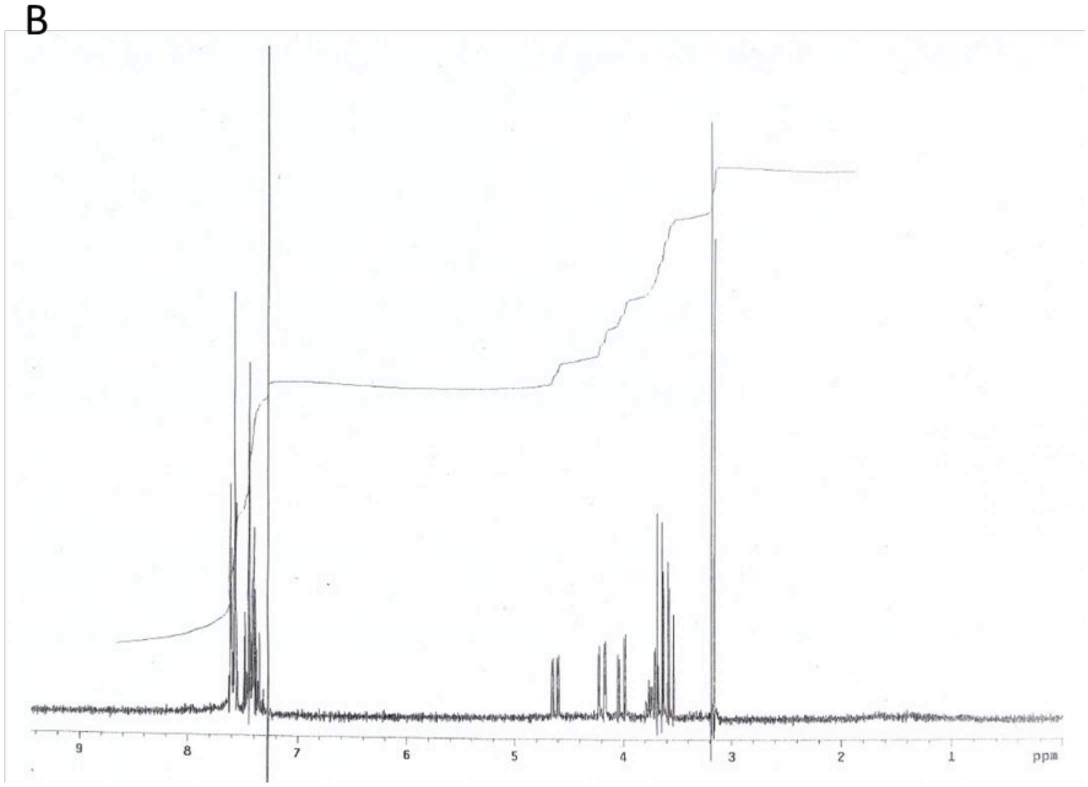

Figure 5S. ^{1}H NMR spectra (200 MHz, $CDCl_3$) of **52a** (A) and **52b** (B).

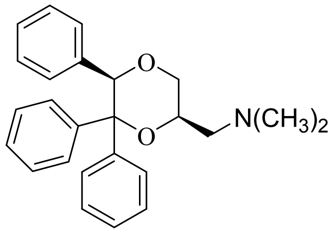

75a

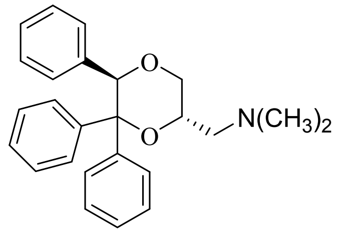

75b

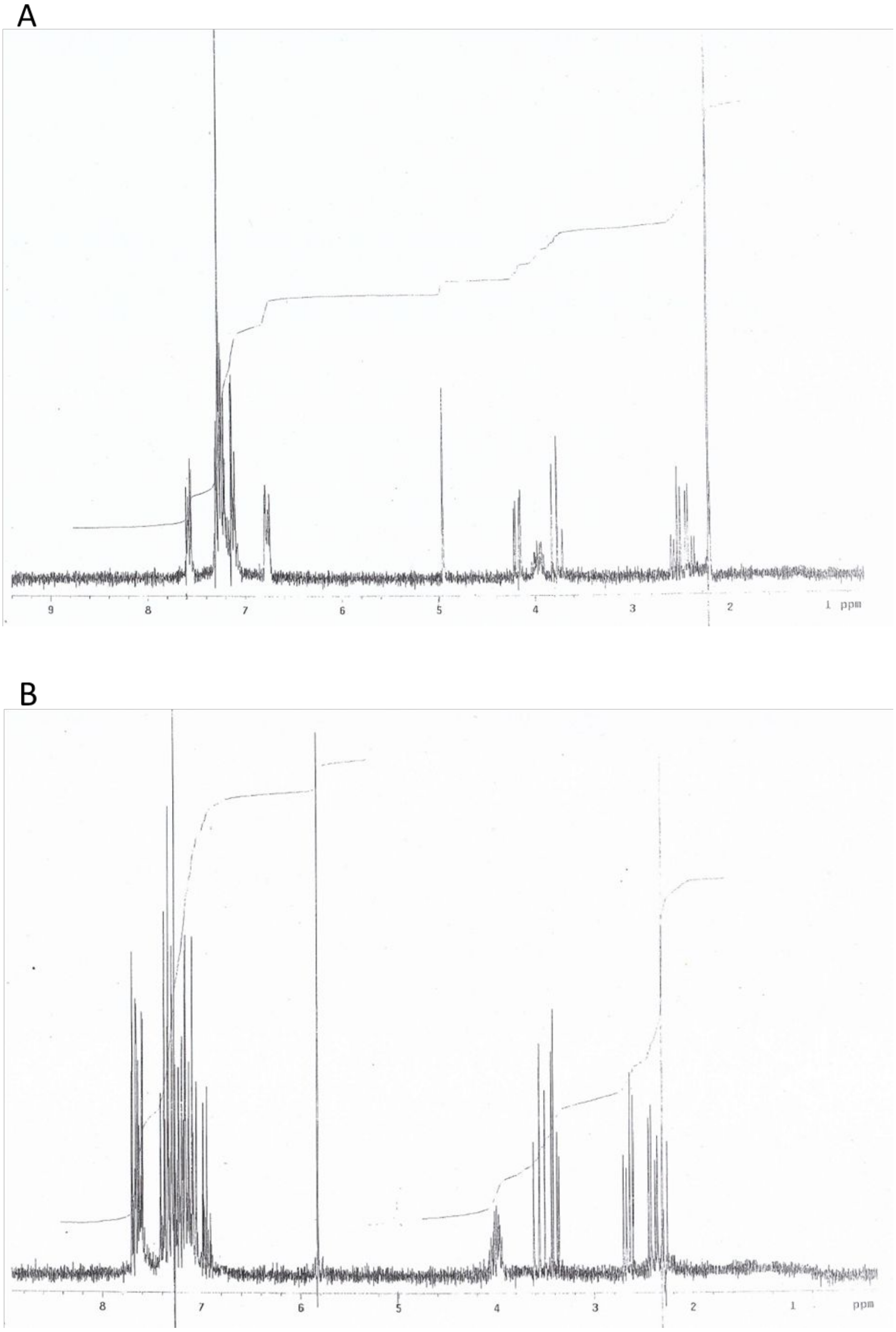

Figure 6S. ^{1}H NMR spectra (200 MHz, $CDCl_3$) of **75a** (A) and **75b** (B).

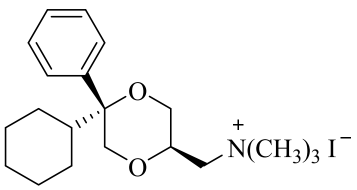

11a

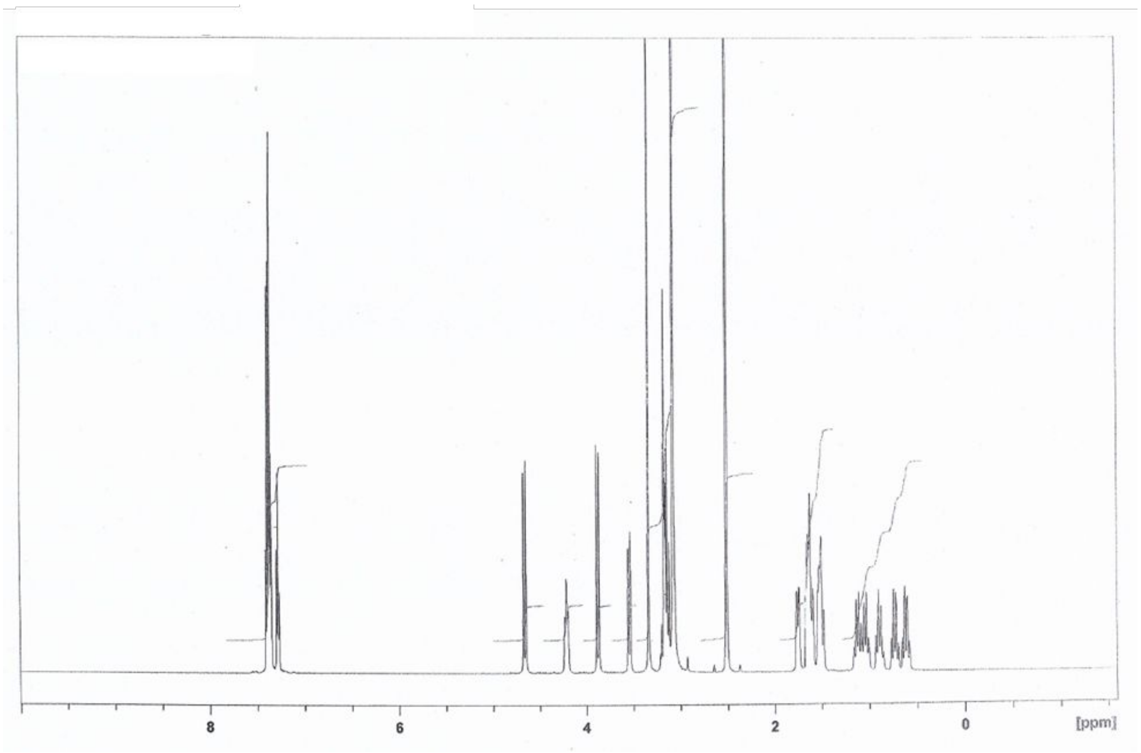

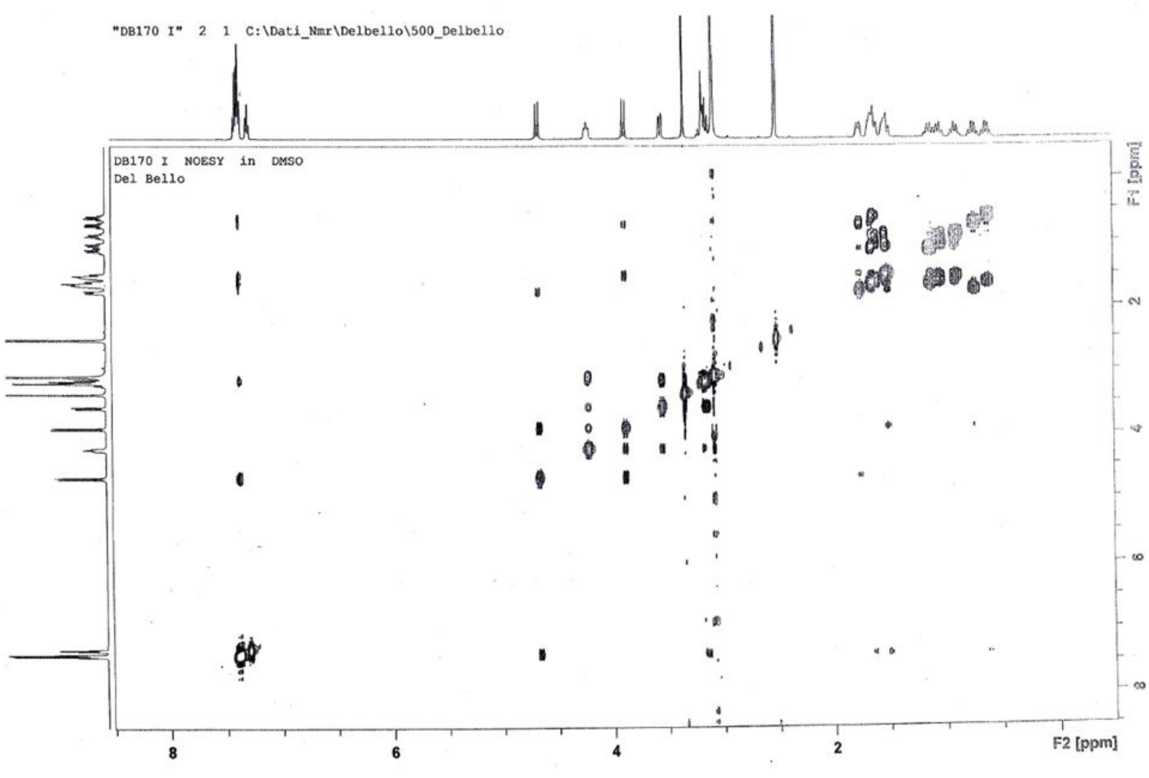

Figure 7S. ^{1}H NMR and NOESY spectra (500 MHz, DMSO) of **11a**.

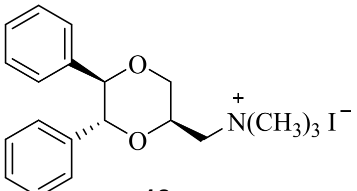

18c

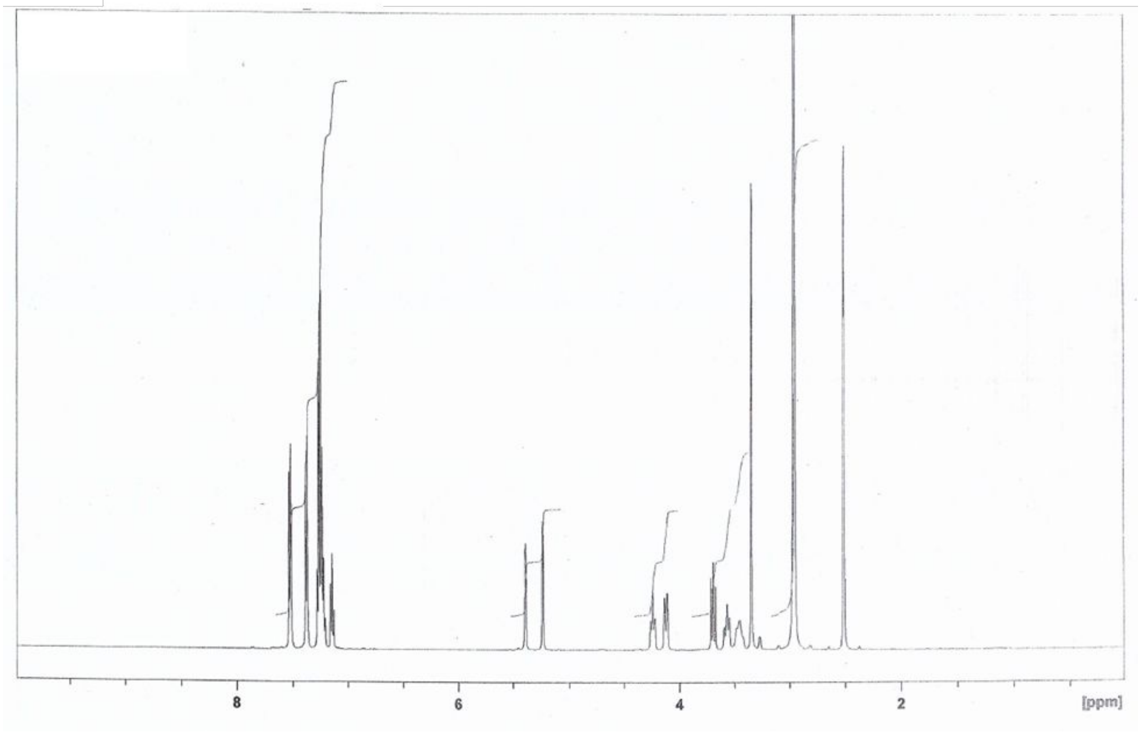

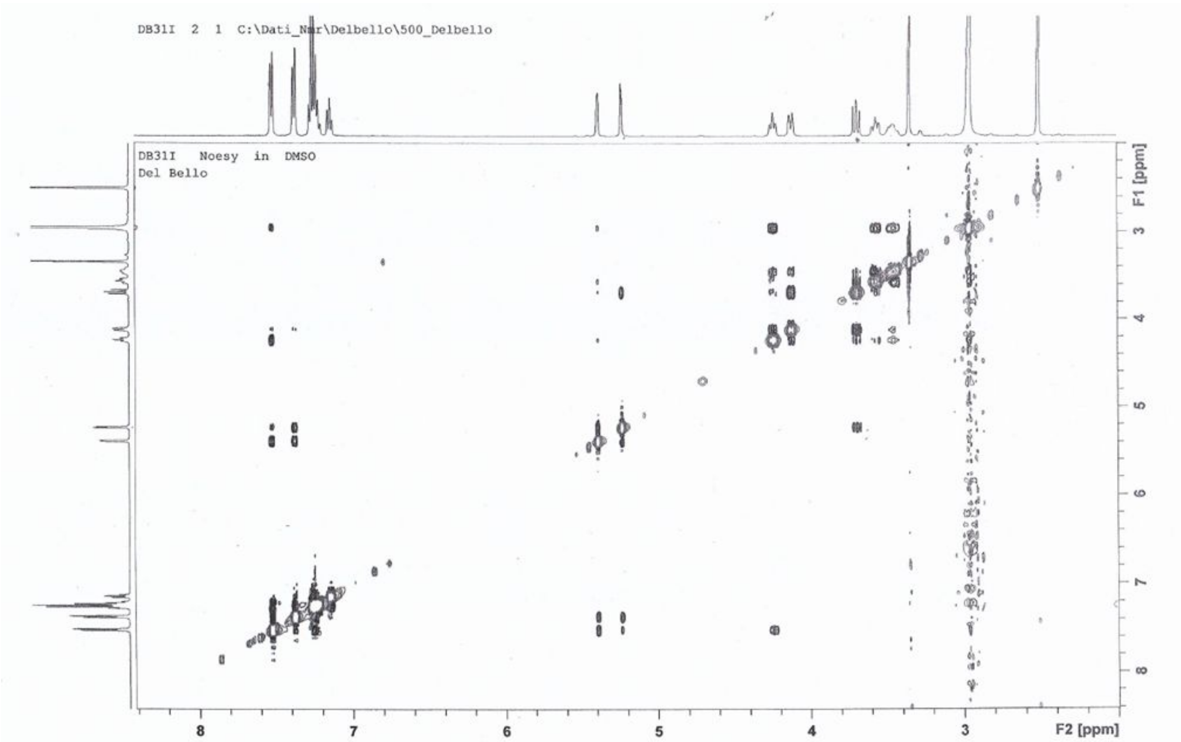

Figure 8S. ^{1}H NMR and NOESY spectra (500 MHz, DMSO) of **18c**.

Analytical chiral HPLC conditions and results:

Column	Whelk O1 (R,R) (25 x 0.46 cm), 10 um
Mobile phase	n-Hexane/2-Propanol 85/15 % v/v
Flow rate (ml/min)	1.0
DAD	220 nm
Loop	20 µL
**Enantiomer 1**	50 % a/a (4.7 min)
**Enantiomer 2**	50 % a/a (10.6 min)

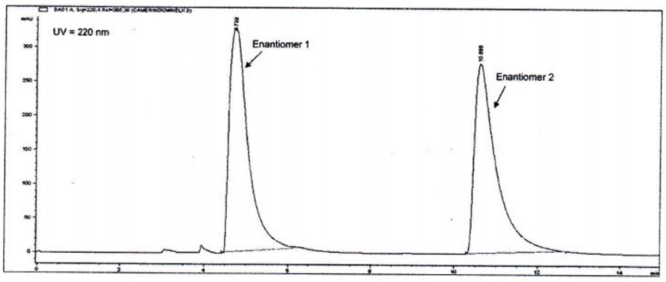

A

Analytical chiral HPLC conditions and results:

Column	Whelk O1 (R,R) (25 x 0.46 cm), 10 um
Mobile phase	n-Hexane/2-Propanol 85/15 % v/v
Flow rate (ml/min)	1.0
DAD	220 nm
Loop	20 µL
**Enantiomer 1**	100 % e.e. (5.6 min)

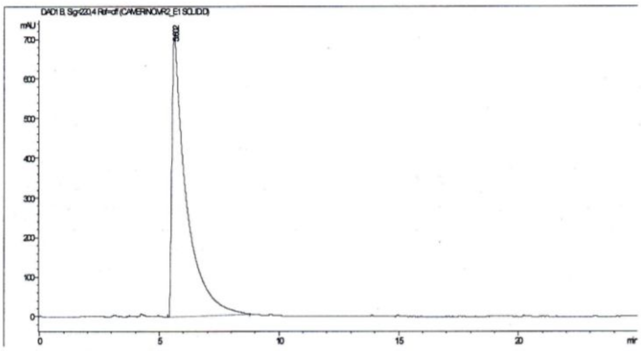

B

Analytical chiral HPLC conditions and results:

Column	Whelk O1 (R,R) (25 x 0.46 cm), 10 um
Mobile phase	n-Hexane/2-Propanol 85/15 % v/v
Flow rate (ml/min)	1.0
DAD	220 nm
Loop	20 µL
**Enantiomer 2**	100 % e.e. (11.4 min)

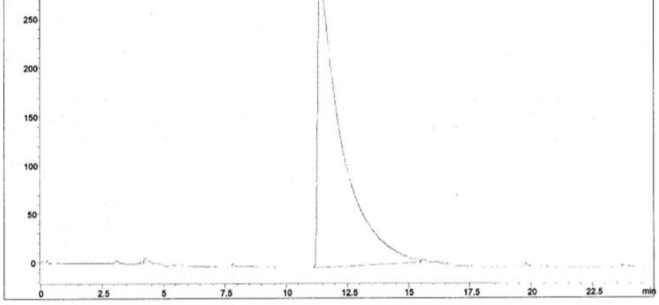

C

Figure 9S. HPLC chromatograms of (±)-**3b** (A) and its enantiomers (2*S*,6*S*)-(−)-**3b** (B) and (2*R*,6*R*)-(+)-**3b** (C).

Table 1S. Elemental analysis results for compounds **3-19**, **33b** and enantiomers of **3b** and **33b**

Compd	Formula	Calcd				Found			
		C%	H%	N%	S%	C%	H%	N%	S%
**3a**	$C_{20}H_{32}INO_2$	53.94	7.24	3.14	-	53.82	7.39	3.10	-
**3b**	$C_{20}H_{32}INO_2$	53.94	7.24	3.14	-	53.78	7.35	3.00	-
(2*S*,6*S*)-(−)-**3b**	$C_{20}H_{32}INO_2$	53.94	7.24	3.14	-	53.77	7.40	3.09	-
(2*R*,6*R*)-(+)-**3b**	$C_{20}H_{32}INO_2$	53.94	7.24	3.14	-	54.03	7.35	2.99	-
**4a**	$C_{20}H_{26}INO_2$	54.68	5.97	3.19	-	54.44	6.11	3.30	-
**4b**	$C_{20}H_{26}INO_2$	54.68	5.97	3.19	-	54.90	6.12	3.06	-
**5a/5b**	$C_{21}H_{28}INO_2$	55.64	6.23	3.09	-	55.41	6.05	2.99	-
**6a**	$C_{20}H_{26}INO_2S$	50.96	5.56	2.97	6.80	60.27	5.54	2.87	6.49
**6b**	$C_{20}H_{26}INO_2S$	50.96	5.56	2.97	6.80	50.83	5.38	2.84	6.98
**7a**	$C_{20}H_{26}INO_3S$	49.29	5.38	2.87	6.58	49.39	5.30	2.96	6.41
**7b**	$C_{20}H_{26}INO_3S$	49.29	5.38	2.87	6.58	49.50	5.17	2.99	6.36
**8a**	$C_{20}H_{26}INO_4S$	47.72	5.21	2.78	6.37	47.90	5.13	2.84	6.57
**8b**	$C_{20}H_{26}INO_4S$	47.72	5.21	2.78	6.37	47.61	5.02	2.68	6.21
**9a**	$C_{14}H_{22}INO_2$	46.29	6.11	3.86	-	46.07	6.00	4.00	-
**9b**	$C_{14}H_{22}INO_2$	46.29	6.11	3.86	-	46.18	6.24	3.92	-
**10**	$C_{20}H_{26}INO_2$	54.68	5.97	3.19	-	54.39	6.10	3.18	-
**11a**	$C_{20}H_{32}INO_2$	53.94	7.24	3.14	-	54.10	7.29	3.02	-
**11b**	$C_{20}H_{32}INO_2$	53.94	7.24	3.14	-	54.03	7.37	3.08	-
**12a**	$C_{21}H_{28}INO_2$	55.64	6.23	3.09	-	55.42	6.30	3.21	-
**12b**	$C_{21}H_{28}INO_2$	55.64	6.23	3.09	-	55.53	6.12	3.19	-
**13a**	$C_{20}H_{26}INO_2$	54.68	5.97	3.19	-	54.50	6.13	3.22	-
**13b**	$C_{20}H_{26}INO_2$	54.68	5.97	3.19	-	54.79	6.08	3.34	-
**14a**	$C_{21}H_{28}INO_2$	55.64	6.23	3.09	-	55.79	6.34	2.96	-
**14b**	$C_{21}H_{28}INO_2$	55.64	6.23	3.09	-	55.87	6.04	3.01	-
**15a**	$C_{20}H_{26}INO_2S$	50.96	5.56	2.97	6.80	51.14	5.71	3.10	6.97
**15b**	$C_{20}H_{26}INO_2S$	50.96	5.56	2.97	6.80	50.83	5.69	2.86	6.74
**16a**	$C_{20}H_{26}INO_3S$	49.29	5.38	2.87	6.58	48.99	5.44	2.94	6.43
**16b**	$C_{20}H_{26}INO_3S$	49.29	5.38	2.87	6.58	49.18	5.18	3.01	6.40
**17a**	$C_{20}H_{26}INO_4S$	47.72	5.21	2.78	6.37	47.94	5.36	2.70	6.24
**17b**	$C_{20}H_{26}INO_4S$	47.72	5.21	2.78	6.37	47.88	5.36	2.67	6.19
**18a**	$C_{20}H_{26}INO_2$	54.68	5.97	3.19	-	54.85	6.12	3.23	-
**18b**	$C_{20}H_{26}INO_2$	54.68	5.97	3.19	-	54.40	5.99	3.12	-
**18c**	$C_{20}H_{26}INO_2$	54.68	5.97	3.19	-	54.53	6.08	3.07	-
**19a**	$C_{26}H_{30}INO_2$	60.59	5.87	2.72	-	60.89	5.95	2.70	-
**19b**	$C_{26}H_{30}INO_2$	60.59	5.87	2.72	-	60.74	6.00	2.84	-
**33b**	$C_{19}H_{29}NO_2{\cdot}C_2H_2O_4$	64.10	7.94	3.56	-	63.94	8.12	3.40	-
(2*S*,6*S*)-(−)-**33b**	$C_{19}H_{29}NO_2{\cdot}C_2H_2O_4$	64.10	7.94	3.56	-	63.81	8.01	3.44	-
(2*R*,6*R*)-(+)-**33b**	$C_{19}H_{29}NO_2{\cdot}C_2H_2O_4$	64.10	7.94	3.56	-	64.29	8.15	3.68	-

Experimental procedures for the synthesis of intermediates 22, 24-26, 28-32, 40-44, 46, 47, 50-56, 67, 68c, 70, 72-74.

2-(4-Benzylphenyl)oxirane (22)

NaH (0.31 g, 12.9 mmol) was added to a solution of 4-benzylbenzaldehyde (**20**)[31] (1.00 g, 5.1 mmol) and $(CH_3)_3SI$ (1.1 g, 5.4 mmol) in DMSO. The mixture was stirred at room temperature for 24 h and the reaction was then quenched with brine. The aqueous layer was extracted with diethyl ether (2 × 50 mL). The combined organic phase was dried over anhydrous Na_2SO_4. Removal of the dried solvent gave **22** as an oil (71% yield). ^{1}H NMR ($CDCl_3$): δ 2.81 (dd, J = 2.7, 5.5 Hz, 1H, CH_2O), 3.16 (dd, J = 4.1, 5.5 Hz, 1H, CH_2O), 3.86 (dd, J = 2.7, 4.1 Hz, 1H, CHO), 4.00 (s, 2H, CH_2Ar), 7.17-7.39 (m, 9H, ArH).

1-([1,1'-Biphenyl]-4-yl)-2-(allyloxy)ethan-1-ol (24)

2-([1,1'-biphenyl]-4-yl)oxirane (**21**)[33] (2.23 g, 11.3 mmol) was added portionwise to a stirred solution of freshly cut sodium (0.08 g, 3.6 mmol) in 2-methyl allyl alcohol (8 mL) at r.t.. After 1 h the reaction mixture was refluxed for 20 h. After cooling to room temperature, 6 N H_2SO_4 (0.6 mL) was added to the residual solution to neutralize the sodium alloxide, and solvent removal was continued to afford a residual oil, which was purified by column chromatography eluting with cyclohexane/EtOAc (9:1). An oil was obtained (60% yield). ^{1}H NMR ($CDCl_3$): δ 2.61 (br s, 1H, OH), 3.52 (dd, J = 9.79, 9.07 Hz, 1H, OCH_2), 3.69 (dd, J = 9.79, 3.17 Hz, 1H, OCH_2), 4.12 (m, 2H, OCH_2), 4.98 (dd, J = 9.06, 3.16 Hz, 1H, OCH), 5.31 (m, 2H, $CH{=}CH_2$), 5.98 (m, 1H, $CH{=}CH_2$), 7.31-7.62 (m, 9H, ArH).

2-(Allyloxy)-1-(4-benzylphenyl)ethan-1-ol (25)

This compound was prepared starting from **22** following the procedure described for **24**: an oil was obtained (65% yield). ^{1}H NMR ($CDCl_3$): δ ^{1}H NMR ($CDCl_3$): δ 2.82 (br s, 1H, OH), 3.48 (dd, J = 9.80, 9.07 1H, CH_2), 3.62 (dd, J = 9.80, 3.15 Hz 1H, CH_2), 3.99 (s, 2H, $ArCH_2$), 4.11 (m, 2H,

OCH_2), 4.90 (dd, *J* = 9.07, 3.16 Hz, 1H, OCH), 5.24 (m, 2H, $CH{=}CH_2$), 5.94 (m, 1H, $CH{=}CH_2$), 7.15-7.38 (m, 9H, ArH).

*2-(Allyloxy)-1-(4-(phenylthio)phenyl)ethan-1-ol (**26**)*

This compound was prepared starting from **23**[34] following the procedure described for **24**: an oil was obtained (52% yield). ^{1}H NMR ($CDCl_3$): δ ^{1}H NMR ($CDCl_3$): δ 2.42 (br s, 1H, OH), 3.42 (dd, *J* = 9.80, 9.08 Hz, 1H, OCH_2), 3.60 (dd, *J* = 9.80, 3.17 Hz, 1H, OCH_2), 4.07 (m, 2H, OCH_2), 4.89 (dd, *J* = 9.08, 3.16 Hz, 1H, OCH), 5.25 (m, 2H, $CH{=}CH_2$), 5.92 (m, 1H, $CH{=}CH_2$), 7.20-7.38 (m, 9H, ArH).

(2S,6S*)-2-([1,1'-Biphenyl]-4-yl)-6-(iodomethyl)-1,4-dioxane (**28a**) and (2R*,6S*)-2-([1,1'-Biphenyl]-4-yl)-6-(iodomethyl)-1,4-dioxane (**28b**)*

A solution of mercury(II) acetate (5.28 g, 16.6 mmol) in H_2O (25 mL) and acetic acid (0.025 mL) was added to a stirred solution of **24** (4.00 g, 15.7 mmol). The reaction mixture was heated to reflux for 45 min, then allowed to stand overnight at room temperature. After the reaction mixture was filtered, a solution of KI (2.88 g, 17.4 mmol) in H_2O (25 mL) was added to the filtrate and ((5-phenyl-1,4-dioxan-2-yl)methyl)mercury(II) iodide separated as an oil, which was dissolved in $CHCl_3$ (16 mL). A solution of I_2 (3.20 g, 12.6 mmol) in $CHCl_3$ was added, and the reaction mixture was heated to boiling and then allowed to stand at room temperature for 18 h. The organic phase was washed with 10% Na_2SO_3 and 10% KI and dried over Na_2SO_4. The evaporation of the solvent in vacuo afforded a mixture of the two diastereoisomers, which were separated by column chromatography, eluting with cyclohexane/EtOAc (99:1). The *trans* isomer **28b** eluted first as a solid: 1.2 g; 20% yield; m.p. 137-139 °C. ^{1}H NMR ($CDCl_3$): δ 3.55 (m, 2H, CH_2I), 3.73 (m, 2H, dioxane), 3.88-4.06 (m, 3H, dioxane), 4.91 (dd, 1H, dioxane), 7.32-7.67 (m, 9H, ArH). The second fraction was the *cis* isomer **28a**: 1.1 g; 18% yield. ^{1}H NMR ($CDCl_3$): δ 3.22 (m, 2H, CH_2I), 3.40 (m,

2H, dioxane), 3.84 (m, 2H, dioxane), 4.09 (dd, 1H, dioxane), 4.79 (dd, 1H, dioxane), 7.32-7.63 (m, 9H, ArH).

(2S,6S*)-2-(4-Benzylphenyl)-6-(iodomethyl)-1,4-dioxane and (2R*,6S*)-2-(4-Benzylphenyl)-6-(iodomethyl)-1,4-dioxane (**29a/b**)*

This mixture of *cis*/*trans* (6:4) diastereomers was prepared starting from **25** following the procedure described for **28**: an oil was obtained (40% yield). ^{1}H NMR ($CDCl_3$): δ 3.18-4.15 (m, 9H *cis* + 9H *trans*, CH_2I, dioxane), 4.70 (dd, 1H *cis*, dioxane), 4.82 (dd, 1H *trans*, dioxane), 7.16-7.38 (m, 9H *cis* + 9H *trans*, ArH).

(2S,6S*)-2-(Iodomethyl)-6-(4-(phenylthio)phenyl)-1,4-dioxane (**30a**) and (2S*,6R*)-2-(Iodomethyl)-6-(4-(phenylthio)phenyl)-1,4-dioxane (**30b**)*

This compound was prepared starting from **26** following the procedure described for **28**. The *trans* isomer **30b** eluted first: 1.4 g; 24% yield. ^{1}H NMR ($CDCl_3$): δ 3.52 (m, 2H, CH_2I), 3.63 (m, 1H, dioxane), 3.79-4.02 (m, 4H, dioxane), 4.82 (dd, 1H, dioxane), 7.28-7.41 (m, 9H, ArH). The second fraction was the *cis* isomer **30a** as a solid m.p. 97-98 °C.: 1.2 g; 20% yield. ^{1}H NMR ($CDCl_3$): δ 3.18 (m, 2H, CH_2I), 3.32 (m, 2H, dioxane), 3.78 (m, 2H, dioxane), 4.02 (dd, 1H, dioxane), 4.71 (dd, 1H, dioxane), 7.20-7.36 (m, 9H, ArH).

(2S,6S*)-2-(Iodomethyl)-6-(4-(phenylsulfinyl)phenyl)-1,4-dioxane (**31a**)*

A solution of **30a** (0.7 g, 1.70 mmol) in CH_2C1_2 was cooled to 0 °C, and *m*-CPBA (0.3 g, 1.70 mmol) was added slowly portionwise. After stirring for 0.5 h at room temperature, the mixture was taken up into ether and washed with a mixture of NaOH, NaI, and $Na_2S_2O_3$. The organic extract was dried over Na_2SO_4, and the solvent was removed. The crude sulfone was purified by column chromatography, eluting with cyclohexane/AcOEt (7:3). An oil was obtained (71% yield). ^{1}H NMR

($CDCl_3$): δ 3.15-3.39 (m, 4H, CH_2I, dioxane), 3.70-3.86 (m, 2H, dioxane), 4.05 (dd, 1H, dioxane), 4.79 (dd, 1H, dioxane), 7.42-7.71 (m, 9H, ArH).

(2S,6R*)-2-(Iodomethyl)-6-(4-(phenylsulfinyl)phenyl)-1,4-dioxane (**31b**)*

This compound was prepared starting from **30b** following the procedure described for **31a**: an oil was obtained (69% yield). ^1H NMR ($CDCl_3$): δ 3.48 (m, 2H, CH_2I), 3.60 (dd, 1H, dioxane), 3.78-4.01 (m, 4H, dioxane), 4.82 (dd, 1H, dioxane), 7.42-7.70 (m, 9H, ArH).

(2S,6S*)-2-(Iodomethyl)-6-(4-(phenylsulfonyl)phenyl)-1,4-dioxane (**32a**)*

A solution of **30a** (0.7 g, 1.70 mmol) in CH_2C1_2 was cooled to 0 °C, and *m*-CPBA (0.6 g, 3.40 mmol) was added slowly portionwise. After stirring for 2 h at room temperature, the mixture was taken up into ether and washed with a mixture of NaOH, NaI, and $Na_2S_2O_3$. The organic layer was dried over Na_2SO_4, and the solvent was removed. The crude product was purified by column chromatography, eluting with cyclohexane/AcOEt (8:2): an oil was obtained (48% yield). ^1H NMR ($CDCl_3$): δ 3.20 (m, 2H, CH_2I), 3.16-3.30 (m, 2H, dioxane), 3.66-3.87 (m, 2H, dioxane), 4.01 (dd, 1H, dioxane), 4.78 (dd, 1H, dioxane), 7.40-7.94 (m, 9H, ArH).

(2S,6R*)-2-(Iodomethyl)-6-(4-(phenylsulfonyl)phenyl)-1,4-dioxane (**32b**)*

This compound was prepared starting from **30b** following the procedure described for **32a**: an oil was obtained (48% yield). ^1H NMR ($CDCl_3$): δ 3.53 (m, 2H, CH_2I), 3.60 (dd, 1H, dioxane), 3.76-4.02 (m, 4H, dioxane), 4.88 (dd, 1H, dioxane), 7.45-8.06 (m, 9, ArH).

*2-(Allyloxy)-2-cyclohexyl-2-phenylethan-1-ol (**40**)*

Perchloric acid (70%, 1 mL) was added to a stirred solution of 2-cyclohexyl-2-phenyloxirane (**39**)[36] (3.9 g, 19.3 mmol) in allyl alcohol (10 mL) at 0 °C. After 0.5 h at room temperature the reaction mixture was poured in H_2O (75 mL) and extracted with Et_2O. The organic phase was washed with

H_2O and dried over Na_2SO_4. Removal of dried solvents gave a residue, which was purified by column chromatography, eluting with cyclohexane/AcOEt (9.8:0.2). An oil was obtained (79% yield). ^1H NMR ($CDCl_3$): δ 0.64-2.01 (m, 12H, cyclohexyl and OH), 3.88 (m, 2H, OCH_2), 4.01 (d, 1H, OCH_2), 4.22 (d, 1H, OCH_2), 5.20 (m, 1H, CH_2=C), 5.41 (m, 1H, CH_2=C), 6.00 (m, 1H, C=CH), 7.20-7.41 (m, 5H, ArH).

*2-(Allyloxy)-3,3-diphenylpropan-1-ol (**41**)*

1 M tetrabutylammonium fluoride (3.7 mL) in THF (4 mL) was added dropwise to a mixture of **47** (1 g, 2.62 mmol) in acetic acid (0.5 mL) for a period of 30 min at 5 °C and the mixture was stirred at room temperature for 6 h. The reaction was poured in $NaHCO_3$ (5 mL) and extracted with Et_2O. The organic phase was dried over Na_2SO_4. Removal of dried solvents gave a residue, which was purified by column chromatography, eluting with cyclohexane/AcOEt (9:1) to afford an oil (82% yield). ^1H NMR ($CDCl_3$): δ 2.92 (br s, 1H, OH), 3.43 (m, 1H, ArCHAr), 3.65-3.97 (m, 3H, CH_2OH, CHO), 4.13 (m, 2H, CH_2C=), 5.08 (m, 2, CH_2=C), 5.72 (m, 1H, CH=C), 7.17-7.41 (m, 10H, ArH).

*2-([1,1'-Biphenyl]-4-yl)-2-(allyloxy)ethan-1-ol (**42**)*

This compound was prepared starting from **21**[33] following the procedure described for **40**: a solid was obtained (62% yield); m.p. 43-45 °C. ^1H NMR ($CDCl_3$): δ 2.32 (m, 1H, OH), 3.62-4.13 (m, 4H, OCH_2, CH_2O), 4.58 (m, 1H, ArCHO), 5.25 (m, 2H, CH_2=C), 5.98 (m, 1H, CH=C), 7.38-7.62 (m, 9H, ArH).

*2-(Allyloxy)-2-(4-benzylphenyl)ethan-1-ol (**43**)*

This compound was prepared starting from **22** following the procedure described for **40**: an oil was obtained (70% yield). ^1H NMR ($CDCl_3$): δ 2.33 (m, 1H, OH), 3.55-4.10 (m, 6H, OCH_2, CH_2O, OCH_2Ar), 4.48 (m, 1H, $ArCH_2O$), 5.22 (m, 2H, CH_2=C), 5.92 (m, 1H, CH=C), 7.18-7.39 (m, 9H, ArH).

2-(Allyloxy)-2-(4-(phenylthio)phenyl)ethan-1-ol (44)

This compound was prepared starting from **23**[34] following the procedure described for **40**: an oil was obtained (72% yield). ^{1}H NMR ($CDCl_3$): δ 2.38 (m, 1H, OH), 3.56-4.10 (m, 4H, OCH_2, CH_2O,), 4.48 (m, 1H, ArCHO), 5.22 (m, 2H, CH_2=C), 5.92 (m, 1H, CH=C), 7.18-7.42 (m, 9H, ArH).

3-((tert-Butyldimethylsilyl)oxy)-1,1-diphenylpropan-2-ol (46)

Et_3N (2.8 mL, 19 mmol) and TBDMSCl (1.5 g, 10 mmol) were added to a solution of 3,3-diphenylpropane-1,2-diol (**45**)[37] (2.3 g, 10 mmol) and 4-dimethylaminopyridine (DMAP) (0.12 g, 1 mmol) in dry CH_2Cl_2 (6 mL). After 11 h under stirring, EtOAc (20 mL) was added to the reaction mixture. The organic layer was washed with $KHSO_4$ 1M and brine. The organic layer was dried over anhydrous Na_2SO_4. The solvent was removed and the residue was purified by column chromatography on silica gel eluting with cyclohexane/EtOAc (9.7:0.3). An oil was obtained (79% yield). ^{1}H NMR ($CDCl_3$): δ 0.03 (s, 6H, $Si(CH_3)_2$), 0.92 (s, 9H, $C(CH_3)_3$), 2.48 (br s, 1H, OH), 3.42 (dd, 1H, OCH_2), 3.61 (dd, 1H, OCH_2), 4.07 (d, 1H, CHAr), 4.42 (m, 1H, CHO), 7.18-7.44 (m, 10H, ArH).

(2-(Allyloxy)-3,3-diphenylpropoxy)(tert-butyl)dimethylsilane (47)

A solution of **46** (1 g, 2.9 mmol) in dry THF (10 mL) was added dropwise to a suspension of 60% NaH (0.24 g, 6.0 mmol) in dry THF (10 mL) at room temperature. After 30 min, a solution of allyl bromide (0.45 g, 3.7 mmol) in THF (2 mL) was added dropwise. The reaction mixture was refluxed overnight. After cooling to room temperature water (10 mL) was added, the organic layer was evaporated to dryness and the residue was purified by column chromatography eluting with cyclohexane/EtOAc (9.8:0.2). An oil was obtained (77% yield). ^{1}H NMR ($CDCl_3$): δ 0.01 (s, 6H, $Si(CH_3)_2$), 0.89 (s, 9H, $C(CH_3)_3$); 3.58 (m, 2H, OCH_2), 3.85 (m, 1H, CHO); 4.09 (m, 2H, CH_2C=), 4.19 (d, 1H, CHAr), 5.06 (m, 2H, C=CH_2), 5.71 (m, 1H, CH=C), 7.15-7.44 (m, 10H, ArH).

(2R,5S*)-2-Cyclohexyl-5-(iodomethyl)-2-phenyl-1,4-dioxane (**50a**) and (2R*,5R*)-2-Cyclohexyl-5-(iodomethyl)-2-phenyl-1,4-dioxane (**50b**)*

These compounds were prepared starting from **40** following the procedure described for **28**. The isomer (**50a**) eluted first as a solid (12% yield); m.p. 104-106 °C. ^{1}H NMR ($CDCl_3$): δ 0.60-1.88 (m, 11H, cyclohexyl), 2.88 (m, 2H, CH_2I), 3.24 (dd, J = 11.5, 10.3 Hz, 1H, dioxane), 3.62-3.85 (m, 3H, dioxane), 4.61 (d, 1H, dioxane), 7.30-7.42 (m, 5H, ArH). The second fraction was the isomer **50b** as an oil (15% yield). ^{1}H NMR ($CDCl_3$): δ 0.61-2.28 (m, 11H, cyclohexyl), 3.33 (m, 2H, CH_2I), 3.54-3.91 (m, 4H, dioxane), 4.38 (d, 1H, dioxane), 7.22-7.40 (m, 5H, ArH).

(2R,5S*)-2-Benzhydryl-5-(iodomethyl)-1,4-dioxane (**51a**) and (2S*,5S*)-2-Benzhydryl-5-(iodomethyl)-1,4-dioxane (**51b**)*

These compounds were prepared starting from **41** following the procedure described for **28**. The *trans* isomer (**51b**) eluted first as a solid (13% yield); m.p. 131-133 °C. ^{1}H NMR ($CDCl_3$): δ 3.08 (m, 2H, CH_2I), 3.38-3.58 (m, 3H, dioxane), 3.69 (dd, 1H, dioxane), 3.89 (d, 1H, CHAr), 4.01 (dd, 1H, dioxane), 4.27 (m, 1H, dioxane), 7.08-7.38 (m, 10H, ArH). The second fraction was the isomer **51a** as an oil (13% yield). ^{1}H NMR ($CDCl_3$): δ 3.38 (m, 2H, CH_2I), 3.52-3.91 (m, 6H, dioxane, CHAr), 4.28 (m, 1H, dioxane), 7.13-7.42 (m, 10H, ArH).

(2R,5S*)-2-([1,1'-Biphenyl]-4-yl)-5-(iodomethyl)-1,4-dioxane (**52a**) and (2S*,5S*)-2-([1,1'-Biphenyl]-4-yl)-5-(iodomethyl)-1,4-dioxane (**52b**)*

These compounds were prepared starting from **42** following the procedure described for **28**. The *trans* isomer (**52b**) eluted first as a solid (8% yield); m.p. 175-176 °C. ^{1}H NMR ($CDCl_3$): δ 3.19 (d, 2H, CH_2I), 3.35-3.81 (m, 3H, dioxane), 4.02 (dd, 1H, dioxane), 4.20 (dd, 1H, dioxane), 4.63 (dd, 1H, dioxane), 7.32-7.62 (m, 9H, ArH). The second fraction was the isomer **52a** as a solid (6% yield);

m.p. 136-137 °C. ^{1}H NMR ($CDCl_3$): δ 3.57 (m, 2H, CH_2I), 3.78-4.10 (m, 5H, dioxane), 4.68 (dd, 1H, dioxane), 7.28-7.43 (m, 9H, ArH).

*(2R**,*5S***)-2-(4-Benzylphenyl)-5-(iodomethyl)-1,4-dioxane (**53a**) and (2S**,*5S***)-2-(4-Benzylphenyl)-5-(iodomethyl)-1,4-dioxane (**53b**)*

These compounds were prepared starting from **43** following the procedure described for **28**. The *trans* isomer (**53b**) eluted first as a solid (21% yield); m.p. 110-111 °C. ^{1}H NMR ($CDCl_3$): δ 3.17 (m, 2H, CH_2I), 3.49-3.78 (m, 3H, dioxane), 3.95 (dd, 1H, dioxane), 4.00 (s, 2H, CH_2Ar); 4.18 (dd, 1H, dioxane), 4.56 (dd, 1H, dioxane), 7.18-7.35 (m, 9H, ArH). The second fraction was the isomer **53a** as a solid (9% yield); m.p. 88-90 °C. ^{1}H NMR ($CDCl_3$): δ 3.58 (m, 2H, CH_2I), 3.67-4.13 (m, 7H, dioxane, CH_2Ar), 4.61 (dd, 1H, dioxane), 7.15-7.38 (m, 9H, ArH).

*(2S**,*5R***)-2-(Iodomethyl)-5-(4-(phenylthio)phenyl)-1,4-dioxane (**54a**) and (2S**,*5S***)-2-(Iodomethyl)-5-(4-(phenylthio)phenyl)-1,4-dioxane (**54b**)*

These compounds were prepared starting from **44** following the procedure described for **28**. The *trans* isomer (**54b**) eluted first as a solid (20% yield); m.p. 96-97 °C. ^{1}H NMR ($CDCl_3$): δ 3.16 (m, 2H, CH_2I), 3.50-3.77 (m, 3H, dioxane), 3.97 (dd, 1H, dioxane), 4.17 (dd, 1H, dioxane), 4.55 (dd, 1H, dioxane), 7.22-7.39 (m, 9H, ArH). The second fraction was the isomer **54a** as a solid (16% yield); m.p. 80-81 °C. ^{1}H NMR ($CDCl_3$): δ 3.52 (m, 2H, CH_2I), 3.72-4.08 (m, 5H, dioxane), 4.61 (dd, 1H, dioxane), 7.26-7.40 (m, 9H, ArH).

*(2S**,*5R***)-2-(Iodomethyl)-5-(4-(phenylsulfinyl)phenyl)-1,4-dioxane (**55a**)*

This compound was prepared starting from **54a** following the procedure described for **31a**: a solid was obtained (85% yield); m.p. 90-91 °C. ^{1}H NMR ($CDCl_3$): δ 3.50 (m, 2H, CH_2I), 3.72-4.00 (m, 5H, dioxane), 4.62 (dd, 1H, dioxane), 7.42-7.69 (m, 9H, ArH).

(2S,5S*)-2-(Iodomethyl)-5-(4-(phenylsulfinyl)phenyl)-1,4-dioxane (**55b**)*

This compound was prepared starting from **54b** following the procedure described for **31a**: an oil was obtained (71% yield). ^{1}H NMR ($CDCl_3$): δ 3.15 (m, 2H, CH_2I), 3.43-375 (m, 3H, dioxane), 3.94 (dd, 1H, dioxane), 4.18 (dd, 1H, dioxane), 4.59 (dd, 1H, dioxane), 7.39-7.68 (m, 9H, ArH).

*(2S,5R)-2-(Iodomethyl)-5-(4-(phenylsulfonyl)phenyl)-1,4-dioxane (**56a**)*

This compound was prepared starting from **54a** following the procedure described for **32a**: a solid was obtained (74% yield); m.p. 152-153 °C ^{1}H NMR ($CDCl_3$): δ 3.45 (m, 2H, CH_2I), 3.74-4.00 (m, 5H, dioxane), 4.62 (dd, 1H, dioxane), 7.45-7.98 (m, 9H, ArH).

(2S,5S*)-2-(Iodomethyl)-5-(4-(phenylsulfonyl)phenyl)-1,4-dioxane (**56b**)*

This compound was prepared starting from **54b** (0.90 g, 2.13 mmol) following the procedure described for **32a**: a solid was obtained (77% yield); m.p. 125-126. ^{1}H NMR ($CDCl_3$): δ 3.18 (d, 2H, CH_2I), 3.41-3.73 (m, 3H, dioxane), 3.97 (dd, 1H, dioxane), 4.19 (dd, 1H, dioxane), 4.61 (dd, 1H, dioxane), 7.41-7.99 (m, 9H, ArH).

*2-(Oxiran-2-ylmethoxy)-1,2-diphenylethan-1-ol (**67**)*

m-CPBA (50%) (14.14 g, 41.0 mmol) was added to a solution of **66** (4.9 g, 19.0 mmol) in CH_2Cl_2 (100 mL). After 20 h at room temperature under stirring the reaction mixture was washed with 10% Na_2SO_3, 5%Na_2CO_3, and H_2O. Removal of dried solvents afforded a mixture of two diastereomers (6:4) as an oil (70% yield). ^{1}H NMR ($CDCl_3$): δ 4.01 (br s, 1H, OH), 2.51 (m, 1H A + 1H B, dioxane), 2.72 (m, 1H A + 1H B, dioxane), 3.07 (m, 1H A + 1H B, dioxane), 3.20-3.72 (m, 2H A + 2H B, OCH_2), 4.52 (d, 1H A, OCHAr), 4.56 (d, 1H B, OCHAr), 4.91 (d, 1H A, ArCHO), 4.56 (d, 1H B, ArCHO), 7.07-8.18 (m, 10H A + 10H B, ArH).

((2R,5S*,6R*)-5,6-Diphenyl-1,4-dioxan-2-yl)methanol (**68c**)*

CF_3COOH (0.8 mL) was added to a solution of **67** (2.5 g, 9.3 mmol) in $CHCl_3$ (100 mL). After 24 h under stirring at room temperature, the reaction mixture was washed with $NaHCO_3$ saturated solution and dried over Na_2SO_4. The organic layer was evaporated to dryness, and the residue was purified by column chromatography on silica gel eluting with cyclohexane/EtOAc (8:2) to afford a colorless oil (79% yield). ^1H NMR ($CDCl_3$): δ 1.92 (br s, 1H, OH, exchangeable with D_2O), 3.19-4.47 (m, 6H, CH_2O, dioxane), 4.80 (m, 1H, dioxane), 7.00-7.52 (m, 10H, ArH).

(2S,3R*,5S*)-5-(Iodomethyl)-2,3-diphenyl-1,4-dioxane (7**0**)*

This compound was prepared starting from **66**[39] following the procedure described for **28**: a solid was obtained (40% yield); m.p. 132-134 °C. ^1H NMR ($CDCl_3$): δ 3.09 (m, 2H, CH_2I), 3.72-3.82 (m, 2H, dioxane), 4.38 (d, 1H, dioxane), 5.19 (m, 2H, dioxane), 7.11-7.51 (m, 10H, ArH).

*2-(Allyloxy)-1,1,2-triphenylethan-1-ol (7**2**)*

A solution of phenylmagnesium chloride 2 M in THF (10.3 mL, 20.6 mmol) was added to a solution of **71**[40] (2.6 g, 10.3 mmol) in ether (50 mL) at -78 °C, and the mixture was stirred until the starting material was completely consumed (30 min). The mixture was poured onto aqueous NH_4Cl solution, the organic layer was separated, and the aqueous layer was extracted with ether. The combined organic extracts were dried over Na_2SO_4, filtered, and evaporated. The residue was purified by flash chromatography eluting with cyclohexane/EtOAc (9.8:0.2): a solid was obtained (76% yield); m.p. 83-85 °C. ^1H NMR ($CDCl_3$): δ 3.05 (br s, 1H, OH), 3.82-4.12 (m, 2H, OCH_2), 5.08-5.22 (m, 3H, CH_2=C and CHAr), 5.91 (m, 1H, CH=C), 6.98-7.63 (m, 15, ArH).

*2-(Oxiran-2-ylmethoxy)-1,1,2-triphenylethan-1-ol (7**3**)*

This compound was prepared starting from **72** following the procedure described for **67**: a mixture of two diastereomers (8:2) was obtained (87% yield). ^1H NMR ($CDCl_3$): δ 1.22 (br s, 1H A + 1H B,

OH), 2.40 (m, 1H A, dioxane), 2.47 (m, 1H B, dioxane), 2.73 (m, 1H A + 1H B, dioxane), 3.12 (m, 1H A + 1H B, dioxane), 3.27 (dd, 1H A, OCH_2), 3.42 (dd, 1H B, OCH_2), 3.66 (dd, 1H B, OCH_2), 3.78 (dd, 1H A, OCH_2), 5.21 (s, 1H B, OCHAr), 5.35 (s, 1H A, OCHAr), 6.98-8.08 (m, 15H A + 15H B, ArH).

((2R,5S*)-5,6,6-Triphenyl-1,4-dioxan-2-yl)methanol (74**a**) and ((2R*,5R*)-5,6,6-Triphenyl-1,4-dioxan-2-yl)methanol (74**b**)*

These compounds were prepared starting from **73** following the procedure described for **68c**. The diastereoisomer **74a** eluted first as a solid (15% yield); m.p. 58-59 °C. ^1H NMR ($CDCl_3$): δ 2.00 (br s, 1H, OH, exchangeable with D_2O), 3.61-4.18 (m, 5H, CH_2O, dioxane), 4.99 (s, 1H, dioxane), 6.67-7.66 (m, 15, ArH). The second fraction was the diastereoisomer **74b** as a solid (20% yield); m.p. 65-67 °C. ^1H NMR ($CDCl_3$): δ 1.63 (br s, 1H, OH, exchangeable with D_2O), 3.40 (dd, 1H, dioxane), 3.62-4.06 (m, 4H, CH_2O, dioxane), 5.83 (s, 1H, dioxane), 6.89-7.75 (m, 15H, ArH).
